# Prevalence and epidemiological patterns of *Neisseria gonorrhoeae* infection in Canada, 1969–2025: a systematic review and meta-analysis

**DOI:** 10.1186/s12889-026-26579-y

**Published:** 2026-03-03

**Authors:** Manale Harfouche, Ammar Khan, Amr Taha, Rayane El-Khoury, Rwedah A. Ageeb, Laith J. Abu-Raddad

**Affiliations:** 1 Infectious Disease Epidemiology Group, Weill Cornell Medicine-Qatar, Cornell University, Qatar Foundation - Education City, Doha, Qatar; 2 Department of Population Health Sciences, Weill Cornell Medicine, Cornell University, New York, NY USA; 3 Department of Public Health, College of Health Sciences, QU Health, Qatar University, Doha, Qatar; 4 College of Health and Life Sciences, Hamad bin Khalifa University, Doha, Qatar

**Keywords:** Gonorrhea, Sexually transmitted infection, Antimicrobial resistance, Prevalence, Meta-analytics

## Abstract

**Background:**

*Neisseria gonorrhoeae* (NG) infection remains a concern due to its morbidity and increasing antimicrobial resistance. This study synthesized evidence on NG prevalence in Canada, examining epidemiological patterns across populations and anatomical sites, temporal trends, and key associations.

**Methods:**

A systematic review of NG prevalence studies published up to August 7, 2025, was conducted following the Cochrane framework and reported in accordance with PRISMA guidelines. Random-effects meta-analyses generated pooled prevalence estimates, while random-effects meta-regression analyses identified associations and sources of heterogeneity.

**Results:**

A total of 136 publications contributed 226 prevalence measures spanning 1969–2025. In the general population, the pooled mean prevalence of urogenital infection was 1.0% (95% CI: 0.5–1.5%). Among men who have sex with men, pooled prevalence was 0.9% (95% CI: 0.2–1.9%) for urogenital, 2.6% (95% CI: 0.9–4.9%) for anorectal, and 1.6% (95% CI: 0.2–3.8%) for oropharyngeal infection. Among STI clinic attendees, prevalence was 7.5% (95% CI: 4.2–11.6%) for urogenital, 2.5% (95% CI: 0.7–5.3%) for anorectal, and 5.0% (95% CI: 1.8–9.6%) for oropharyngeal infection. Prevalence was high among symptomatic populations—15.9% (95% CI: 4.0–33.2%) in women and 37.4% (95% CI: 11.1–68.6%) in men. Meta-regression analyses explained over half of the variation in prevalence, showing a long-term decline, a gradient by population type, and a small-study effect, but no differences by age, sex, or province.

**Conclusions:**

NG prevalence in Canada mirrors global levels and patterns. The persistence of infection underscores the need for sustained prevention, enhanced surveillance—including extragenital screening—and continued research to inform policy.

**Supplementary Information:**

The online version contains supplementary material available at 10.1186/s12889-026-26579-y.

## Background

Gonorrhea, caused by *Neisseria gonorrhoeae* (NG), remains one of the most common sexually transmitted infections (STI) worldwide [1–3]. NG can infect the urogenital, anorectal, and oropharyngeal mucosa [1, 4]. Efforts to control its spread are hindered by the prevalence of asymptomatic infection—particularly among women—which contributes to delayed diagnosis, untreated cases, and ongoing transmission [1, 4]. Left untreated, NG infection can cause serious complications, including urethritis, cervicitis, pelvic inflammatory disease, ectopic pregnancy, and infertility [1, 4, 5]. The World Health Organization (WHO) estimated 82.4 million new NG infections globally in 2020 [6, 7], and recent evidence points to increasing incidence across different countries [8].

Gonorrhea poses a global health challenge due to widespread antimicrobial resistance (AMR) and the emergence of extensively drug-resistant strains [1, 9, 10]. Recognizing this threat, WHO has classified gonococcal AMR as a global high-priority concern and launched a dedicated action plan to address it [11]. In parallel, WHO’s Global Health Sector Strategy on HIV, Viral Hepatitis, and STIs aims to achieve a 90% reduction in the global incidence of NG infection by 2030 relative to the baseline incidence in 2020, through expanded access to quality care and evidence-based interventions [12]. A pillar of this strategy is strengthening understanding of STI epidemiology to mobilize political commitment, inform national planning, optimize resource allocation, enhance program effectiveness, and support the potential future introduction of an NG vaccine [12–14].

NG infection is the second most commonly reported STI in Canada, following chlamydia [15, 16]. The number of reported NG cases has increased in recent years across provinces and demographic groups [15, 16]. This increase may reflect changes in transmission dynamics, sexual behaviors, and inequities in access to comprehensive sexual health services [15–17]. Against this background, the present study aimed to characterize the epidemiology of NG infection in Canada over the past four decades by: (1) systematically reviewing and synthesizing available prevalence data; (2) estimating pooled mean prevalence across population groups; and (3) examining population-level associations, temporal trends, and sources of between-study heterogeneity.

## Methods

### Data sources and search strategy

A systematic review was undertaken to synthesize epidemiologic evidence on NG prevalence in Canada, following methodological guidance from the Cochrane Collaboration [18]. Findings are presented in accordance with the Preferred Reporting Items for Systematic Reviews and Meta-Analyses (PRISMA) guidelines [19], with the corresponding checklist provided in Supplementary Table S1. The review protocol was not formally registered with PROSPERO, as it was adapted from a previously published protocol [20] and informed by established methodological frameworks applied in previous systematic reviews of NG and other STI prevalence [5, 21–27].

A literature search was conducted across four international databases—Embase, PubMed, Scopus, and Web of Science—covering all publications up to August 7, 2025, the date of the most recent update of this review. To maximize coverage and minimize the risk of missing relevant studies, a broad and systematic search strategy was applied in each database, combining exploded index terms (including all subheadings) with relevant free-text keywords (Supplementary Table S2). No restrictions were imposed on language or publication year.

### Study selection process and inclusion and exclusion criteria

Search results were imported into EndNote (Clarivate Analytics, London, United Kingdom) for deduplication. Screening was conducted independently by three reviewers (MH, AK, and AT). Titles and abstracts were first screened to identify relevant or potentially relevant records, followed by full-text assessment of selected articles. Reference lists of eligible studies and relevant reviews were also examined to capture additional records. Discrepancies during the screening process were resolved by consensus, with arbitration from the senior study author (LJA).

Publications were eligible for inclusion if they reported primary data on NG prevalence in Canada based on specimens collected directly from study participants, including urogenital, rectal, and oropharyngeal specimens. Specimens were required to be tested with laboratory methods such as nucleic acid amplification tests (NAATs)/polymerase chain reaction (PCR), culture, or Gram stain. Studies were excluded if they relied on self-reported infection status, included fewer than 10 participants, or tested specimens from the upper genital tract. Case reports, case series, commentaries, reviews, and qualitative studies were also excluded.

For this review, specific definitions were applied to distinguish between types of retrieved information. A *record* was defined as any document—such as a publication or public health report—reporting NG prevalence data for one or more distinct populations. A *study* referred to a single prevalence estimate extracted from a record for any population type, irrespective of the sampling method used.

When duplicate prevalence estimates were identified across multiple records, a predefined hierarchy was used to select the most appropriate source: first, the record providing the most recent NG prevalence was retained; second, if publication years were identical, the record with the largest sample size was selected; and third, if both year and sample size were identical, the record offering the most detailed data for extraction and analysis was prioritized.

### Data extraction

Data extraction from eligible publications was conducted by at least two reviewers (MH, AK, AT, REK, and RAA), who performed both independent and double extraction of all overall (total sample) and stratified outcome measures. Extraction variables were predefined, piloted, and are summarized in Supplementary Box S1. When prevalence estimates were reported for combined sexes, sex classification was assigned based on the predominant sex in the sample (≥ 60%). Prevalence data for individuals younger than 15 years were extracted but excluded from analysis.

Stratified data were extracted only when subgroup sample sizes were ≥ 10 participants. Prevalence estimates of current NG infection were stratified according to the following hierarchy: anatomical site (urogenital, anorectal, or oropharyngeal), population type, sex, year of data collection, age group, and region or city. Study populations were classified according to risk of exposure to NG infection and symptomatic status, as delineated in Table 1, which also provides definitions for each population type. In addition to measures of current NG infection, serologic measures reflecting ever infection were also extracted.

**Table 1:** Definitions of population type categories

Population	Definition
General populations (low-risk populations)	Groups considered at low risk of exposure to NG, including antenatal clinic attendees, blood donors, pregnant women, and other comparable populations.
Intermediate-risk populations	Groups presumed to have sexual contact with individuals engaging in high-risk sexual behavior, resulting in a greater risk of NG exposure than the general population. Examples include prisoners, homeless people, people who inject drugs, migrant workers, and truck drivers.
Female sex workers	Women who engage in sex work, defined as the exchange of sexual services for money.
Men who have sex with men	Men who engage in same-sex sexual activity, particularly anal intercourse with other men.
Symptomatic women	Women with clinical manifestations related to NG infection, such as presenting with vaginal discharge.
Symptomatic men	Men with clinical manifestations related to NG infection, such as presenting with urethral discharge.
Symptomatic mixed sexes	Populations of undetermined sex with clinical manifestations related to NG infection, such as vaginal or urethral discharge.
Infertility clinic attendees	Categorized separately due to uncertainty regarding their risk of NG exposure and the potential biological association between NG infection and infertility.
Women with miscarriage or ectopic pregnancy	Categorized separately due to uncertainty regarding their risk of NG exposure and the potential biological association between NG infection and miscarriage or ectopic pregnancy.
STI clinic attendees	Individuals attending or seeking care at STI clinics.
Individuals living with HIV and individuals in HIV-discordant couples	Individuals living with HIV or those in a spousal relationship with an individual living with HIV.
Sexual contacts of persons with NG or CT infection	Individuals who have had sexual contact with persons infected with NG or CT.
Patients with confirmed or suspected STIs and related infections	Individuals diagnosed with an STI or suspected of having concomitant STIs or other related infections.
Other populations	Groups that do not fit the above definitions or have an undetermined risk of acquiring NG infection, such as cervical cancer patients, specimens submitted to virology or bacteriology laboratories, and mixed or undefined populations.

*CT* *Chlamydia trachomatis,* *HIV* Human immunodeficiency virus, *NG* *Neisseria gonorrhoeae,* *STI* Sexually transmitted infection

When a single assay was used to test multiple specimen types within a study, only one prevalence estimate was retained—prioritizing endocervical swabs for women (followed by vaginal swabs and urine samples) and urethral swabs for men (followed by urine and semen samples). Conversely, when different assays were applied to the same specimen type, each estimate was extracted separately. This approach enabled evaluation of the influence of diagnostic methods on NG prevalence through meta-regression analyses and supported the derivation of adjustment factors for STI estimation in mathematical modeling studies [28, 29].

Diagnostic performance for NG detection varies across assay types, with NAAT/PCR demonstrating higher sensitivity than culture or Gram stain [30–32]. Accordingly, extracting separate prevalence estimates when different assays were applied to the same specimen type enabled assessment of the impact of assay choice on reported NG prevalence through meta-regression analyses, as described below. This approach also supported the derivation of adjustment factors for STI estimation in mathematical modeling studies [28, 29].

### Precision, risk of bias, and publication bias

The precision and risk of bias of included studies were independently evaluated by at least two authors (MH, AK, AT, REK, and RAA), with input from LJA. Assessments followed the Cochrane framework [18], incorporated quality criteria tailored to prevalence studies [33, 34], and were refined based on approaches used in previous systematic reviews of STI prevalence [21–27, 35]. The final evaluation framework comprised one criterion assessing study precision and two assessing risk of bias.

Additional criteria were not formally evaluated, as they were either inherently addressed through the review’s design and eligibility criteria or more appropriately examined in subsequent analyses within the study (Supplementary Table S3). For example, the validity and reliability of diagnostic assays used to estimate NG prevalence were assessed through meta-regression analyses evaluating the effect of assay type on prevalence estimates.

Study precision was classified as low (< 200 participants) or high (≥ 200 participants). Risk of bias was assessed as low or high based on sampling methodology (probability-based vs. non-probability-based) and response rate (≥ 80% vs. < 80%). When information was insufficient, the risk of bias was rated as unclear. These quality indicators were incorporated into meta-regression analyses to assess their potential impact on NG prevalence estimates, consistent with approaches used in previous systematic reviews [21–27, 35].

Publication bias was evaluated using Doi plots and the Luis Furuya-Kanamori (LFK) index when at least three studies were available [36]. Asymmetry in the Doi plot was interpreted as evidence of potential publication bias, suggesting that variation in prevalence estimates may not be fully attributable to random error [36]. An absolute LFK index value greater than 1 was considered indicative of possible publication bias [36].

### Meta-analyses

Stratified prevalence estimates were summarized using medians and ranges. Meta-analyses of stratified NG prevalence were performed using DerSimonian–Laird random-effects models [37], applying the Freeman–Tukey double arcsine transformation to stabilize variance [38] after confirming its suitability for the data [39]. Pooled mean prevalence estimates and corresponding 95% confidence intervals (CIs) were generated for each population type, stratified by anatomical site and assay type, when at least three observations were available. Pooled results and between-study variability were displayed using forest plots.

Heterogeneity was assessed using Cochran’s Q statistic, with a p-value < 0.1 indicating significant heterogeneity across studies. The I² statistic quantified the proportion of total variation attributable to true differences in prevalence rather than sampling error, while prediction intervals were calculated to reflect the expected range of true prevalence values around the pooled mean [40]. All analyses were conducted in R version 4.1.3 (R Foundation for Statistical Computing, Vienna, Austria) using the *meta* package [41].

Given the heterogeneity observed across prevalence estimates, pooled means were interpreted as summary indicators representing the overall average across studies [24, 27]. To better understand this heterogeneity, meta-regression analyses were conducted to identify epidemiologic and methodological factors associated with differences in NG prevalence across studies.

### Meta-regressions

Univariable and multivariable random-effects meta-regression analyses were performed to explore sources of between-study heterogeneity and identify factors associated with NG prevalence. Log-transformed prevalence estimates were used [42], with the log transformation preferred over the logit transformation to facilitate the estimation of prevalence ratios (PRs), which provide a more epidemiologically interpretable measure of association than prevalence odds ratios [25].

Selection of variables for inclusion in the meta-regression models was guided by epidemiologic relevance, prior evidence from HIV/STI research, and the need to assess potential sources of bias in the available prevalence estimates [21–27, 35]. Candidate variables are detailed in Supplementary Box S2. Variables with a p-value ≤ 0.2 in univariable analyses were considered for inclusion in the multivariable model, in which a p-value ≤ 0.05 indicated a statistically significant association with NG prevalence. Model performance was assessed using the adjusted R² to quantify the proportion of between-study heterogeneity explained.

The meta-regression analyses incorporated assessments of temporal trends by examining the effects of publication year and year of data collection on observed prevalence. These variables were modeled both categorically and as continuous linear terms, with the latter used to evaluate the presence of an overarching temporal trend across the decades in which the prevalence studies were conducted.

For studies that did not report the year of data collection, this value was imputed as the publication year minus the median difference between publication year and data collection year among studies reporting both. This approach maintained the temporal structure of the data while minimizing potential bias.

Meta-regression analyses were conducted in Stata/SE version 16 (StataCorp, College Station, TX, USA) using the *metareg* package [42].

### Ethics

This study was conducted in accordance with established ethical guidelines and principles. As all data were obtained from publicly available sources, and no primary data collection involving human participants was conducted, formal ethics approval was not required.

### Role of the funding source

The funder of the study had no role in study design, data collection, data analysis, data interpretation, or writing of the article. MH and LJA had full access to all the data in the study and had the final responsibility for the decision to submit for publication.

## Results

### Search results and scope of evidence

**Fig. 1:**
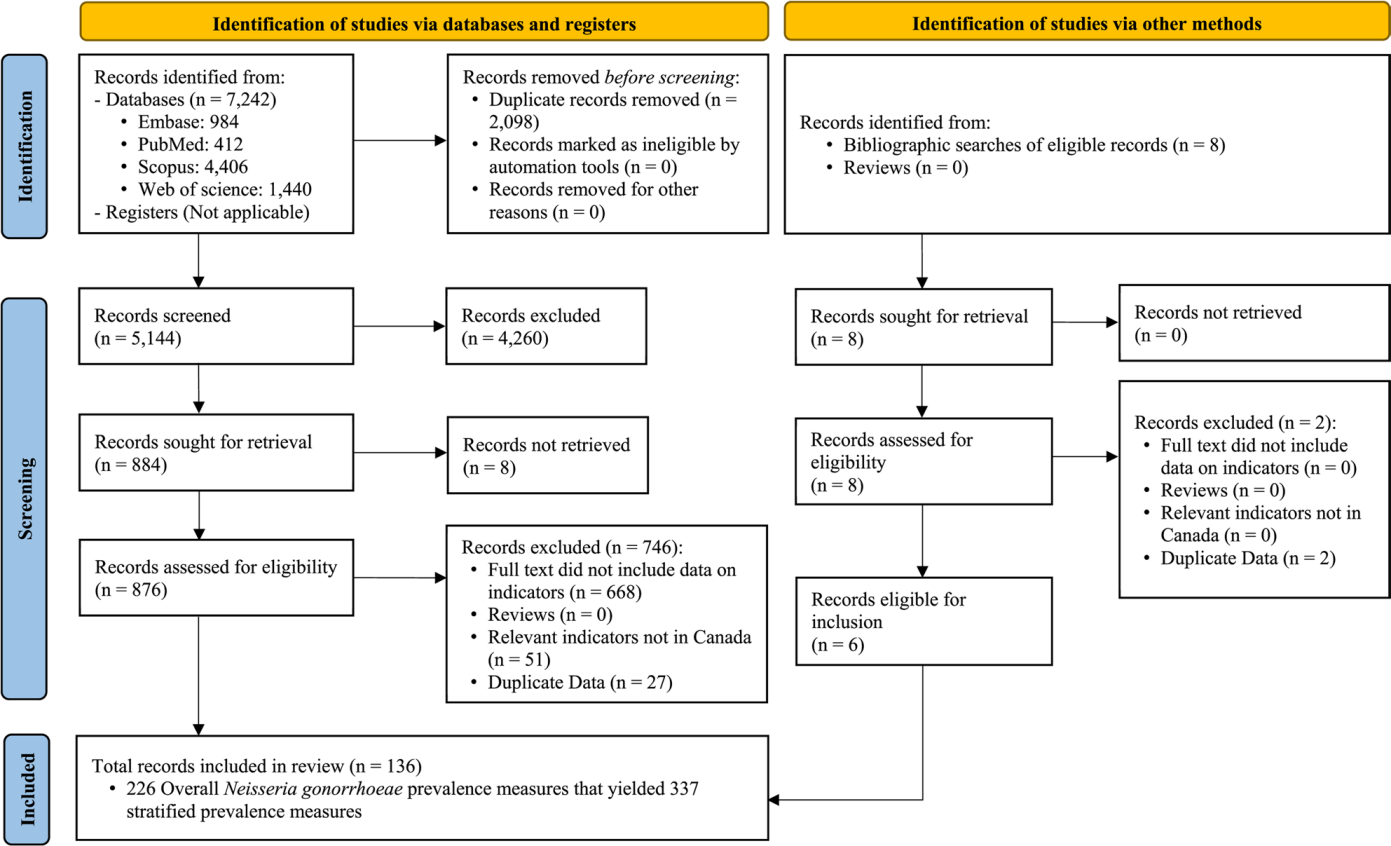
Study selection flowchart for *Neisseria gonorrhoeae* prevalence in Canada, following PRISMA guidelines [19]

Figure 1 presents the PRISMA flow diagram summarizing the study selection process. The systematic search identified 7,242 records: 984 from Embase, 412 from PubMed, 4,406 from Scopus, and 1,440 from Web of Science. After removing 2,098 duplicates, 5,144 unique records were screened by title and abstract, resulting in the exclusion of 4,260 records. The remaining 884 full-text articles were screened in detail, and 130 met the inclusion criteria. No reviews were identified in the search to provide additional records. Bibliographic screening of relevant publications identified an additional six eligible studies. In total, 136 publications were included in the review (Supplementary Table S4).

From the included publications, a total of 226 overall (total-sample) NG prevalence measures and 337 stratified measures were extracted. The overall measures comprised 146 urogenital (181 stratified), 16 anorectal (20 stratified), 11 oropharyngeal (15 stratified), 43 unspecified-site (111 stratified), and 10 serologic estimates.

The extracted data spanned several decades, with the earliest measure reported in 1969, and over one-quarter of all overall measures (*n* = 64) published after 2015. Ontario (*n* = 71) and Quebec (*n* = 46) contributed the largest number of measures.

### Precision, risk of bias, and publication bias assessments

Supplementary Table S5 presents the assessments of precision and risk of bias for all included studies. Among the 226 studies reporting NG prevalence, 154 (68.1%) had sample sizes of at least 200 participants, indicating high precision, while 72 (31.9%) were classified as low precision.

Nearly all studies (219, 96.9%) employed non-probability sampling methods and were therefore classified as having a high risk of bias. Reporting of response rates was limited: 201 studies (88.9%) did not report response rates and were thus assigned an unclear risk of bias for this domain. Thirty-one studies (13.7%) were classified as having a low risk of bias in at least one domain. None of the studies demonstrated low risk of bias across both sampling method and response rate domains, and only one study exhibited high risk of bias in both.

Publication bias assessments are summarized in Supplementary Table S6. While few meta-analyses showed no evidence of publication bias, others displayed asymmetrical Doi plots and absolute LFK index values exceeding 1, suggesting potential publication bias (Supplementary Figures S1–S5).

### Pooled mean estimates of NG prevalence

Table 2 summarizes the stratified NG prevalence estimates by median and range and presents pooled mean prevalence by population type and anatomical site, with additional stratification by assay type provided in Supplementary Table S7.

**Table 2:** Pooled mean prevalence of Neisseria gonorrhoeae in Canada, stratified by population type and anatomical site

Population type by anatomical site	Stratified prevalence measures	Sample size	NG prevalence (%)	Pooled mean NG prevalence	Heterogeneity measures			
	Total n	Total N	Range	Median	Mean (%) (95% CI)	Q^*^ (*p*-value)	I²^†^ (%) (95% CI)	Prediction interval^§^ (%)
General populations								
Urogenital	53	353,423	0.0–11.9	0.8	1.0 (0.5–1.5)	3496.0 (*p*<0.001)	98.5 (98.4–98.7)	0.0–6.6
Serological	5	1231	0.0–7.0	1.5	2.3 (0.3–5.6)	21.4 (*p*<0.001)	81.3 (56.5–91.9)	0.0–14.6
Unspecified/mixed	79	220,432	0.0–3.1	0.3	0.5 (0.4–0.6)	1523.7 (*p*<0.001)	94.9 (94.1–95.5)	0.0–2.3
Intermediate-risk populations								
Urogenital	35	12,162	0.0–18.0	1.7	2.7 (1.4–4.3)	526.0 (*p*<0.001)	93.5 (91.9–94.8)	0.0–15.6
Unspecified/mixed	4	5821	3.4–12.9	4.0	3.3 (2.7–3.9)	6.3 (*p*=0.098)	52.3 (0.0–84.2)	1.7–5.3
Men who have sex with men								
Urogenital	14	5373	0.0–8.3	0.5	0.9 (0.2–1.9)	91.2 (*p*<0.001)	85.8 (77.7–90.9)	0.0–6.2
Anorectal	13	3378	0.0–10.9	2.9	2.6 (0.9–4.9)	99.4 (*p*<0.001)	87.9 (81.1–92.3)	0.0–14.1
Oropharyngeal	12	4071	0.0–11.0	2.0	1.6 (0.2–3.8)	49.5 (*p*<0.001)	93.5 (90.4–95.6)	0.0–13.9
Unspecified/mixed	7	12,369	1.0–43.9	9.1	10.0 (2.4–21.8)	2717.9 (*p*<0.001)	99.8 (99.7–99.8)	0.0–64.0
Symptomatic women								
Urogenital	9	901	0.0–72.8	16.7	15.9 (4.0–33.2)	383.3 (*p*<0.001)	97.9 (97.1–98.5)	0.0–83.8
Symptomatic men								
Urogenital	4	589	22.0–82.6	22.9	37.4 (11.1–68.6)	217.2 (*p*<0.001)	98.6 (97.3–99.1)	0.0–100.0
Serological	1	205	-	-	2.9 (1.1–6.3)	-	-	-
Infertility clinic attendees								
Urogenital	5	5589	0.0–0.1	0.0	0.0 (0.0-0.0)	0.9 (*p*=0.925)	0.0 (0.0–79.2)	0.0-0.0
Women with miscarriage or ectopic pregnancy								
Urogenital	1	52	-	-	0.0 (0.0–6.84)	-	-	-
STI clinic attendees								
Urogenital	26	78,622	0.1–47.6	4.2	7.5 (4.2–11.6)	1822.7 (*p*<0.001)	98.6 (98.4–98.8)	0.0–37.3
Anorectal	5	3500	0.2–5.9	2.8	2.5 (0.7–5.3)	57.9 (*p*<0.001)	93.1 (86.8–96.4)	0.0–15.1
Oropharyngeal	3	2988	2.4–8.9	5.9	5.0 (1.8–9.6)	17.9 (*p*<0.001)	88.8 (69.2–95.9)	0.0–31.2
Serological	1	156	-	-	35.3 (27.8–43.3)	-	-	-
Unspecified/mixed	9	24,419	0.4–21.2	9.9	7.6 (3.3–13.3)	1922.8 (*p*<0.001)	99.6 (99.5–99.7)	0.0-34.7
Individuals living with HIV and individuals in HIV-discordant couples								
Urogenital	5	3702	0.0–2.0	0.3	0.6 (0.0–1.8)	10.2 (*p*=0.037)	60.9 (0.0–85.3)	0.0–4.6
Unspecified/mixed	2	2575	0.7–2.3	1.5	1.9 (1.4–2.6)	-	-	-
Sexual contacts of persons infected with NG or CT infection								
Urogenital	1	133	-	-	47.4 (38.7–56.2)	-	-	-
Unspecified/mixed	2	429	25.0-25.0	25.0	20.3 (16.6–24.4)	-	-	-
Patients with confirmed or suspected STIs and related infections								
Anorectal	2	432	2.6–13.3	8.0	8.6 (6.1–11.6)	-	-	-
Serological	1	169	-	-	79.9 (73.0–85.6)	-	-	-
Unspecified/mixed	6	17,636	10.3–24.0	13.7	14.1 (10.2–18.4)	112.1 (*p*<0.001)	95.5 (92.6–97.3)	3.3–30.4
Other populations^¶^								
Urogenital	28	19,463	0.0–47.2	6.7	7.2 (4.5–10.4)	1046.7 (*p*<0.001)	97.4 (96.9–97.9)	0.0–29.6
Serological	2	885	14.3–19.4	7.9	19.2 (16.7–22.0)	-	-	-
Unspecified/mixed	2	19,829	7.6–15.3	11.4	11.5 (5.1–19.9)	-	-	

*Abbreviation*: *CI* Confidence interval, *CT* Chlamydia trachomatis, *HIV* Human immunodeficiency virus, *NG* Neisseria gonorrhoeae, *STI* Sexually transmitted infection

^*^Q: The Cochran’s Q statistic is a measure assessing the existence of heterogeneity in pooled outcome measures, here NG prevalence

^+^I^2^: A measure that assesses the magnitude of between-study variation that is due to true differences in NG prevalence across studies rather than chance

^§^Prediction interval: A measure that estimates the distribution (95% interval) of true NG prevalence around the estimated mean

^¶^Other populations include groups with an undetermined risk of acquiring NG infection, such as cervical cancer patients, individuals evaluated following sexual assault, specimens submitted to virology or bacteriology laboratories, and mixed or undefined populations

A minimum of three studies was required to perform a meta-analysis

Forest plots illustrating pooled prevalence estimates across population groups are presented in Supplementary Figure S6 for urogenital infection, Supplementary Figure S7 for anorectal infection, Supplementary Figure S8 for oropharyngeal infection, Supplementary Figure S9 for serological evidence of ever infection, and Supplementary Figure S10 for infections detected in unspecified or mixed specimens.

In the general population, the pooled mean prevalence of urogenital NG infection was 1.0% (95% CI: 0.5–1.5%), while serological measures indicated an ever-infection prevalence of 2.3% (95% CI: 0.3–5.6%). Among intermediate-risk populations, the prevalence was higher, with a pooled mean of 2.7% (95% CI: 1.4–4.3%). The general population estimates incorporated two studies among Indigenous populations in Canada, which were not analyzed separately because only two studies were available.

Among men who have sex with men (MSM), pooled mean prevalence estimates were 0.9% (95% CI: 0.2–1.9%) for urogenital infection, 2.6% (95% CI: 0.9–4.9%) for anorectal infection, and 1.6% (95% CI: 0.2–3.8%) for oropharyngeal infection.

Among STI clinic attendees, the pooled mean prevalence was 7.5% (95% CI: 4.2–11.6%) for urogenital infection, 2.5% (95% CI: 0.7–5.3%) for anorectal infection, and 5.0% (95% CI: 1.8–9.6%) for oropharyngeal infection. Among individuals living with HIV and those in HIV-discordant couples, the pooled mean prevalence of urogenital infection was 0.6% (95% CI: 0.0–1.8%).

Prevalence among symptomatic populations was markedly higher—15.9% (95% CI: 4.0–33.2%) among women and 37.4% (95% CI: 11.1–68.6%) among men. In contrast, infertility clinic attendees demonstrated a very low urogenital prevalence of 0.0% (95% CI: 0.0–0.0%).

Most meta-analyses demonstrated significant heterogeneity (*p* < 0.1), with I² values generally exceeding 50%, indicating that the variability in prevalence estimates primarily reflected true differences across studies rather than sampling variation. To further explore and explain these sources of heterogeneity, meta-regression analyses were subsequently performed.

### Associations with NG prevalence and sources of between-study heterogeneity

Table 3 summarizes the results of the univariable and multivariable meta-regression analyses for urogenital NG infection, including the two multivariable models that treated calendar time as either a categorical or a continuous linear variable. The year of publication was used as the temporal variable since it was available for all studies, whereas the year of data collection required imputation for a subset (*n* = 28, 15.5%) of measures. Sensitivity analyses substituting the year of publication with the year of data collection yielded consistent results (Table 4). Collectively, the models explained most of the observed variation in prevalence across studies (adjusted R² = 53–59%).

**Table 3:** Univariable and multivariable meta-regression analyses of *Neisseria gonorrhoeae* prevalence in urogenital specimens in Canada

Urogenital speciments			Stratified prevalence measures	Sample size	Univariable analysis				Multivariable analyses				
			**Total n**	Total N	PR (95% CI)	*p*-value	LT test *p*-value	Adjusted R^2^	Model 1			Model 2	
									APR (95% CI)		*p*-value	APR (95% CI)	*p-*value
**Population characteristics**	**Population type**	General populations	53	353,423	1.00	-	< 0.001	37.01	1.00		-	1.00	-
		Intermediate-risk populations	35	12,162	3.52 (1.85–6.70)	< 0.001			5.46 (2.74–10.88)		< 0.001	4.19 (2.13–8.23)	< 0.001
		Men who have sex with men	14	5373	1.24 (0.49–3.17)	0.649			2.21 (0.89–5.52)		0.088	2.54 (1.07–6.03)	0.034
		Symptomatic women	9	901	12.59 (4.72–33.55)	< 0.001			3.56 (1.16–10.90)		0.026	4.36 (1.51–12.64)	0.007
		Symptomatic men	4	489	28.43 (8.02-100.74)	< 0.001			13.32 (3.75–47.24)		< 0.001	11.78 (3.55–39.10)	< 0.001
		Infertility clinic attendees and women with miscarriage or ectopic pregnancy^*^	6	5641	0.05 (0.01–0.39)	0.005			0.08 (0.01–0.52)		0.009	0.07 (0.01–0.42)	0.004
		STI clinic attendees	26	78,622	4.43 (2.37–8.30)	< 0.001			2.85 (1.40–5.81)		0.004	3.22 (1.64–6.35)	0.001
		Individuals living with HIV and individuals in HIV-discordant couples	5	3702	0.93 (0.18–4.85)	0.931			1.82 (0.40–8.30)		0.438	1.60 (0.37–6.85)	0.528
		Sexual contacts of persons with NG or CT infection	1	133	42.86 (3.81-482.22)	0.003			15.69 (1.63–150.70)		0.017	9.96 (1.16–85.66)	0.037
		Other populations^**†**^	28	19,644	5.28 (2.83–9.88)	< 0.001			3.22 (1.60–6.47)		0.001	2.98 (1.53–5.80)	0.002
	**Age group**	< 20 years	14	2090	1.00	-	0.764	0.00	-		-	-	-
		20–29 years	6	944	0.29 (0.03–2.53)	0.260			-		-	-	-
		30–39 years	4	3674	0.37 (0.05–2.92)	0.343			-		-	-	-
		≥ 40 years	6	1922	0.60 (0.09–4.17)	0.602			-		-	-	-
		Mixed ages	150	470,666	0.64 (0.23–1.75)	0.381			-		-	-	-
	**Sex**	Women	109	345,792	1.00	-	0.474	0.00	-		-	-	-
		Men	62	35,848	0.85 (0.48–1.49)	0.559			-		-	-	-
		Mixed sexes	10	98,369	0.53 (0.18–1.57)	0.248			-		-	-	-
	**Canadian provinces**	Alberta	22	178,446	1.00	-	0.040	4.87	1.00		-	1.00	-
		British Colombia	14	12,168	2.15 (0.73–6.33)	0.165			1.03 (0.43–2.47)		0.949	0.74 (0.31–1.73)	0.482
		Manitoba	11	5978	1.18 (0.31–4.43)	0.810			0.87 (0.29–2.58)		0.794	0.78 (0.28–2.22)	0.644
		Nova Scotia	11	44,927	1.05 (0.34–3.25)	0.934			1.35 (0.55–3.31)		0.511	1.11 (0.48–2.60)	0.801
		Ontario	56	201,311	0.43 (0.19-1.00)	0.049			1.14 (0.57–2.29)		0.718	1.01 (0.53–1.92)	0.988
		Quebec	52	28,208	1.19 (0.53–2.67)	0.674			0.59 (0.27–1.29)		0.185	0.52 (0.25–1.10)	0.086
		Saskatchewan	10	5244	1.44 (0.45–4.57)	0.536			1.19 (0.47–2.98)		0.713	1.15 (0.48–2.73)	0.757
		Unclear-mixed-other	5	3727	0.76 (0.13–4.34)	0.756			0.59 (0.15–2.41)		0.463	0.67 (0.17–2.54)	0.549
**Study methodology characteristics**	**Assay type**	NAAT/PCR	86	350,100	1.00	-	< 0.001	24.77	1.00		-	1.00	-
		Culture	88	127,733	4.68 (2.95–7.43)	< 0.001			1.60 (0.79–3.26)		0.193	0.84 (0.40–1.80)	0.660
		Gram stain and other/unclear assays^**§**^	7	2176	1.82 (0.48–6.85)	0.377			0.53 (0.15–1.90)		0.325	0.47 (0.14–1.59)	0.226
	**Sample size**	< 200	47	4925	1.00	-	0.001	8.46	1.00		-	1.00	-
		≥ 200	134	475,084	0.35 (0.19–0.65)	0.001			0.58 (0.30–1.10)		0.092	0.55 (0.30–0.99)	0.048
	**Sampling method**	Probability based	3	1534	1.00	-	0.147	1.03	1.00		-	1.00	-
		Non-probability based	178	478,475	4.00 (0.61–26.21)	0.147			1.49 (0.32–6.90)		0.610	1.10 (0.26–4.68)	0.900
	**Response rate**	≥ 80%	20	8080	1.00	-	0.034	3.12	1.00		-	1.00	-
		< 80%	1	1780	0.37 (0.02–8.57)	0.532			0.99 (0.09–10.29)		0.990	0.80 (0.09–7.44)	0.843
		Unclear	160	470,146	2.81 (1.16–6.80)	0.022			3.83 (1.74–8.43)		0.001	2.25 (1.10–4.62)	0.027
**Temporal trend**	**Year of publication category**	≤ 2005	98	43,852	1.00	-	< 0.001	27.94	1.00		-	-	-
		2006–20,015	51	411,781	0.17 (0.10–0.29)	< 0.001			0.34 (0.15–0.76)		0.009	-	-
		> 2015	32	24,376	0.25 (0.14–0.46)	< 0.001			0.37 (0.16–0.87)		0.024	-	-
	**Year of publication**		181	480,009	0.95 (0.94–0.96)	< 0.001	< 0.001	36.41	-		-	0.95 (0.93–0.97)	< 0.001

*APR* Adjusted prevalence ratio, *CI* Confidence interval, *CT* Chlamydia trachomatis, *HIV* Human immunodeficiency virus, *LT *test, Likelihood ratio test, *NAAT* Nucleic acid amplification test, *NG* Neisseria gonorrhoeae, *PCR* Polymerase chain reaction, *PR* Prevalence ratio; STI, Sexually transmitted infection

Adjusted R² for the final multivariable model = 53.49%

Adjusted R² for the final multivariable model = 58.24%

^*^ Measures from infertility clinic attendees and women with miscarriage or ectopic pregnancy were combined into one category to increase statistical power, given the small number of studies in each group and the close epidemiological relationship between these populations

^**†**^ Other populations include groups with an undetermined risk of acquiring NG infection, such as cervical cancer patients, specimens submitted to virology or bacteriology laboratories, and mixed or undefined populations

^**§**^ Measures from studies using Gram stain and those employing other or unclear assays were combined into a single category due to the limited number of studies available for each

**Table 4:** Sensitivity analyses. Univariable and multivariable meta-regression analyses of *Neisseria gonorrhoeae* prevalence in urogenital specimens in Canada, using year of data collection as the Temporal variable instead of year of publication

Urogenital speciments			Stratified prevalence measures	Sample size	Univariable analysis				Multivariable analyses				
			**Total n**	Total N	PR (95% CI)	*p*-value	LT test *p*-value	Adjusted R^2^	Model 1			Model 2	
									APR (95% CI)		*p*-value	APR (95% CI)	*p-*value
**Population characteristics**	**Population type**	General populations	53	353,423	1.00	-	< 0.001	37.01	1.00		-	1.00	-
		Intermediate-risk populations	35	12,162	3.52 (1.85–6.70)	< 0.001			5.50 (2.76–10.96)		< 0.001	4.10 (2.10–8.02)	< 0.001
		Men who have sex with men	14	5373	1.24 (0.49–3.17)	0.649			2.61 (0.98–6.91)		0.054	2.48 (1.05–5.83)	0.038
		Symptomatic women	9	901	12.59 (4.72–33.55)	< 0.001			3.70 (1.22–11.23)		0.021	4.41 (1.54–12.68)	0.006
		Symptomatic men	4	489	28.43 (8.02-100.74)	< 0.001			13.97 (3.95–49.43)		< 0.001	12.12 (3.69–39.77)	< 0.001
		Infertility clinic attendees and women with miscarriage or ectopic pregnancy^*^	6	5641	0.05 (0.01–0.39)	0.005			0.07 (0.01–0.45)		0.006	0.06 (0.01–0.36)	0.003
		STI clinic attendees	26	78,622	4.43 (2.37–8.30)	< 0.001			3.43 (1.62–7.29)		0.001	3.31 (1.69–6.48)	0.001
		Individuals living with HIV and individuals in HIV-discordant couples	5	3702	0.93 (0.18–4.85)	0.931			1.82 (0.40–8.32)		0.436	1.52 (0.36–6.47)	0.570
		Sexual contacts of persons with NG or CT infection	1	133	42.86 (3.81-482.22)	0.003			15.44 (1.62-147.18)		0.018	10.12 (1.20-85.49)	0.034
		Other populations^**†**^	28	19,644	5.28 (2.83–9.88)	< 0.001			3.39 (1.68–6.88)		0.001	2.92 (1.50–5.66)	0.002
	**Age group**	< 20 years	14	2090	1.00	-	0.763	0.00	-		-	-	-
		20–29 years	6	944	0.29 (0.03–2.53)	0.260			-		-	-	-
		30–39 years	4	3674	0.37 (0.05–2.92)	0.343			-		-	-	-
		≥ 40 years	6	1922	0.60 (0.09–4.17)	0.602			-		-	-	-
		Mixed ages	150	470,666	0.64 (0.23–1.75)	0.381			-		-	-	-
	**Sex**	Women	109	345,792	1.00	-	0.474	0.00	-		-	-	-
		Men	62	35,848	0.85 (0.48–1.49)	0.559			-		-	-	-
		Mixed sexes	10	98,369	0.53 (0.18–1.57)	0.248			-		-	-	-
	**Canadian provinces**	Alberta	22	178,446	1.00	-	0.040	4.87	1.00		-	1.00	-
		British Colombia	14	12,168	2.15 (0.73–6.33)	0.165			0.96 (0.40–2.31)		0.922	0.76 (0.33–1.76)	0.523
		Manitoba	11	5978	1.18 (0.31–4.43)	0.810			0.90 (0.30–2.68)		0.854	0.82 (0.29–2.31)	0.708
		Nova Scotia	11	44,927	1.05 (0.34–3.25)	0.934			1.27 (0.52–3.12)		0.593	0.96 (0.41–2.24)	0.917
		Ontario	56	201,311	0.43 (0.19-1.00)	0.049			1.16 (0.58–2.34)		0.673	1.02 (0.54–1.94)	0.955
		Quebec	52	28,208	1.19 (0.53–2.67)	0.674			0.62 (0.28–1.34)		0.221	0.56 (0.27–1.16)	0.118
		Saskatchewan	10	5244	1.44 (0.45–4.57)	0.536			1.19 (0.48–2.99)		0.707	1.19 (0.50–2.81)	0.693
		Unclear-mixed-other	5	3727	0.76 (0.13–4.34)	0.756			0.61 (0.15–2.46)		0.480	0.62 (0.16–2.35)	0.482
**Study methodology characteristics**	**Assay type**	NAAT/PCR	86	350,100	1.00	-	< 0.001	24.77	1.00		-	1.00	-
		Culture	88	127,733	4.68 (2.95–7.43)	< 0.001			1.45 (0.68–3.07)		0.329	0.77 (0.36–1.65)	0.492
		Gram stain and other/unclear assays^**§**^	7	2176	1.82 (0.48–6.85)	0.377			0.49 (0.14–1.75)		0.271	0.46 (0.14–1.52)	0.199
	**Sample size**	< 200	47	4925	1.00	-	0.001	8.46	1.00		-	1.00	-
		≥ 200	134	475,084	0.35 (0.19–0.65)	0.001			0.54 (0.29–1.04)		0.064	0.53 (0.29–0.95)	0.032
	**Sampling method**	Probability based	3	1534	1.00	-	0.147	1.03	1.00		-	1.00	-
		Non-probability based	178	478,475	4.00 (0.61–26.21)	0.147			1.34 (0.30–6.11)		0.700	1.40 (0.33–5.89)	0.642
	**Response rate**	≥ 80%	20	8080	1.00	-	0.034	3.12	1.00		-	1.00	-
		< 80%	1	1780	0.37 (0.02–8.57)	0.532			1.05 (0.10-10.95)		0.967	0.87 (0.09–7.96)	0.900
		Unclear	160	470,146	2.81 (1.16–6.80)	0.022			3.90 (1.75–8.71)		0.001	2.39 (1.17–4.86)	0.017
**Temporal trend**	**Year of data collection category**	< 2000	97	43,652	1.00	-	< 0.001	27.68	1.00		-	-	-
		2000–2009	39	346,239	0.16 (0.09–0.30)	< 0.001			0.38 (0.15–0.95)		0.039	-	-
		≥ 2010	45	90,118	0.24 (0.14–0.41)	< 0.001			0.29 (0.12–0.70)		0.006	-	-
	**Year of data collection**		181	480,009	0.95 (0.94–0.96)	< 0.001	< 0.001	35.71	-		-	0.95 (0.93–0.97)	< 0.001

*APR* Adjusted prevalence ratio, *CI* Confidence interval, *CT* Chlamydia trachomatis, *HIV* Human immunodeficiency virus, *LT *test Likelihood ratio test, *NAAT* Nucleic acid amplification test, *NG* Neisseria gonorrhoeae, *PCR* Polymerase chain reaction, *PR* Prevalence ratio, *STI* Sexually transmitted infection

Adjusted R² for the final multivariable model = 53.69%

Adjusted R² for the final multivariable model = 58.94%

^*^ Measures from infertility clinic attendees and women with miscarriage or ectopic pregnancy were combined into one category to increase statistical power, given the small number of studies in each group and the close epidemiological relationship between these populations

^**†**^ Other populations include groups with an undetermined risk of acquiring NG infection, such as cervical cancer patients, specimens submitted to virology or bacteriology laboratories, and mixed or undefined populations

^**§**^ Measures from studies using Gram stain and those employing other or unclear assays were combined into a single category due to the limited number of studies available for each

Population type was the strongest predictor of NG prevalence, independently explaining 37.0% of the observed variation (Table 3). Compared with the general population, prevalence was highest among sexual contacts of persons infected with NG or *Chlamydia trachomatis* and symptomatic men, followed by intermediate-risk populations, symptomatic women, STI clinic attendees, and MSM. In contrast, infertility clinic attendees and women presenting with miscarriage or ectopic pregnancy exhibited significantly lower prevalence than the general population.

No significant differences in prevalence were observed by age group, sex, or Canadian province. However, all models consistently demonstrated a downward temporal trend in NG prevalence, irrespective of whether calendar time was modeled categorically or continuously, and whether the year of data collection or year of publication was used as the temporal variable.

Study design characteristics influenced prevalence estimates. A clear small-study effect was observed, with studies enrolling ≥ 200 participants reporting approximately 45% lower prevalence than smaller studies. Studies with unclear response rates reported higher prevalence than those with high response rates (≥ 80%). However, there was no evidence of differences in prevalence by assay type or sampling method (probability-based vs. non-probability-based).

Meta-regression analyses for other anatomical sites were not conducted due to the limited number of available studies.

## Discussion

NG prevalence in Canada, estimated at around 1% in the general population, mirrors the global average of 0.8% [6, 7] and is consistent with estimates from most world regions, which are near 1% [6, 7, 21–23], including the Americas [6, 7], where Canada is situated. However, this estimate exceeds prevalence reported in population-based surveys from countries with broadly similar socioeconomic, cultural, and demographic contexts, such as the National Health and Nutrition Examination Surveys in the United States and the National Survey of Sexual Attitudes and Lifestyles in the United Kingdom, both of which report prevalence well below 1% [43, 44].

NG prevalence in Canada was found to exhibit an overall decline over the past four decades. This long-term downward trend is consistent with patterns observed in other regions [21–23]. However, the pace of decline remains insufficient to achieve WHO’s target of a 90% reduction in NG incidence by 2030 [12].

This overall downward trend may reflect the combined impact of sustained public health interventions and broader societal changes. These include the expansion of screening and testing programs [15, 17, 32], widespread adoption of NAAT/PCR diagnostics facilitating earlier detection and treatment [15, 17, 30, 32], strengthened surveillance and partner notification systems [15, 17, 45], increased public awareness of STIs [46], and the adoption of safer sexual practices in the post-HIV era [47]. However, this long-term decline may mask short-term fluctuations or recent increases in prevalence, as suggested by more recent surveillance data [15, 17].

Certain populations—such as STI clinic attendees and MSM—remain disproportionately affected, consistent with global trends [48]. Elevated NG acquisition through anal and oral sex is documented among these groups, paralleling findings from Europe [21] and South-East Asia [23]. These patterns reflect established associations between NG infection and recent high-risk sexual behaviors [2, 49–53], including multiple partnerships, inconsistent condom use, and engagement in transactional sex [2, 54, 55]. The prevalence of oropharyngeal infection is particularly concerning given its role as a reservoir for the emergence and spread of gonococcal AMR [1, 56, 57]. Collectively, these findings underscore the need to strengthen targeted prevention, screening, and treatment efforts among key populations to reduce transmission and mitigate the growing threat of AMR.

By contrast, NG prevalence among infertility clinic attendees and women with miscarriage or ectopic pregnancy was lower than that of the general population—findings consistent with other high-income settings but contrasting with the higher prevalence reported in some low- and middle-income countries [5, 21, 22]. These differences may reflect variations in healthcare access, diagnostic capacity, and screening coverage across settings [5].

NG prevalence followed a clear gradient, with higher levels observed among populations at greater sexual behavior risk, consistent with patterns reported in other regions [21–23] and for other STIs [24–27, 58]. No significant differences in prevalence were observed by sex, an unexpected finding that contrasts with evidence from other regions where men are more frequently infected than women [21–23]. As expected, prevalence was high among symptomatic men, reflecting the typically symptomatic presentation of NG infection in men [59] and underscoring its role as a leading cause of urethritis in Canada, similar to other settings [21–23]. No significant differences in prevalence were observed across age groups, consistent with findings from other regions [21–23]. This suggests that NG exposure may extend across adult populations rather than being largely confined to younger individuals, although limited statistical power in the available evidence may also have constrained detection of age-specific differences.

As anticipated, ever-infection prevalence, derived from serological measures, was substantially higher than current infection prevalence; however, its epidemiological interpretation remains limited by uncertainties surrounding the accuracy and reliability of NG serological assays [60]. A clear small-study effect [27] was observed, with studies enrolling ≥ 200 participants reporting lower prevalence than smaller studies—a well-documented phenomenon across STI prevalence research, irrespective of pathogen [21–27, 35].

This study has limitations. Data availability was uneven across anatomical sites and population groups. While urogenital NG infection was relatively well characterized, evidence on anorectal and oropharyngeal infection was limited. Notably, no studies specifically examined NG prevalence among female sex workers—a population central to STI transmission dynamics [61] and often a key focus of surveillance in other regions [21–23]. Even among MSM, the number of available studies was small, potentially limiting representativeness of the broader MSM population. Limited data from certain provinces and Indigenous populations may also have reduced the overall geographic and population representativeness of the findings.

Prevalence estimates from facility-based populations, particularly symptomatic individuals and STI clinic attendees, showed considerable variability and skewed distributions. This heterogeneity likely reflects differences in population demographics, behavioral risk profiles, healthcare-seeking patterns, and the scope and catchment of participating facilities. Evidence of publication bias was also detected, primarily linked to the small-study effect.

The meta-regression analyses assessed the influence of factors that could potentially affect observed prevalence and were extractable from the included studies. However, other potentially important factors that were not explicitly reported—such as screening coverage and population-specific screening behaviors, which likely have evolved over time with the expansion of screening and possible changes associated with increasing pre-exposure prophylaxis (PrEP) use [62]—could not be evaluated for their impact on prevalence.

The observed decline in NG prevalence over time should be interpreted with caution. The apparent steepness of this decline was partly driven by high prevalence estimates reported in early studies from previous decades, which may not have been representative of the true population-level burden at the time. Moreover, analyses of overall temporal trends may obscure fluctuations in prevalence or periods of increase or decrease, including potential recent increases associated with expanding PrEP use [62]. Nevertheless, the multiple analyses conducted in this study consistently support a long-term decline in NG prevalence, although the possibility of more recent increases cannot be excluded.

Marked heterogeneity in prevalence across studies was evident, reflecting differences in population composition, study period, diagnostic assays, sampling methods, sample sizes, and response rates. Over time, diagnostic practices shifted toward more sensitive NAAT-based assays; however, most studies continued to rely on convenience rather than probability-based sampling. Studies with unclear response rates tended to report higher prevalence, and a clear small-study effect was observed. Furthermore, confirmatory testing following reactive molecular results was not routinely performed; given the potentially suboptimal positive predictive value of unconfirmed reactive tests for some assays in low-prevalence populations [63], prevalence estimates may have been inflated. Collectively, these findings suggest that studies with weaker methodological rigor were more likely to overestimate NG prevalence, whereas those employing more robust designs produced lower and more reliable estimates.

These methodological limitations may have contributed to an overestimation of the pooled mean prevalence and underscore the need for future research using standardized study designs, probability-based sampling, consistent diagnostic approaches, and transparent reporting. Strengthening methodological rigor is essential to improve the accuracy, reliability, and comparability of STI epidemiological estimates in Canada and globally. There is also a particular need for expanded and targeted research among specific populations, especially FSWs, MSM, and Indigenous populations, to address existing evidence gaps and strengthen surveillance.

This study has strengths. It employed a comprehensive and systematic search strategy across multiple international databases, without restrictions on language or publication year, ensuring extensive coverage of the available evidence. Rigorous assessments of study quality and risk of bias were conducted, including analytical evaluations of how methodological factors influenced reported prevalence estimates. By consolidating a large and diverse body of prevalence data, the study generated a robust synthesis of evidence that allowed detailed examination of epidemiological patterns and sources of heterogeneity. Importantly, the meta-regression analyses explained more than half of the observed variation in prevalence, underscoring the combined impact of epidemiological and methodological factors.

In conclusion, this systematic review provided a comprehensive overview of NG epidemiology in Canada over more than four decades. NG prevalence in the general population remains similar to global levels but is markedly higher among key populations, including MSM, symptomatic individuals, and STI clinic attendees. Although prevalence has declined, the reduction is insufficient to meet WHO’s 2030 target. The persistence of infection and the rise of gonococcal AMR highlight the need for sustained investment in targeted prevention, enhanced surveillance—including extragenital screening—and standardized, high-quality data to inform policy and prepare for future NG vaccine deployment.

## Supplementary Information

Supplementary Material 1.

## Data Availability

All data generated or analyzed during this study are included in this published article and its Supplementary Material file.

## Declarations

### Use of artificial intelligence tools

ChatGPT was used solely for grammar verification and phrasing refinement. The authors thoroughly reviewed and edited the text, taking full responsibility for its accuracy and quality.

## Abbreviations

AMR: Antimicrobial resistance
CI: Confidence interval
HIV: Human immunodeficiency virus
LFK: Luis Furuya-Kanamori index
MSM: Men who have sex with men
NAAT: Nucleic acid amplification test
NG: Neisseria gonorrhoeae
PCR: Polymerase chain reaction
PR: Prevalence ratio
PRISMA: Preferred Reporting Items for Systematic Reviews and Meta-Analyses
STI: Sexually transmitted infection
WHO: World Health Organization
