# Supplementary material for: Prevalence and epidemiological patterns of Neisseria gonorrhoeae infection in Canada, 1969–2025: a systematic review and meta-analysis
Source: BMC Public Health. 2026 Mar 3;26:1148. doi: 10.1186/s12889-026-26579-y (PMC13064092; doi:10.1186/s12889-026-26579-y)
Supplement: Supplementary file 1 — Supplementary Material 1. [file 12889_2026_26579_MOESM1_ESM.docx]

**Supplementary Material**

Table of Contents

[**Table S1.** Preferred Reporting Items for Systematic Reviews and Meta-analyses (PRISMA) checklist [1]. 3](#_Toc215579375)

[**Table S2.** Data sources and search strategies used to identify studies reporting *Neisseria gonorrhoeae* prevalence in Canada. 5](#_Toc215579376)

[**Box S1.** Variables extracted from relevant records meeting the inclusion criteria. 6](#_Toc215579377)

[**Table S3.** Range of quality assessment components relevant to prevalence studies and their applicability to the methods of this systematic review and the included studies reporting *Neisseria gonorrhoeae* prevalence in Canada. 7](#_Toc215579378)

[**Box S2.** Factors (variables) selected *a priori* and included in the univariable and multivariable meta-regression analyses. 8](#_Toc215579379)

[**Table S4.** List of publications meeting the inclusion criteria of this systematic review, from which *Neisseria gonorrhoeae* prevalence measures were extracted. 9](#_Toc215579380)

[**Table S5.** Summary of precision assessment and risk of bias assessment for studies reporting *Neisseria gonorrhoeae* prevalence in Canada. 14](#_Toc215579381)

[**Table S6.** Assessment of publication bias for studies reporting *Neisseria gonorrhoeae* prevalence in Canada using Doi plots and the LFK index [4]. 15](#_Toc215579382)

[**Figure S1.** Doi plots assessing publication bias among studies reporting urogenital *Neisseria gonorrhoeae* prevalence in Canada. 16](#_Toc215579383)

[**Figure S2.** Doi plots assessing publication bias among studies reporting anorectal *Neisseria gonorrhoeae* prevalence in Canada. 17](#_Toc215579384)

[**Figure S3.** Doi plots assessing publication bias among studies reporting oropharyngeal *Neisseria gonorrhoeae* prevalence in Canada. 18](#_Toc215579385)

[**Figure S4.** Doi plot assessing publication bias among studies reporting serological (ever-infection) *Neisseria gonorrhoeae* prevalence in Canada. 19](#_Toc215579386)

[**Figure S5.** Doi plots assessing publication bias among studies reporting *Neisseria gonorrhoeae* prevalence in unspecified/mixed specimens Canada. 20](#_Toc215579387)

[**Table S7.** Pooled mean prevalence of *Neisseria gonorrhoeae* in Canada, stratified by anatomical site, population type, assay type. 21](#_Toc215579388)

[**Figure S6.** Forest plots presenting outcomes of the pooled mean *Neisseria gonorrhoeae* prevalence in urogenital specimens among different populations in Canada. 24](#_Toc215579389)

[**Figure S7.** Forest plots presenting outcomes of the pooled mean *Neisseria gonorrhoeae* prevalence in anorectal specimens among different populations in Canada. 33](#_Toc215579390)

[**Figure S8.** Forest plots presenting outcomes of the pooled mean *Neisseria gonorrhoeae* prevalence in oropharyngeal specimens among different populations in Canada. 35](#_Toc215579391)

[**Figure S9.** Forest plots presenting outcomes of the pooled mean *Neisseria gonorrhoeae* prevalence in serological specimens among general populations in Canada. 37](#_Toc215579392)

[**Figure S10.** Forest plots presenting outcomes of the pooled mean *Neisseria gonorrhoeae* prevalence in unspecified/mixed specimens among different populations in Canada. 38](#_Toc215579393)

[**References** 43](#_Toc215579394)

# **Table S1.** Preferred Reporting Items for Systematic Reviews and Meta-analyses (PRISMA) checklist [1].

| **Section and topic** | **Item #** | **Checklist item** | **Location where item is reported** |
| --- | --- | --- | --- |
| **Title** | | |  |
| Title | 1 | Identify the report as a systematic review. | Title |
| **Abstract** | | |  |
| Abstract | 2 | See the PRISMA 2020 for Abstracts checklist (table 2). | Abstract section |
| **Introduction** | | |  |
| Rationale | 3 | Describe the rationale for the review in the context of existing knowledge. | Background section |
| Objectives | 4 | Provide an explicit statement of the objective(s) or question(s) the review addresses. | Background section |
| **Methods** | | |  |
| Eligibility criteria | 5 | Specify the inclusion and exclusion criteria for the review and how studies were grouped for the syntheses. | Methods: Study selection and inclusion and exclusion criteria section |
| Information sources | 6 | Specify all databases, registers, websites, organisations, reference lists and other sources searched or consulted to identify studies. Specify the date when each source was last searched or consulted. | Methods: Data sources and search strategy section; Table S2 |
| Search strategy | 7 | Present the full search strategies for all databases, registers and websites, including any filters and limits used. | Table S2 |
| Selection process | 8 | Specify the methods used to decide whether a study met the inclusion criteria of the review, including how many reviewers screened each record and each report retrieved, whether they worked independently, and if applicable, details of automation tools used in the process. | Methods: Study selection process and inclusion and exclusion criteria section |
| Data collection process | 9 | Specify the methods used to collect data from reports, including how many reviewers collected data from each report, whether they worked independently, any processes for obtaining or confirming data from study investigators, and if applicable, details of automation tools used in the process. | Methods: Data extraction and synthesis section |
| Data items | 10a | List and define all outcomes for which data were sought. Specify whether all results that were compatible with each outcome domain in each study were sought (e.g., for all measures, time points, analyses), and if not, the methods used to decide which results to collect. | Methods: Data extraction and synthesis section; Boxes S1 and S2 |
|  | 10b | List and define all other variables for which data were sought (e.g., participant and intervention characteristics, funding sources). Describe any assumptions made about any missing or unclear information. | Boxes S1 and S2 |
| Study risk of bias assessment | 11 | Specify the methods used to assess risk of bias in the included studies, including details of the tool(s) used, how many reviewers assessed each study and whether they worked independently, and if applicable, details of automation tools used in the process. | Methods: Precision, risk of bias, and publication bias section |
| Effect measures | 12 | Specify for each outcome the effect measure(s) (e.g. risk ratio, mean difference) used in the synthesis or presentation of results. | Methods: Data extraction and synthesis section; Boxes S1 and S2 |
| Synthesis methods | 13a | Describe the processes used to decide which studies were eligible for each synthesis (e.g. tabulating the study intervention characteristics and comparing against the planned groups for each synthesis (item #5)). | Methods: Meta-analyses and Meta-regressions sections; Boxes S1 and S2 |
|  | 13b | Describe any methods required to prepare the data for presentation or synthesis, such as handling of missing summary statistics, or data conversions. | Methods: Meta-analyses and Meta-regressions sections; Boxes S1 and S2 |
|  | 13c | Describe any methods used to tabulate or visually display results of individual studies and syntheses. | Methods: Meta-analyses and Meta-regressions sections; Boxes S1 and S2 |
|  | 13d | Describe any methods used to synthesise results and provide a rationale for the choice(s). If meta-analysis was performed, describe the model(s), method(s) to identify the presence and extent of statistical heterogeneity, and software package(s) used. | Methods: Meta-analyses and Meta-regressions sections; Boxes S1 and S2 |
|  | 13e | Describe any methods used to explore possible causes of heterogeneity among study results (e.g. subgroup analysis, metaregression). | Methods: Meta-analyses and Meta-regressions sections; Boxes S1 and S2 |
|  | 13f | Describe any sensitivity analyses conducted to assess robustness of the synthesised results. | Methods: Meta-analyses and Meta-regressions sections; Boxes S1 and S2 |
| Reporting bias assessment | 14 | Describe any methods used to assess risk of bias due to missing results in a synthesis (arising from reporting biases). | N/A |
| Certainty assessment | 15 | Describe any methods used to assess certainty (or confidence) in the body of evidence for an outcome. | N/A |
| **Results** | | |  |
| Study selection | 16a | Describe the results of the search and selection process, from the number of records identified in the search to the number of studies included in the review, ideally using a flow diagram (see fig 1). | Results: Search results, scope of evidence, and prevalence overview section; Figure 1 |
|  | 16b | Cite studies that might appear to meet the inclusion criteria, but which were excluded, and explain why they were excluded. | Figure 1 |
| Study characteristics | 17 | Cite each included study and present its characteristics. | Results: Search results, scope of evidence, and prevalence overview section; Table 2 and Tables S4 and S7 |
| Risk of bias in studies | 18 | Present assessments of risk of bias for each included study. | Results: Precision, risk of bias, and publication bias assessments; Tables S5 and S6; Figures S1-S5 |
| Results of individual studies | 19 | For all outcomes, present, for each study: (a) summary statistics for each group (where appropriate) and (b) an effect estimate and its precision (e.g. confidence/credible interval), ideally using structured tables or plots. | Table 2; Table S7; Figures S6-S10 |
| Results of syntheses | 20a | For each synthesis, briefly summarise the characteristics and risk of bias among contributing studies. | Results: Precision, risk of bias, and publication bias assessments; Tables S5 and S6; Figures S1-S5 |
|  | 20b | Present results of all statistical syntheses conducted. If meta-analysis was done, present for each the summary estimate and its precision (e.g. confidence/credible interval) and measures of statistical heterogeneity. If comparing groups, describe the direction of the effect. | Results: Pooled mean estimates of gonorrhea prevalence section; Table 2; Table S7; Figures S6-S10 |
|  | 20c | Present results of all investigations of possible causes of heterogeneity among study results. | Results Predictors of prevalence and sources of between-study heterogeneity section; Tables 3 and 4 |
|  | 20d | Present results of all sensitivity analyses conducted to assess the robustness of the synthesised results. | Predictors of prevalence and sources of between-study heterogeneity section; Tables 3 and 4 |
| Reporting biases | 21 | Present assessments of risk of bias due to missing results (arising from reporting biases) for each synthesis assessed. | N/A |
| Certainty of evidence | 22 | Present assessments of certainty (or confidence) in the body of evidence for each outcome assessed. | N/A |
| **Discussion** | | |  |
| Discussion | 23a | Provide a general interpretation of the results in the context of other evidence. | Discussion section |
|  | 23b | Discuss any limitations of the evidence included in the review. | Discussion section |
|  | 23c | Discuss any limitations of the review processes used. | Discussion section |
|  | 23d | Discuss implications of the results for practice, policy, and future research. | Discussion section |
| **Other information** | | |  |
| Registration and  protocol | 24a | Provide registration information for the review, including register name and registration number, or state that the review was not registered. | N/A |
|  | 24b | Indicate where the review protocol can be accessed, or state that a protocol was not prepared. | N/A |
|  | 24c | Describe and explain any amendments to information provided at registration or in the protocol. | N/A |
| Support | 25 | Describe sources of financial or non-financial support for the review, and the role of the funders or sponsors in the review. | Funding and Acknowledgements |
| Competing interests | 26 | Declare any competing interests of review authors. | Declaration of interests |
| Availability of data, code, and other materials | 27 | Report which of the following are publicly available and where they can be found: template data collection forms; data extracted from included studies; data used for all analyses; analytic code; any other materials used in the review. | Data sharing statement |

Abbreviations: NA, Not applicable.

# **Table S2.** Data sources and search strategies used to identify studies reporting *Neisseria gonorrhoeae* prevalence in Canada.

| **PubMed (Last searched: August 7, 2025)** |
| --- |
| ("Neisseria gonorrhoeae"[Mesh] OR "Gonorrhea"[Mesh] OR "Pelvic Inflammatory Disease"[Mesh] OR "Epididymitis"[Mesh] OR "Orchitis"[Mesh] OR "Neisseria gonorrhoeae"[Text] OR "Gonorrhoeae"[Text] OR "Gonorrhea"[Text] OR "Gonococcus"[Text] OR "Gonococci"[Text] OR "Gonococcal"[Text] OR "Gonococcal infection"[Text] OR "Pelvic inflammatory disease"[Text] OR "Gonococcal epididymitis"[Text] OR "Orchi-epididymitis"[Text] OR "Orchiepididymitis"[Text] OR "seminal vesicle disease"[Text]) AND (Canada[MeSH] OR Canad*[Text] OR Alberta[Text] OR British Columbia[Text] OR Manitoba[Text] OR New Brunswick[Text] OR Newfoundland and Labrador[Text] OR Northwest Territories[Text] OR Nova Scotia[Text] OR Nunavut[Text] OR Ontario[Text] OR Prince Edward Island[Text] OR Quebec[Text] OR Saskatchewan[Text] OR Yukon Territory[Text]) |
| **Embase (Last searched: August 7, 2025)** |
| (exp gonorrhea / or exp neisseria gonorrhoeae / or exp epididymitis / or exp orchitis / or exp pelvic inflammatory disease/ or gonorrhea.mp. or neisseria gonorrhoeae.mp. or gonorrhoeae.mp. or gonococcus.mp. or gonococci.mp. or gonococcal.mp. or gonococcal infection.mp. or pelvic inflammatory disease.mp. or gonococcal epididymitis.mp. or orchi-epididymitis.mp. or orchiepididymitis.mp. or seminal vesicle disease.mp. or seminal disease.mp. or seminal vasculitis.mp.) AND (exp Canada/ OR Canad*.mp. OR Alberta.mp. OR British Columbia.mp. OR Manitoba.mp. OR New Brunswick.mp. OR "Newfoundland and Labrador".mp. OR Northwest Territories.mp. OR Nova Scotia.mp. OR Nunavut.mp. OR Ontario.mp. OR Prince Edward Island.mp. OR Quebec.mp. OR Saskatchewan.mp. OR Yukon.mp. ) |
| **Web of Science (Last searched: August 7, 2025)** |
| ((((ALL=(gonorrhoeae)) OR ALL=(gonorrhea)) OR ALL=(Neisseria gonorrhoeae )) OR ALL=(gonococcus)) OR ALL=(gonococcal) and Canada |
| **SCOPUS (Last searched: August 7, 2025)** |
| ALL ( gonorrhea ) OR ALL ( neisseria AND gonorrhoeae ) OR ALL ( gonorrhoeae ) OR ALL ( gonococcus ) OR ALL ( gonococcal ) AND ( LIMIT-TO ( AFFILCOUNTRY , "Canada" ) ) |

#

# **Box S1.** Variables extracted from relevant records meeting the inclusion criteria.

- Author(s)
- Year of publication
- Full citation
- Country
- City
- Study design
- Sampling methodology
- Year(s) of data collection
- Study site
- Study population
- Population characteristics (e.g., sex and age)
- Response rate
- Sample size of tested population
- Number of participants positive for *Neisseria gonorrhoeae* infection
- Reported *Neisseria gonorrhoeae* prevalence
- Anatomical site: urogenital (urethral, vaginal, endocervical, urine, and semen), anorectal, oropharyngeal, serum, and unclear
- Type of assay used for infection ascertainment: nucleic acid amplification test/polymerase chain reaction, culture, gram stain, blood tested for antibodies, and unclear

# **Table S3.** Range of quality assessment components relevant to prevalence studies and their applicability to the methods of this systematic review and the included studies reporting *Neisseria gonorrhoeae* prevalence in Canada.

| **Risk of bias tool from Hoy, 2012 [2]** | **Risk of bias tool from Munn, 2015 [3]** | **Risk of bias assessment of included studies** |
| --- | --- | --- |
| Was the study’s target population a close representation of the national population in relation to relevant variables? | Were study participants sampled in an appropriate way? | Met in the study design of this systematic review. The systematic review investigated prevalence in all population groups. The meta-regression analyses explored the impact of population type on observed prevalence. The sampling method was one of the investigated risk of bias domains. |
| Was the sampling frame a true or close representation of the target population? | Was the sample frame appropriate to address the target population? | Met in the study design of this systematic review. Included as the probability-based versus non-probability-based sampling risk of bias domain. The meta-regression analyses also explored the impact of sampling method on observed prevalence. |
| Was some form of random selection used to select the sample, OR was a census undertaken? |  | Met in the study design of this systematic review. Included as the probability-based versus non-probability-based sampling risk of bias domain. The meta-regression analyses also explored the impact of sampling method on observed prevalence. |
| Was the likelihood of nonresponse bias minimal? | Was the response rate adequate, and if not, was the low response rate managed appropriately? | Met in the study design of this systematic review. Included as the response rate risk of bias domain. The meta-regression analyses also explored the impact of response rate on observed prevalence. |
| Were data collected directly from the subjects (as opposed to a proxy)? |  | Met in the study design of this systematic review. The inclusion criteria specified that only studies based on biomarkers collected directly from individuals are included in this systematic review. |
| Was an acceptable case definition used in the study? | Were valid methods used for the identification of the condition? | Met in the study design of this systematic review. A standardized and consistent case definition was used, that of NG infection diagnosed through laboratory methods. |
| Was the study instrument that measured the parameter of interest shown to have validity and reliability? | Was the condition measured in a standard, reliable way for all participants? | Met in the study design of this systematic review. The laboratory methods utilized in the included studies are generally well-established, with acceptable levels of specificity and sensitivity and are widely employed in both research and clinical settings. Importantly, the meta-regression analyses examined the effect of assay type on the observed prevalence |
| Was the same mode of data collection used for all subjects? |  | Met in the study design of this systematic review. It is standard for STI studies involving biomarkers, by design, to use a consistent mode of data collection from all subjects recruited for a study, including specimen type and assay type. |
| Was the length of the shortest prevalence period for the parameter of interest appropriate? |  | Met in the study design of this systematic review. Included studies reported point prevalence measures, that is, prevalence based on a cross-sectional survey at a specific and appropriately relevant time interval. |
| Were the numerator(s) and denominator(s) for the parameter of interest appropriate? | Was there appropriate statistical analysis? | Met in the study design of this systematic review. The numerator and denominator were defined with no ambiguity: number of positive NG cases over total number of tested subjects. |
|  | Was the data analysis conducted with sufficient coverage of the identified sample? | Met in the study design of this systematic review. It is standard practice in such STI studies for data analysis to be conducted on the full reported and tested sample. |
|  | Was the sample size adequate? | Met in the study design of this systematic review. Included as the precision assessment of the sample size. Importantly, the meta-regression analyses explored the impact of sample size on observed prevalence. |
|  | Were the study subjects and the setting described in detail? | Met in the study design of this systematic review. For all included studies, the population type of study subjects and the study site were available and extracted. Populations were classified according to this systematic review definitions of study populations. |

Abbreviations: NG, *Neisseria gonorrhoeae*; STI, Sexually transmitted infection.

#

# **Box S2.** Factors (variables) selected *a priori* and included in the univariable and multivariable meta-regression analyses.

| 1. Population type as defined in Table 1 2. Sex 3. Age groups classified to best fit reported data as:  - <20 years old - 20-29 years old - 30-39 years old - ≥40 years old - Mixed ages  1. Assay type:  - NAAT/PCR - Culture - Gram stain - Other  1. Sample size:  - <200 - ≥200  1. Sampling method:  - Probability-based sampling^*^ - Non-probability-based sampling**^†^**  1. Response rate:  - ≥80% - <80% - Unclear  1. Year of publication category:  - <2005 - 2005-2014 - ≥2015  1. Year of publication as a continuous linear term 2. Year of data collection category**^§^**  - <2000 - 2000-2009 - ≥2010  1. Year of data collection as a linear term |
| --- |

Abbreviations: NAAT, Nucleic acid amplification test; PCR, Polymerase chain reaction.

‎^*^ Probability-based sampling, as reported in the reviewed publications, includes random sampling and respondent-driven sampling.‎

‎**^†^** Non-probability sampling, as reported in the reviewed publications, includes convenience sampling and snowball sampling.‎

**^§^** The categories were defined based on the observed median interval of 3.5 years between the year of data collection and the year of publication, which was rounded to 5 years to create a standardized 5-year bracket.

# **Table S4.** List of publications meeting the inclusion criteria of this systematic review, from which *Neisseria gonorrhoeae* prevalence measures were extracted.

| 1. Ablona A, Grennan T, Hart T, Shoveller J, Cox J, Ogilvie G, Krajden M, Fairley C, Haag D, Gilbert M: **The impact of including throat and rectal swabs for chlamydia and gonorrhea testing online in British Columbia, Canada**. *Sexually Transmitted Infections* 2019, **95(Supplement 1)**:A277.  2. Aggarwal A, Spitzer RF, Caccia N, Stephens D, Johnstone J, Allen L: **Repeat Screening for Sexually Transmitted Infection in Adolescent Obstetric Patients**. *Journal of Obstetrics and Gynaecology Canada* 2010, **32(10)**:956-961.  3. Al-Bargash D, Shahinc R, Stuart R: **The recent emergence of lymphogranuloma venereum reports among men in toronto: A descriptive study of cases reported between january 1 2014 and december 30 2017**. *Sexually Transmitted Diseases* 2018, **45(Supplement 2)**:S96.  4. Allard R, Robert J, Turgeon P, Lepage Y: **Predictors of asymptomatic gonorrhea among patients seen by private practitioners**. *Cmaj* 1985, **133**(11):1135-1139, 1146.  5. Anand CM, Gubash SM: **Evaluation of the GO Slide (Roche) growth transport system for isolation of Neisseria gonorrhoeae from clinical specimens**. *Journal of Clinical Microbiology* 1986, **24**(1):96-98.  6. Arias M, Jang D, Gilchrist J, Luinstra K, Li J, Smieja M, Chernesky MA: **Ease, Comfort, and Performance of the HerSwab Vaginal Self-Sampling Device for the Detection of Chlamydia trachomatis and Neisseria gonorrhoeae**. *Sexually Transmitted Diseases* 2016, **43**(2):125-129.  7. Atwood CV, Fang L, Demers A, Gratrix J, Rossi M, Taylor D, Wright J, Wong T, Jayaraman G: **Co-infection with sexually transmitted infections among Canadian street-involved youth 2001-2006**. *Sexually Transmitted Infections* 2011, **1)**:A29-A30.  8. Bazin S, Bouchard C, Brisson J, Morin C, Meisels A, Fortier M: **Vulvar vestibulitis syndrome: An exploratory case-control study**. *Obstet Gynecol* 1994, **83**(1):47-50.  9. Bowie WR, Borrie-Hume CJ, Manzon LM, Fawcett A, Percival-Smith RK, Jones HD: **Prevalence of Chlamydia trachomatis and Neisseria gonorrhoeae in two different populations of women**. *Can Med Assoc J* 1981a, **124**(11):1477-1479.  10. Bowie WR, Jones H: **Acute pelvic inflammatory disease in outpatients: association with Chlamydia trachomatis and Neisseria gonorrhoeae**. *Ann Intern Med* 1981b, **95**(6):685-688.  11. Bowie WR, Willetts V, Binns BA, Brunham RC: **Etiology of cervicitis and treatment with minocycline**. *Can J Infect Dis* 1993, **4**(2):95-100.  12. Brodeur BR, Ashton FE, Diena BB: **Enzyme-linked immunosorbent assay with polyvalent gonococcal antigen**. *J Med Microbiol* 1982, **15**(1):1-9.  13. Brunham RC, Binns B, Guijon F, Danforth D, Kosseim ML, Rand F, McDowell J, Rayner E: **Etiology and outcome of acute pelvic inflammatory disease**. *J Infect Dis* 1988, **158**(3):510-517.  14. Brunham RC, Peeling R, Maclean I, Kosseim ML, Paraskevas M: **Chlamydia trachomatis-associated ectopic pregnancy: Serologic and histologic correlates**. *J Infect Dis* 1992, **165**(6):1076-1081.  15. Burchell AN, Grewal R, Allen VG, Gardner SL, Moravan V, Bayoumi AM, Kaul R, McGee F, Millson ME, Remis RS *et al*: **Modest rise in chlamydia and gonorrhoea testing did not increase case detection in a clinical HIV Cohort in Ontario, Canada**. *Sexually Transmitted Infections* 2014, **90(8)**:608-614.  16. Bush KR, Henderson EA, Dunn J, Read RR, Singh A: **Mapping the core: Chlamydia and gonorrhea infections in Calgary, Alberta**. *Sexually Transmitted Diseases* 2008, **35(3)**:291-297.  17. Caffaro Rouget A, Mah JK, Lang RA, Joffres MR: **Prevalence of sexually transmitted diseases in juvenile prostitutes and street youth**. *Canadian Journal of Infectious Diseases* 1994, **5(1)**:21-27.  18. Caloenescu M, Larose G, Birry A, Roy J, Kasatiya SS: **Genital infection in juvenile delinquent females**. *Br J Vener Dis* 1973, **49**(1):72-77.  19. Candler E, Naeem Khan M, Gratrix J, Plitt S, Stadnyk M, Smyczek P, Anderson N, Carter J, Sayers S, Smith D *et al*: **Retrospective audit of a convenience cohort of individuals on HIV pre-exposure prophylaxis in Alberta, Canada**. *J Assoc Med Microbiol Infect Dis Can* 2022, **7**(4):350-363.  20. Chan EL, Brandt K, Olienus K, Antonishyn N, Horsman GB: **Performance characteristics of the Becton Dickinson ProbeTec system for direct detection of Chlamydia trachomatis and Neisseria gonorrhoeae in male and female urine specimens in comparison with Roche Cobas systems**. *Archives of Pathology and Laboratory Medicine* 2000, **124(11)**:1649-1652.  21. Chernesky M: **Comparison of a new aptima specimen collection and transportation kit to l-pap for detection of c trachomatis, n gonorrhoeae and T vaginalis in cervical and vaginal specimens**. *SEXUALLY TRANSMITTED INFECTIONS* 2011, **87**:A73-A74.  22. Chernesky M, Freund GG, Hook Iii E, Leone P, D'Ascoli P, Martens M: **Detection of Chlamydia trachomatis and Neisseria gonorrhoeae infections in north American women by testing SurePath liquid-based pap specimens in APTIMA assays**. *Journal of Clinical Microbiology* 2007, **45**(8):2434-2438.  23. Chernesky M, Jang D, Aries M, Smieja M, Ratman S: **Self-obtained vaginal swabs detected more chlamydia trachomatis, neisseria gonorrhoeae and mycoplasma genitalium infections than first catch urine collected at home compared to a clinic**. *Sexually Transmitted Diseases* 2018, **45(Supplement 2)**:S16.  24. Chernesky M, Jang D, Gilchrist J, Hatchette T, Poirier A, Flandin JF, Smieja M, Ratnam S: **Head-to-head comparison of second-generation nucleic acid amplification tests for detection of Chlamydia trachomatis and Neisseria gonorrhoeae on urine samples from female subjects and self-collected vaginal swabs**. *Journal of Clinical Microbiology* 2014, **52**(7):2305-2310.  25. Chernesky M, Jang D, Portillo E, Chong S, Smieja M, Luinstra K, Petrich A, MacRitchie C, Ewert R, Hayhoe B *et al*: **Abilities of APTIMA, AMPLICOR, and ProbeTec assays to detect Chlamydia trachomatis and Neisseria gonorrhoeae in PreservCyt ThinPrep liquid-based Pap samples**. *Journal of Clinical Microbiology* 2007, **45**(8):2355-2358.  26. Chernesky M, Jang D, Portillo E, Smieja M, Kapala J, Doucette C, Sumner J, Ewert R, MacRitchie C, Gilchrist J: **Comparison of three assays for detection of Chlamydia trachomatis and Neisseria gonorrhoeae in surepath pap samples and the role of pre- and postcytology testing**. *Journal of Clinical Microbiology* 2012, **50(4)**:1281-1284.  27. Chernesky M, Jang D, Smieja M, Arias M, Martin I, Weinbaum B, Getman D: **Urinary Meatal Swabbing Detects More Men Infected with Mycoplasma genitalium and Four Other Sexually Transmitted Infections Than First Catch Urine**. *Sexually Transmitted Diseases* 2017, **44**(8):489-491.  28. Chernesky M, Jang D, Smieja M, Portillo E, Kapala J, Sumner J: **Burden of infection with <i>C</i>. <i>trachomatis</i>, <i>N</i>. <i>gonorrhoeae</i>, <i>T-vaginalis</i> and HR-HPV in homeless youth determined by APTIMA testing**. *INTERNATIONAL JOURNAL OF INFECTIOUS DISEASES* 2010, **14**:E353-E353.  29. Chernesky MA, Jang D, Portillo E, Smieja M, Gilchrist J, Ewert R, MacRitchie C: **Self-collected swabs of the urinary meatus diagnose more Chlamydia trachomatis and Neisseria gonorrhoeae infections than first catch urine from men**. *Sexually Transmitted Infections* 2013, **89**(2):102-104.  30. Chernesky MA, Martin DH, Hook EW, Willis D, Jordan J, Wang S, Lane JR, Fuller D, Schachter J: **Ability of new APTIMA CT and APTIMA GC assays to detect Chlamydia trachomatis and Neisseria gonorrhoeae in male urine and urethral swabs**. *Journal of Clinical Microbiology* 2005, **43**(1):127-131.  31. Church DL, Amante L, Semeniuk H, Gregson DB: **Selective testing of women based on age for genital Chlamydia trachomatis and Neisseria gonorrhoeae infection in a centralized regional microbiology laboratory**. *Diagnostic Microbiology and Infectious Disease* 2007, **57(4)**:379-385.  32. D'Aiuto C, Valderrama A, Byrns M, Boucoiran I: **Sexually Transmitted and Blood-Borne Infections in Pregnant Women and Adverse Pregnancy Outcomes**. *Journal of Obstetrics and Gynaecology Canada* 2020, **42(8)**:977-983.  33. Diemert DJ, Libman MD, Lebel P: **Confirmation by 16S rRNA PCR of the COBAS AMPLICOR CT/NG test for diagnosis of <i>Neisseria gonorrhoeae</i> infection in a low-prevalence population**. *JOURNAL OF CLINICAL MICROBIOLOGY* 2002, **40**(11):4056-4059.  34. Domes T, Lo KC, Grober ED, Mullen JB, Mazzulli T, Jarvi K: **The utility and cost of Chlamydia trachomatis and Neisseria gonorrhoeae screening of a male infertility population**. *Fertil Steril* 2012, **97**(2):299-305.  35. Drews S, Chui L, Douglas L, Zahariadis G, Tyrrell G: **Confirmation of the Roche COBAS AMPLICOR 480 Neisseria gonorrhea molecular assay with an in-house real-time polymerase chain reaction assay**. *Canadian Journal of Infectious Diseases and Medical Microbiology* 2011, **SA)**:11A-12A.  36. Embil JA, Garner JB, Pereira LH, White FMM, Manuel FR: **Association of cytomegalovirus and herpes simplex virus infections of the cervix in four clinic populations**. *Sexually Transmitted Diseases* 1985, **12**(4):224-228.  37. Embree JE, Lindsay D, Williams T, Peeling RW, Wood S, Morris M: **Acceptability and usefulness of vaginal washes in premenarcheal girls as a diagnostic procedure for sexually transmitted diseases**. *PEDIATR INFECT DIS J* 1996, **15**(8):662-667.  38. Flores Anato JL, Panagiotoglou D, Greenwald ZR, Blanchette M, Trottier C, Vaziri M, Charest L, Szabo J, Thomas R, Maheu-Giroux M: **Chemsex and incidence of sexually transmitted infections among Canadian pre-exposure prophylaxis (PrEP) users in the l'Actuel PrEP Cohort (2013-2020)**. *Sexually Transmitted Infections* 2022, **(no pagination)**.  39. Forward KR: **Risk of coinfection with Chlamydia trachomatis and Neisseria gonorrhoeae in Nova Scotia**. *Canadian Journal of Infectious Diseases and Medical Microbiology* 2010, **21(2)**:e84-e86.  40. Friedland SN, Slapcoff B, Dylewski J: **Presumptive Treatment of Chlamydia and Gonorrhea Infections in a Canadian Ambulatory Emergency Department Setting: Determination of Overtreatment and Undertreatment Rates**. *Infectious Diseases in Clinical Practice* 2017, **25(6)**:320-322.  41. Gander S, Scholten V, Osswald I, Sutton M, van Wylick R: **Cervical Dysplasia and Associated Risk Factors in a Juvenile Detainee Population**. *Journal of Pediatric and Adolescent Gynecology* 2009, **22(6)**:351-355.  42. Gardhouse CE, Levett PN, Horsman GB: **Epidemiology, co-infection and laboratory testing of chlamydia trachomatis and neisseria gonorrhoeae in Saskatchewan**. *Canadian Journal of Infectious Diseases and Medical Microbiology* 2012, **SB)**:19B.  43. Gesink D, Sarai Racey C, Seah C, Zittermann S, Mitterni L, Juzkiw J, Jamieson H, Greer J, Singh S, Jensen JS *et al*: **Mycoplasma genitalium in Toronto, Ont: Estimates of prevalence and macrolide resistance**. *Canadian Family Physician* 2016, **62(2)**:e96-e101.  44. Gilbert M, Hottes TS, Haag D, Bondyra M, Thomson K, Holgerson N, Grennan T, Kopp S, Fairley C, Krajden M *et al*: **Use of a comprehensive internet-based STI/HIV testing service in vancouver, British Columbia and uptake by promotion strategy**. *Sexually Transmitted Diseases* 2016, **43(10 Supplement 2)**:S147.  45. Gohil N, Mushanski L, Wanlin T, Lepe A, Lang A, Minion J, Dillon J: **Mycoplasma genitalium: the most prevalent STI in Saskatchewan, canada, has a high prevalence of resistance to macrolides and fluoroquinolones**. *Sexually Transmitted Infections* 2021, **97(SUPPL 1)**:A116-A117.  46. Grant LJ: **Assessment of child sexual abuse: Eighteen months' experience at the Child Protection Center**. *American Journal of Obstetrics and Gynecology* 1984, **148**(5):617-620.  47. Gratrix J, Bergman J, Brandley J, Parker P, Smyczek P, Singh AE: **Impact of introducing triage criteria for express testing at a canadian sexually transmitted infection clinic**. *Sexually Transmitted Diseases* 2015, **42(11)**:660-663.  48. Gratrix J, Plitt S, Turnbull L, Smyczek P, Brandley J, Scarrott R, Naidu P, Bertholet L, Chernesky M, Read R *et al*: **Trichomonas vaginalis Prevalence and Correlates in Women and Men Attending STI Clinics in Western Canada**. *Sexually Transmitted Diseases* 2017, **44(10)**:627-629.  49. Gratrix J, Singh AE, Bergman J, Egan C, McGinnis J, Drews SJ, Read R: **Prevalence and characteristics of rectal chlamydia and gonorrhea cases among men who have sex with men after the introduction of nucleic acid amplification test screening at 2 Canadian sexually transmitted infection clinics**. *Sexually Transmitted Diseases* 2014, **41(10)**:589-591.  50. Gratrix J, Singh AE, Bergman J, Egan C, Plitt SS, McGinnis J, Bell CA, Drews SJ, Read R: **Evidence for increased chlamydia case finding after the introduction of rectal screening among women attending 2 Canadian sexually transmitted infection clinics**. *Clinical Infectious Diseases* 2015, **60(3)**:398-404.  51. Gratrix J, Smyczek P, Bertholet L, Lee MC, Pyne D, Woods D, Courtney K, Ahmed R: **A cross-sectional evaluation of opt-in testing for sexually transmitted and blood-borne infections in three Canadian provincial correctional facilities: a missed opportunity for public health?** *International journal of prisoner health* 2019, **15(3)**:273-281.  52. Grewal R, Allen VG, Gardner SL, Bayoumi AM, Kaul R, Mazzulli T, McGee F, Raboud J, Rourke SB, Tan DH *et al*: **Risk factors for chlamydia and gonorrhea diagnosis among men who have sex with men in HIV care in Ontario**. *Canadian Journal of Infectious Diseases and Medical Microbiology* 2015, **SB)**:80B.  53. Guijon F, Paraskevas M, Rand F, Heywood E, Brunham R, McNicol P: **Vaginal microbial flora as a cofactor in the pathogenesis of uterine cervical intraepithelial neoplasia**. *Int J Gynaecol Obstet* 1992, **37**(3):185-191.  54. Guijon FB, Paraskevas M, Brunham R: **The association of sexually transmitted diseases with cervical intraepithelial neoplasia: A case-control study**. *American Journal of Obstetrics and Gynecology* 1985, **151**(2):185-190.  55. Haley N, Lambert G, Jean S, Frappier JY, Otis J, Roy E: **Mental health distress and alcohol misuse associated with prevalence of sexually transmitted infections among adolescents in care**. *European Psychiatry Conference: 21st European Congress of Psychiatry, EPA* 2013, **28**(SUPPL. 1).  56. Haley N, Roy E, Leclerc P, Lambert G, Boivin JF, Cedras L, Vincelette J: **Risk behaviours and prevalence of Chlamydia trachomatis and Neisseria gonorrhoeae genital infections among Montreal street youth**. *International Journal of STD and AIDS* 2002, **13(4)**:238-245.  57. Hall CW, Pyke A, Davydov M, Urbanoski K, Guimond TH, Woodward K: **Impact of first-void urine volume on chlamydia and gonorrhea positivity rates in men who have sex with men and transgender women**. *Microbiol Spectr* 2025:e0307224.  58. Hart TA, Moore DM, Noor SW, Lachowsky N, Grace D, Cox J, Skakoon-Sparling S, Jollimore J, Parlette A, Lal A: **Prevalence of HIV and sexually transmitted and blood-borne infections, and related preventive and risk behaviours, among gay, bisexual and other men who have sex with men in Montreal, Toronto and Vancouver: results from the Engage Study**. *Canadian journal of public health* 2021, **112**(6):1020-1029.  59. Harvey-Lavoie S, Apelian H, Labbe AC, Cox J, Messier-Peet M, Moodie EEM, Fourmigue A, Moore D, Lachowsky NJ, Grace D *et al*: **Community-Based Prevalence Estimates of Chlamydia trachomatis and Neisseria gonorrhoeae Infections among Gay, Bisexual, and Other Men Who Have Sex with Men in Montreal, Canada**. *Sexually Transmitted Diseases* 2021, **48(12)**:939-944.  60. Hennink M, Abbas Z, Choudhri Y, Diener T, Lloyd K, Archibald CP, Cule S: **Risk behaviours for infection with HIV and hepatitis C virus among people who inject drugs in Regina, Saskatchewan**. *Canada communicable disease report = Releve des maladies transmissibles au Canada* 2007, **33(5)**:53-59.  61. Hill LH, Ruparelia H, Embil JA: **Nonspecific vaginitis and other genital infections in three clinic populations**. *Sex Transm Dis* 1983, **10**(3):114-118.  62. Hill LV, Luther ER, Young D, Pereira L, Embil JA: **Prevalence of lower genital tract infections in pregnancy**. *Sexually Transmitted Diseases* 1988, **15**(1):5-10.  63. Hovhannisyan G, Lee C, Hogg-Johnson S, Bondy S, Millson M: **Risk factors and prevalence of chlamydia and gonorrhoea in public health sexual health clinics in Hamilton, ON**. *Sexually Transmitted Infections Conference: STI and AIDS World Congress* 2013, **89**(SUPPL. 1).  64. Hughes EG, Mowatt J, Spence JE: **Endocervical Chlamydia trachomatis infection in Canadian adolescents**. *Cmaj* 1989, **140**(3):297-301.  65. Ivensky V, Mandel R, Boulay AC, Lavallée C, Benoît J, Labbé AC: **Suboptimal prenatal screening of Chlamydia trachomatis and Neisseria gonorrhoeae infections in a Montréal birthing and tertiary care centre: A retrospective cohort study**. *Can Commun Dis Rep* 2021, **47**(4):209-215.  66. Jiang H, Lukac C, Ogilvie G, Gilbert M, Grennan T, Wong J: **Core groups of individuals with chlamydia and/or gonorrhoea reinfections have increased odds of diagnosis with infectious syphilis: A population-based retrospective cohort study In British Columbia, Canada, 2006-2015**. *Sexually Transmitted Infections* 2017, **93(Supplement 2)**:A30-A31.  67. Kapala J, Biers K, Cox M, Kamionka M, Sumner J, Toor R, Gilchrist J, Jang D, Smieja M, Chernesky M: **Aptima combo 2 testing detected additional cases of Neisseria gonorrhoeae infection in men and women in community settings**. *Journal of Clinical Microbiology* 2011, **49**(5):1970-1971.  68. Kendall P, Whynot E, Gomber E: **A retrospective study of 191 cases of pelvic inflammatory disease**. *Can J Public Health* 1977, **68**(4):318-322.  69. Kouyoumdjian FG, Main C, Calzavara LM, Kiefer L: **Prevalence and predictors of urethral chlamydia and gonorrhea infection in male inmates in an Ontario correctional facility**. *Canadian Journal of Public Health* 2011, **102(3)**:220-224.  70. Krahn J, Gratrix J, Khan M, Meyer G, Smyczek P, Singh AE: **Retrospective Cohort Study of Financial Incentives for Sexually Transmitted Infection Testing and Treatment in an Outreach Population in Edmonton, Canada, 2018-2019**. *Sex Transm Dis* 2025, **52**(1):37-42.  71. Lambert G, Haley N, Jean S, Tremblay C, Frappier JY, Otis J, Roy E: **Sexual health of adolescents in Quebec residential youth protection centres**. *Canadian Journal of Public Health* 2013, **104(3)**:e216-e221.  72. Landis SJ, Stewart IO, Chernesky MA, Mahony JB, Cunningham AI, Grenier-Landis MN, Seidelman WE: **Value of the gram-stained urethral smear in the management of men with urethritis**. *Sexually Transmitted Diseases* 1988, **15**(2):78-84.  73. Leblanc J, Roberts C, Delong T, Macdonald J, Heinstein C, Hatchette T: **Detection of neisseria gonorrhoea using viper XTR; confirmation is necessary to avoid false positive results**. *Canadian Journal of Infectious Diseases and Medical Microbiology* 2012, **23**(SUPPL. SB):8B.  74. Leclerc P, Gallant S, Morissette C, Roy E: **Rates of sexually transmitted and blood-borne infections and related risk behaviours among street youth in Montreal**. *Canadian Journal of Infectious Diseases and Medical Microbiology* 2013, **SA)**:82A.  75. Levallois P, Rioux J-E, Cote L: **Chlamydial infection among females attending an abortion clinic: prevalence and risk factors**. *CMAJ: Canadian Medical Association Journal* 1987, **137**(1):33.  76. Levallois P, Rioux JE: **Prophylactic antibiotics for suction curettage abortion: results of a clinical controlled trial**. *Am J Obstet Gynecol* 1988, **158**(1):100-105.  77. Levett PN, Brandt K, Olenius K, Brown C, Montgomery K, Horsman GB: **Evaluation of three automated nucleic acid amplification systems for detection of Chlamydia trachomatis and Neisseria gonorrhoeae in first-void urine specimens**. *Journal of Clinical Microbiology* 2008, **46(6)**:2109-2111.  78. Li J, Jang D, Gilchrist J, Smieja M, Ewert R, MacRitchie C, Chernesky M: **Comparison of flocked and Aptima swabs and two specimen transport media in the Aptima combo 2 assay**. *Journal of Clinical Microbiology* 2014, **52**(10):3808-3809.  79. Lindegger M, Salway Hottes T, Gilbert M, Lester R, Imperial M: **Epidemiology of lymphogranuloma venereum (LGV) in BC, 2004-2012: Increased transmission or better detection of endemic disease?** *Canadian Journal of Infectious Diseases and Medical Microbiology* 2012, **SB)**:39B-40B.  80. Machouf N, Thomas R, O'Brien R, Vezina S, Longpre D, Legault D, Milne M, Fafard J, Trottier B: **Risk factors for STIS among MSM attending a sexually transmitted disease clinic in Montreal, Canada**. *Sexually Transmitted Infections* 2011, **1)**:A144.  81. Mann TA, Uddin Z, Hendriks AM, Bouchard CJ, Etches VG: **Get tested why not? a novel approach to internet-based Chlamydia and gonorrhea testing in Canada**. *Canadian Journal of Public Health* 2013, **104(3)**:e205-e209.  82. Massé R, Laperriere H, Rousseau H, Lefebvre J, Remis R: **Chlamydia trachomatis cervical infection: prevalence and determinants among women presenting for routine gynecologic examination**. *CMAJ: Canadian Medical Association Journal* 1991, **145**(8):953.  83. Mitchell-Foster S, Racey CS, Day T, Falkner C, Smith L, Pedersen H, Chan T, Cook D, Shannon K, Lee M *et al*: **Opportunities for HPV self-collection to improve cervical cancer screening uptake in street entrenched women in rural regional centres**. *American Journal of Obstetrics and Gynecology* 2019, **221(6)**:695.  84. Mushanski LM, Brandt K, Coffin N, Levett PN, Horsman GB, Rank EL: **Comparison of the BD Viper System with XTR Technology to the Gen-Probe APTIMA COMBO 2 Assay using the TIGRIS DTS system for the detection of Chlamydia trachomatis and Neisseria gonorrhoeae in urine specimens**. *Sex Transm Dis* 2012, **39**(7):514-517.  85. Nelson LE, Tharao W, Husbands W, Sa T, Zhang N, Kushwaha S, Absalom D, Kaul R: **The epidemiology of HIV and other sexually transmitted infections in African, Caribbean and Black men in Toronto, Canada**. *BMC Infect Dis* 2019, **19**(1):294.  86. Nguyen VK, Greenwald ZR, Trottier H, Cadieux M, Goyette A, Beauchemin M, Charest L, Longpre D, Lavoie S, Tossa HG *et al*: **Incidence of sexually transmitted infections before and after preexposure prophylaxis for HIV**. *Aids* 2018, **32(4)**:523-530.  87. Niruban J, Meyer G, Parker P, Gratrix J, Smyczek P: **Incentive testing and treatment for STBBI in hard to reach populations in Edmonton, Alberta, Canada**. *Sexually Transmitted Infections* 2019, **95(Supplement 1)**:A171.  88. O'Byrne P, Dias R: **Urine drop-off testing: A self-directed method for STI screening and prevention**. *Canadian Journal of Human Sexuality* 2008, **17(1-2)**:53-59.  89. O'Byrne P, MacPherson P, Ember A, Grayson MO, Bourgault A: **Overview of a gay men's STI/HIV testing clinic in Ottawa: clinical operations and outcomes**. *Canadian journal of public health = Revue canadienne de sante publique* 2014, **105(5)**:e389-e394.  90. O'Byrne P, Orser L: **Overfilled urine specimens for gonorrhea and chlamydia testing: Implications for practice**. *Appl Nurs Res* 2018, **39**:121-124.  91. O'Byrne P, Orser L, Vandyk A: **Immediate PrEP after PEP: Results from an Observational Nurse-Led PEP2PrEP Study**. *Journal of the International Association of Providers of AIDS Care* 2020, **19**(no pagination).  92. Orser L, Tran V, O'Byrne P, Kroch A, Bonnetsmueller M, Hasso M, Musten A: **Testing for extragenital Neisseria gonorrhoeae and Chlamydia trachomatis: At-home pharyngeal and rectal self-swabs are non-inferior to those completed in healthcare settings**. *PLoS One* 2024, **19**(5):e0302785.  93. Ota KV, Tamari IE, Smieja M, Jamieson F, Jones KE, Towns L, Juzkiw J, Richardson SE: **Detection of Neisseria gonorrhoeae and Chlamydia trachomatis in pharyngeal and rectal specimens using the BD Probetec ET system, the Gen-Probe Aptima Combo 2 assay and culture**. *Sexually Transmitted Infections* 2009.  94. Otis J, Blais M, Veillette-Bourbeau L, Rodrigue C, Haig T, Wainberg MA, Rousseau R: **Social drivers of STI and HIV infection among participants at SPOT, a community-based testing intervention for MSM in Montreal**. *Canadian Journal of Infectious Diseases and Medical Microbiology* 2014, **SA)**:95A.  95. Parmar NR, Mushanski L, Wanlin T, Lepe A, Lang A, Minion J, Dillon JR: **High Prevalence of Macrolide and Fluoroquinolone Resistance-Mediating Mutations in Mycoplasma genitalium-Positive Urine Specimens From Saskatchewan**. *Sex Transm Dis* 2021, **48**(9):680-684.  96. Patrick DM, Rekart ML, Knowles L: **Unsatisfactory performance of the leukocyte esterase test of first voided urine for rapid diagnosis of urethritis**. *GENITOURIN MED* 1994, **70**(3):187-190.  97. Pereira LH, Embil JA, Haase DA, Manley KM: **Prevalence of human immunodeficiency virus in the patient population of a sexually transmitted disease clinic. Association with syphilis and gonorrhea**. *Sexually Transmitted Diseases* 1992, **19(2)**:115-120.  98. Pilkie D, Gratrix J, Sawatzky P, Martin I, Singh A, Prasad E, Naidu P, Mulvey M, Wong T, Smyczek PA: **Molecular Surveillance and Prediction of Antimicrobial Resistance of Neisseria gonorrhoeae in Northern Alberta, Canada, 2015 to 2018**. *Sexually Transmitted Diseases* 2022, **49(5)**:377-382.  99. Portnoy J, Mendelson J, Clecner B, Heisler L: **Asymptomatic gonorrhea in the male**. *Can Med Assoc J* 1974, **110**(2):169 passim.  100. Poulin C, Alary M, Bernier F, Carbonneau D, Boily MC, Joly JR: **Prevalence of chlamydia trachomatis and neisseria gonorrhoeae among at-risk women, young sex workers, and street youth attending community organizations in Quebec city, Canada**. *Sexually Transmitted Diseases* 2001, **28(8)**:437-443.  101. Poulin C, Alary M, Bernier F, Ringuet J, Joly JR: **Prevalence of Chlamydia trachomatis, Neisseria gonorrhoeae, and HIV infection among drug users attending an STD/HIV prevention and needle- exchange program in Quebec City, Canada**. *Sexually Transmitted Diseases* 1999, **26(7)**:410-420.  102. Raval M, Gratrix J, Plitt S, Niruban J, Smyczek P, Dong K, Singh AE: **Retrospective Cohort Study Examining the Correlates of Reported Lifetime Stimulant Use in Persons Diagnosed with Infectious Syphilis in Alberta, Canada, 2018 to 2019**. *Sexually Transmitted Diseases* 2022, **49(8)**:551-559.  103. Redditt VJ, Janakiram P, Graziano D, Rashid M: **Health status of newly arrived refugees in Toronto, Ont: Part 1: Infectious diseases**. *Canadian Family Physician* 2015, **61(7)**:e303-e309 and e331-e337.  104. Reekie A, Gratrix J, Smyczek P, Woods D, Poshtar K, Courtney K, Ahmed R: **A Cross-Sectional, Retrospective Evaluation of Opt-Out Sexually Transmitted Infection Screening at Admission in a Short-Term Correctional Facility in Alberta, Canada**. *Journal of correctional health care : the official journal of the National Commission on Correctional Health Care* 2022, **28(6)**:429-438.  105. Regimbal-Ethier M, Benomar K, To V, Quesnel M: **Prelib: Evaluating a newly launched Canadian provider of innovative internet-based services for self-directed HIV and STI screening**. *HIV Medicine* 2019, **20**(Supplement 9):195.  106. Remis RS, Liu J, Loutfy M, Tharao W, Rebbapragada A, Perusini SJ, Chieza L, Saunders M, Green-Walker L, Kaul R: **The epidemiology of sexually transmitted co-infections in HIV-positive and HIV-negative African-Caribbean women in Toronto**. *BMC Infectious Diseases* 2013, **13(1) (no pagination)**.  107. Remis RS, Liu J, Loutfy MR, Tharao W, Rebbapragada A, Huibner S, Kesler M, Halpenny R, Grennan T, Brunetta J *et al*: **Prevalence of sexually transmitted viral and bacterial infections in HIV-positive and hivnegative men who have sex with men in Toronto**. *PLoS ONE* 2016, **11(7) (no pagination)**.  108. Rodas CU, Ronald AR: **Comparison of three serological tests in gonococcal infection**. *Appl Microbiol* 1974, **27**(4):695-698.  109. Romanowski B, Dempster M, Forsey E: **Evaluation of the Isocult® diagnostic culturing system**. *Canadian Journal of Public Health* 1986, **77**(1):37-40.  110. Rouget AC, Lang RA, Joffres MR: **Sexually transmitted diseases in abused children and adolescents**. *Annals of Sex Research* 1988, **1**(1):95-114.  111. Rousseau S, Morisset ME, Knoefel F, Gattereau D, Morisset R: **The role of laparoscopy in acute pelvic infections**. *Eur J Obstet Gynecol Reprod Biol* 1991, **40**(1):49-55.  112. Rusch MLA, Shoveller JA, Burgess S, Stancer K, Patrick DM, Tyndall MW: **Demographics, sexual risk behaviours and uptake of screening for sexually transmitted infections among attendees of a weekly women-only community clinic program**. *Canadian Journal of Public Health* 2008, **99(4)**:257-261.  113. Salway T, Butt ZA, Wong S, Abdia Y, Balshaw R, Rich AJ, Ablona A, Wong J, Grennan T, Yu A *et al*: **A Computable Phenotype Model for Classification of Men Who Have Sex With Men Within a Large Linked Database of Laboratory, Surveillance, and Administrative Healthcare Records**. *Front Digit Health* 2020, **2**:547324.  114. Schleihauf E, Leonard E, Phillips C, Hatchette T, Haldane D, Arnason T, Martin I, Whelan N: **Increase in Gonorrhea Incidence Associated with Enhanced Partner Notification Strategy**. *Sexually Transmitted Diseases* 2019, **46(11)**:706-712.  115. Sellors JW, Mahony JB, Pickard L, Jang D, Groves D, Luinstra KE, Chernesky MA: **Screening urine with a leukocyte esterase strip and subsequent chlamydial testing of asymptomatic men attending primary care practitioners**. *Sexually Transmitted Diseases* 1993, **20**(3):152-157.  116. Sellors JW, Pickard L, Gafni A, Goldsmith CH, Jang D, Mahony JB, Chernesky MA: **Effectiveness and efficiency of selective vs universal screening for chlamydial infection in sexually active young women**. *Arch Intern Med* 1992, **152**(9):1837-1844.  117. Shtibel R: **Evaluation of the charcoal transport and 'Transgrow' media as holding media for N. gonorrhoeae**. *Canadian Journal of Public Health* 1975, **66(2)**:128-129.  118. Singh A, Taylor M, Krasowski A, Turner K, McDermott L, Ahmad R, Conroy P, Guenette T, Plitt S: **Women in the Shadows (WIS): Impact of a peer-based outreach programme in reaching street-involved pregnant women during a heterosexual syphilis outbreak in Alberta, Canada**. *International Journal of Antimicrobial Agents* 2009, **34**(Suppl. 2):S107.  119. Sivachandran N, Siemieniuk RAC, Murphy P, Sharp A, Walach C, Placido T, Bogoch II: **Sexually transmitted infections and viral hepatitides in patients presenting for non-occupational HIV post-exposure prophylaxis: Results of a prospective cohort study**. *International Journal of Infectious Diseases* 2015, **40**:142-144.  120. Steenbeek A, Tyndall M, Sheps S, Rothenberg R: **An epidemiological survey of chlamydial and gonococcal infections in a canadian arctic community**. *Sexually Transmitted Diseases* 2009, **36(2)**:79-83.  121. Talbot H, Romanowski B: **Factors affecting urine EIA sensitivity in the detection of Chlamydia trachomatis in men**. *GENITOURIN MED* 1994, **70**(2):101-104.  122. Tenbergen M, Dryer J: **Pelvic inflammatory disease: Diagnosis and treatment in a tertiary emergency department**. *Canadian Journal of Emergency Medicine* 2015, **17(Supplement 2)**:S84.  123. Thibeault R, Escobar Careaga R, Lavallee C, Labbe A, Roy G, Fortin C: **Screening rates and follow-up of chlamydia trachomatis and neisseria gonorrhoeae infections during pregnancy**. *Sexually Transmitted Infections* 2021, **97**(SUPPL 1):A168.  124. Thomas E, Scott SD, Grefkees I, Hession G, Pollock R, Martin T, Albritton W: **Validity and cost-effectiveness of the Gonozyme test in the diagnosis of gonorrhea**. *Cmaj* 1986, **134**(2):121-124, 146.  125. Toshach S: **Effects of meningococcal carriage on serological tests for gonorrhoea**. *Canadian Journal of Public Health* 1978, **69(2)**:127-129.  126. Toshach S, Coull I, Sigurdson S, Dublenko S, Grocholski J, Linarez L: **Evaluation of five serologic tests for antibody to neisseria gonorrhoeae**. *Sexually Transmitted Diseases* 1979, **6**(3):214-217.  127. Tyker A, Pudwell J, Schneiderman M, Hundal P, Thorne J, Jamieson MA: **Prevalence of Chlamydia and Gonorrhea Among Pregnant Adolescents Screened in the Third Trimester Using a Urine PCR Test: A Retrospective Review**. *Journal of Obstetrics and Gynaecology Canada* 2021, **43(9)**:1069-1075.  128. Vainder M, Kives S, Yudin MH: **Screening for Gonorrhea and Chlamydia in Pregnancy: Room for Improvement**. *Journal of Obstetrics and Gynaecology Canada* 2019, **41(9)**:1289-1294.  129. Vincelette J, Baril JG, Allard R: **Predictors of chlamydial infection and gonorrhea among patients seen by private practitioners**. *Cmaj* 1991, **144(6)**:713-721.  130. Waters JR, Roulston TM: **Gonococcal infection in a prenatal clinic**. *Am J Obstet Gynecol* 1969, **103**(4):532-536.  131. Wenman WM, Tataryn IV, Joffres MR, Pearson R, Grace MGA, Albritton WL, Prasad E, Boyd JJ, Chua RCM, Iwaniuk G *et al*: **Demographic, clinical and microbiological characteristics of maternity patients: A Canadian clinical cohort study**. *Canadian Journal of Infectious Diseases* 2002, **13(5)**:311-318.  132. Wong JMH, Av-Gay G, Lee T, Azampanah A, Elwood C, van Schalkwyk J, Sauvé L, Money D: **Sexually transmitted infections and bacterial vaginosis and preterm birth in pregnant people living with HIV: A population-based cohort study**. *International Journal of STD and AIDS* 2025.  133. Woolnough KV, Domovitch E, Wilson D: **SCREENING FEMALES FOR GONORRHEA**. *CANADIAN FAMILY PHYSICIAN* 1981, **27**(MAY):849-+.  134. Wylie JL, Jolly A: **Patterns of chlamydia and gonorrhea infection in sexual networks in Manitoba, Canada**. *Sex Transm Dis* 2001, **28**(1):14-24.  135. Young TK, McNicol P, Beauvais J: **Factors associated with human papillomavirus infection detected by polymerase chain reaction among urban Canadian Aboriginal and non-Aboriginal women**. *Sexually Transmitted Diseases* 1997, **24(5)**:293-298.  136. Zur R, Casson M, Bellaire J, Yudin M: **Unintended Consequences: The Impact of Cervical Cancer Screening Guidelines on Rates of STI Screening in Primary Care**. *Journal of Obstetrics and Gynaecology Canada* 2021, **43(3)**:344-351. |
| --- |

# **Table S5.** Summary of precision assessment and risk of bias assessment for studies reporting *Neisseria gonorrhoeae* prevalence in Canada.

| **Quality assessment** | ***Neisseria gonorrhea* prevalence measures** | |
| --- | --- | --- |
|  | **Number of studies** | **%** |
| **Precision of prevalence measures**^*^ | | |
| Low precision | 72 | 31.9 |
| High precision | 154 | 68.1 |
| **Risk of bias quality domain^†^** | | |
| **Sampling method** | | |
| Low risk of bias | 7 | 3.1 |
| High risk of bias | 219 | 96.9 |
| **Response rate** | | |
| Low risk of bias | 24 | 10.6 |
| High risk of bias | 1 | 0.4 |
| Unclear risk of bias | 201 | 88.9 |
| **Summary of the risk of bias assessment** | | |
| **Low risk of bias** |  |  |
| In at least one quality domain | 31 | 13.7 |
| In both quality domains | 0 | 0.0 |
| **High risk of bias** |  |  |
| In at least one quality domain | 219 | 96.9 |
| In both quality domains | 1 | 0.4 |
| **Unclear risk of bias** |  |  |
| In at least one quality domain | 201 | 88.9 |
| In both quality domains | 0 | 0.0 |
| **Prevalence studies where risk of bias assessment was possible** | **226** | **100** |

^*^Precision was assessed based on the overall sample size (not each stratum subsample size) of the study as reported in the record/publication.

**^†^**Risk of bias was assessed based on the overall sample size (not each stratum subsample size) of the study as reported in the record/publication.

# **Table S6.** Assessment of publication bias for studies reporting *Neisseria gonorrhoeae* prevalence in Canada using Doi plots and the LFK index [4].

| **Population type** | **Number of measures** | **LFK index** | **Doi plot inspection** | **Interpretation** |
| --- | --- | --- | --- | --- |
| **Current urogenital infection** | | | | |
| General populations | 52 | 5.89 | Asymmetrical Doi plot | Indicative of potential publication bias |
| Intermediate-risk populations | 35 | 0.67 | Symmetrical Doi plot | No publication bias |
| Men who have sex with men | 14 | 1.94 | Asymmetrical Doi plot | Indicative of potential publication bias |
| Symptomatic women | 9 | -0.96 | Symmetrical Doi plot | No publication bias |
| Symptomatic men | 4 | 0.96 | Symmetrical Doi plot | No publication bias |
| Infertility clinic attendees | 5 | 5.61 | Asymmetrical Doi plot | Indicative of potential publication bias |
| STI clinic attendees | 26 | 4.66 | Asymmetrical Doi plot | Indicative of potential publication bias |
| Individuals living with HIV and individuals in HIV-discordant couples | 5 | -5.67 | Asymmetrical Doi plot | Indicative of potential publication bias |
| Other populations^*^ | 29 | 3.98 | Asymmetrical Doi plot | Indicative of potential publication bias |
| **Current anorectal infection** | | | | |
| Men who have sex with men | 13 | 1.78 | Asymmetrical Doi plot | Indicative of potential publication bias |
| STI clinic attendees | 5 | -0.72 | Symmetrical Doi plot | No publication bias |
| **Current oropharyngeal infection** | | | | |
| Men who have sex with men | 12 | 1.95 | Asymmetrical Doi plot | Indicative of potential publication bias |
| STI clinic attendees | 3 | 5.72 | Asymmetrical Doi plot | Indicative of potential publication bias |
| **Serology (ever infection)** |  |  |  |  |
| General populations | 5 | 4.98 | Asymmetrical Doi plot | Indicative of potential publication bias |
| **Current infection in unspecified/mixed specimens** |  |  |  |  |
| General populations | 79 | -3.19 | Asymmetrical Doi plot | Indicative of potential publication bias |
| Intermediate-risk populations | 4 | 5.61 | Asymmetrical Doi plot | Indicative of potential publication bias |
| Men who have sex with men | 7 | -3.26 | Asymmetrical Doi plot | Indicative of potential publication bias |
| STI clinic attendees | 9 | -4.28 | Asymmetrical Doi plot | Indicative of potential publication bias |
| Patients with confirmed or suspected STIs and related infections | 6 | 7.07 | Asymmetrical Doi plot | Indicative of potential publication bias |

Abbreviations: HIV, Human immunodeficiency virus; LFK, Luis Furuya-Kanamori; STI, Sexually transmitted infection.

A minimum of three studies was required to perform this assessment.

^*^Other populations include groups with an undetermined risk of acquiring NG infection, such as cervical cancer patients, specimens submitted to virology or bacteriology laboratories, and mixed or undefined populations.

# **Figure S1.** Doi plots assessing publication bias among studies reporting urogenital *Neisseria gonorrhoeae* prevalence in Canada.

| - 1. General populations | - 1. Intermediate-risk populations | - 1. Men who have sex with men |
| --- | --- | --- |
| 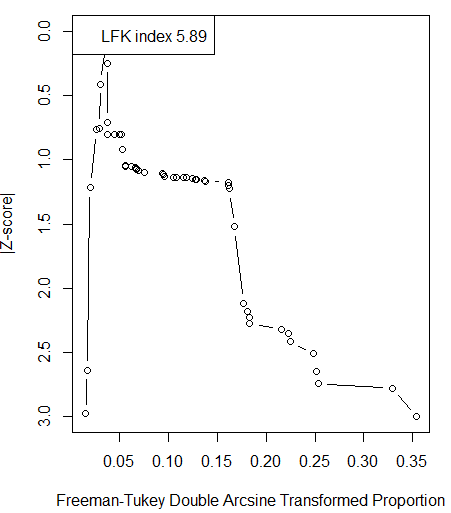 | 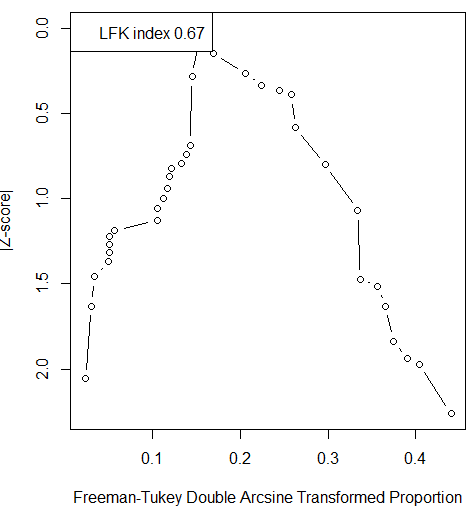 | 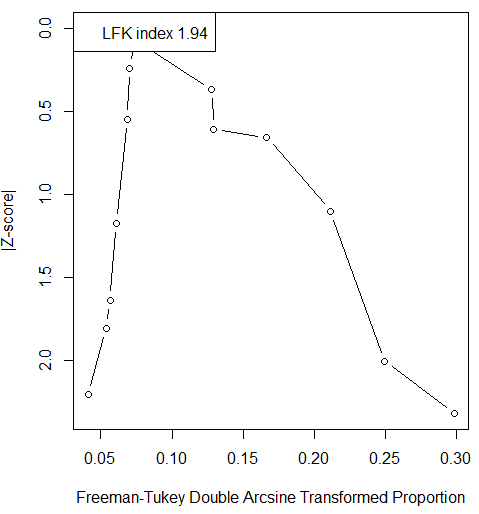 |
| - 1. Symptomatic women | - 1. Symptomatic men | - 1. Infertility clinic attendees |
| 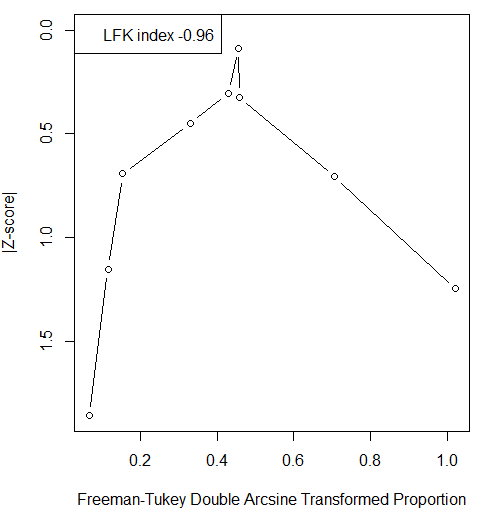 | 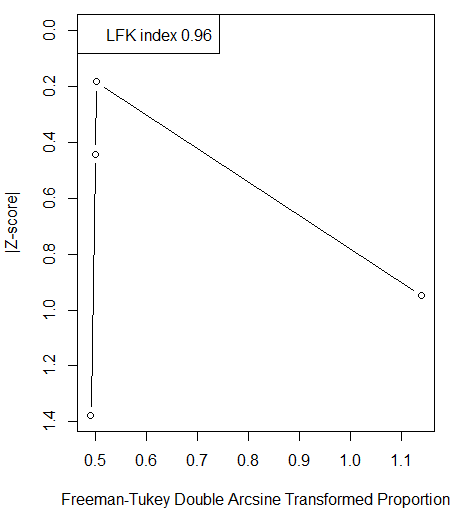 | 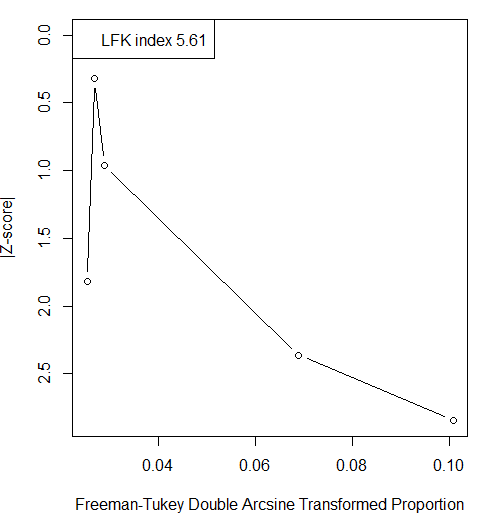 |
| - 1. STI clinic attendees | - 1. Individuals living with HIV and individuals in HIV-discordant couples | - 1. Other Populations^*^ |
| 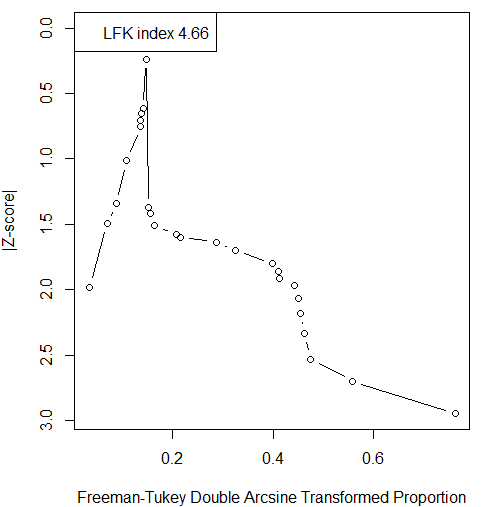 | 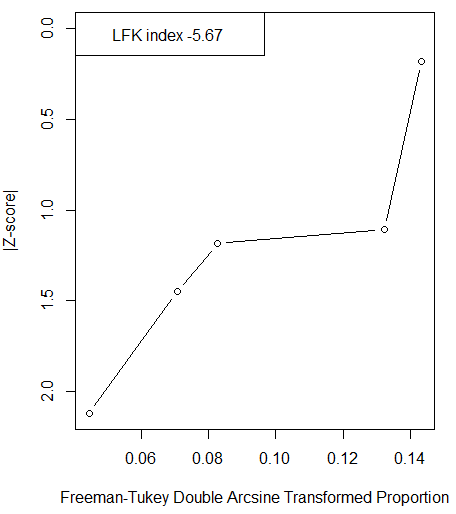 | 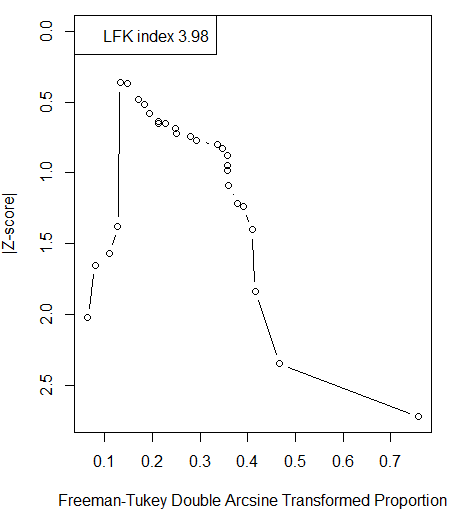 |

Abbreviations: HIV, Human immunodeficiency virus; LFK, Luis Furuya-Kanamori; STI, Sexually transmitted infection.

A minimum of three studies was required to perform this assessment.

^*^ Other populations include groups with an undetermined risk of acquiring NG infection, such as cervical cancer patients, specimens submitted to virology or bacteriology laboratories, and mixed or undefined populations.

# **Figure S2.** Doi plots assessing publication bias among studies reporting anorectal *Neisseria gonorrhoeae* prevalence in Canada.

| 1. Men who have sex with men | 1. STI clinic attendees |
| --- | --- |
| 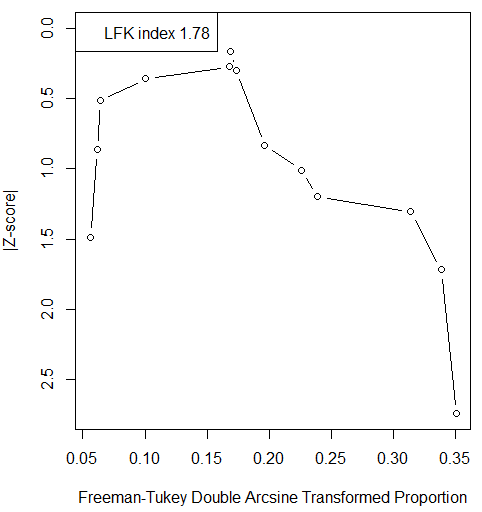 | 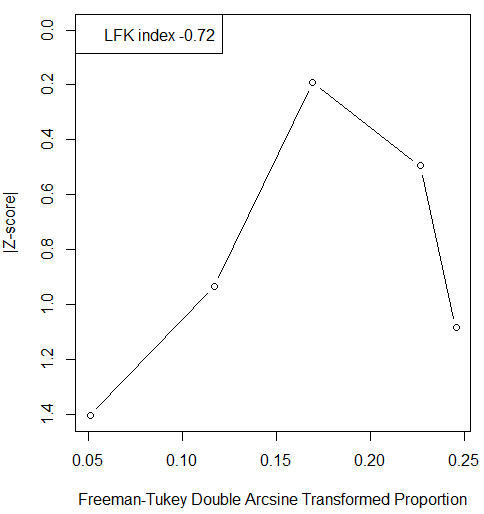 |

Abbreviations: LFK, Luis Furuya-Kanamori; STI, Sexually transmitted infection.

A minimum of three studies was required to perform this assessment.

# **Figure S3.** Doi plots assessing publication bias among studies reporting oropharyngeal *Neisseria gonorrhoeae* prevalence in Canada.

| 1. Men who have sex with men | 1. STI clinic attendees |
| --- | --- |
| 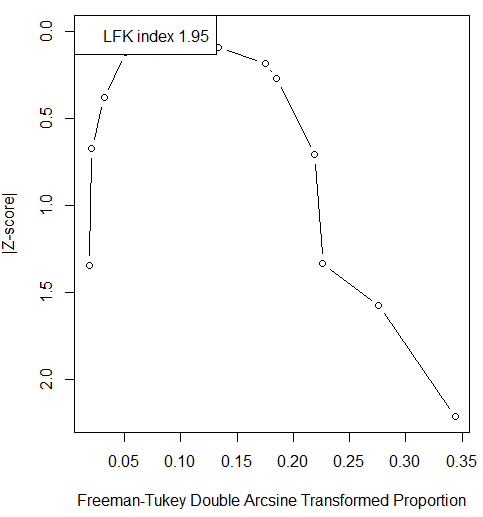 | 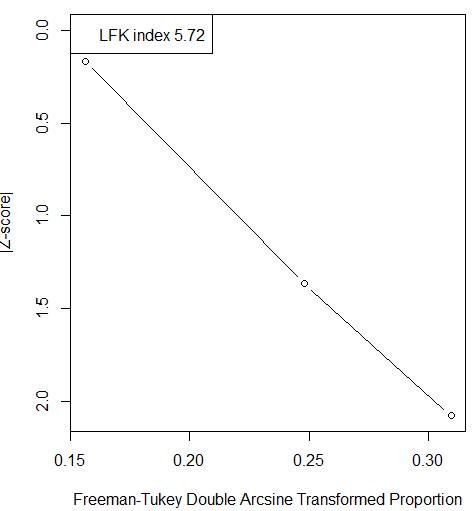 |

Abbreviations: LFK, Luis Furuya-Kanamori; STI, Sexually transmitted infection.

A minimum of three studies was required to perform this assessment.

# **Figure S4.** Doi plot assessing publication bias among studies reporting serological (ever-infection) *Neisseria gonorrhoeae* prevalence in Canada.

| 1. General populations |
| --- |
| 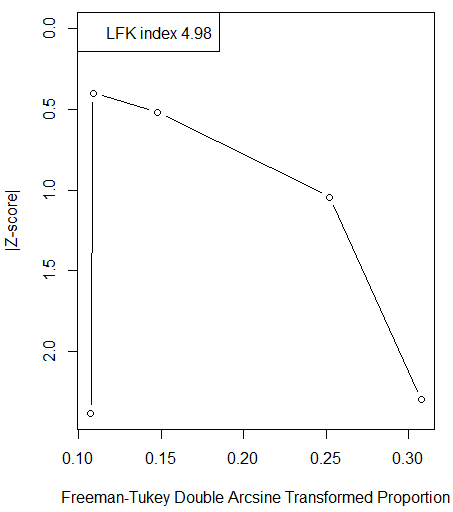 |

Abbreviations: LFK, Luis Furuya-Kanamori.

A minimum of three studies was required to perform this assessment.

# **Figure S5.** Doi plots assessing publication bias among studies reporting *Neisseria gonorrhoeae* prevalence in unspecified/mixed specimens Canada.

| 1. General populations | 1. Intermediate-risk populations | 1. Men who have sex with men |
| --- | --- | --- |
| 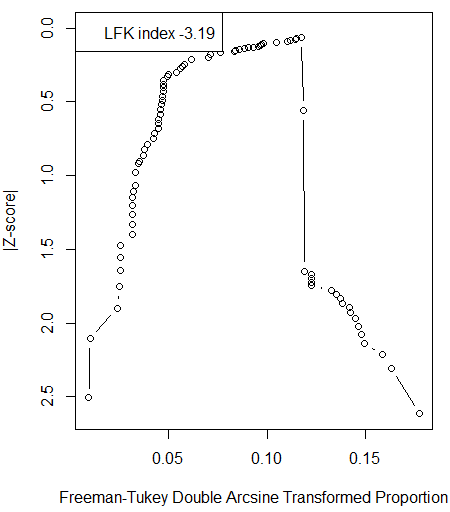 | 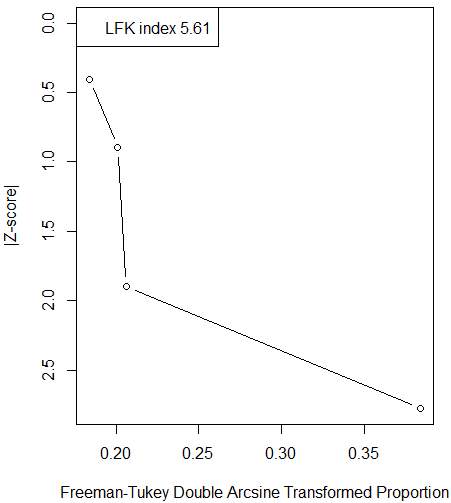 | 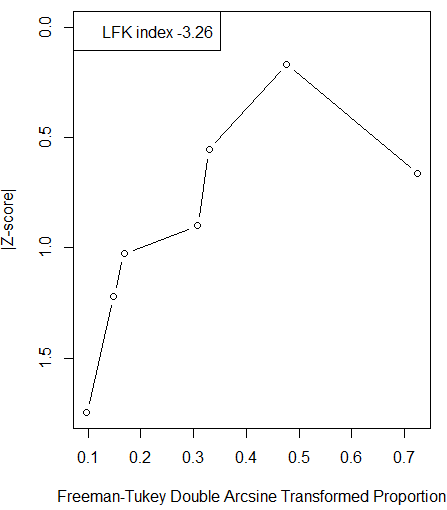 |
| 1. STI clinic attendees | 1. Patients with confirmed or suspected STIs and related infections |  |
| 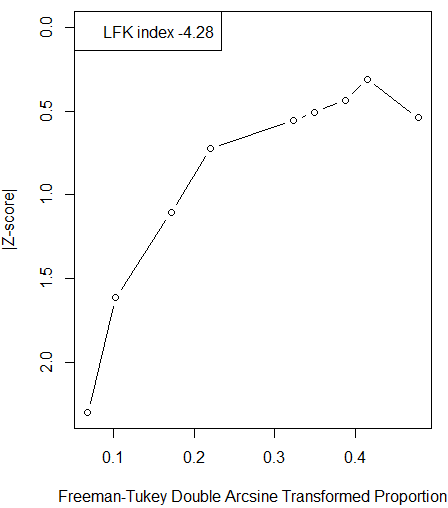 | 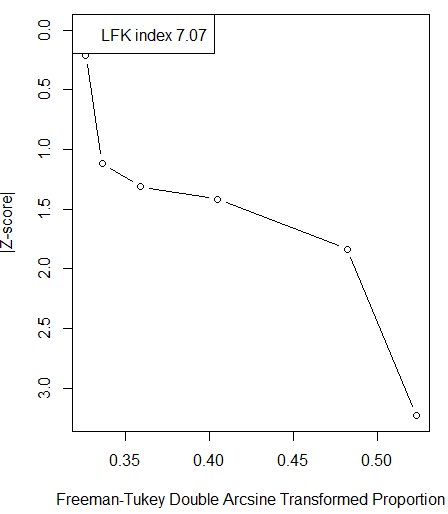 |  |

Abbreviations: HIV, Human immunodeficiency virus; LFK, Luis Furuya-Kanamori; STI, Sexually transmitted infection.

A minimum of three studies was required to perform this assessment.

# **Table S7.** Pooled mean prevalence of *Neisseria gonorrhoeae* in Canada, stratified by anatomical site, population type, assay type.

| **Population type** | **Stratified prevalence measures** | **Sample** | **NG prevalence (%)** | | **Pooled mean NG prevalence** | **Heterogeneity measures** | | |
| --- | --- | --- | --- | --- | --- | --- | --- | --- |
|  | **Total n** | **Total N** | **Range** | **Median** | **Mean (%)**  **(95% CI)** | **Q**^*^  **(p-value)** | **I²^†^ (%)**  **(95% CI)** | **Prediction interval^§^ (%)** |
| **Urogenital specimens** | | | | | | | | |
| **General populations** |  |  |  |  |  |  |  |  |
| NAAT/PCR | 26 | 241,272 | 0.0-6.1 | 1.0 | 1.2 (0.6-1.9) | 2,676.4 (p<0.001) | 99.1 (98.9-99.2) | 0.0-6.5 |
| Culture | 25 | 111,060 | 1.0-11.9 | 0.5 | 0.8 (0.2-1.7) | 808.1 (p<0.001) | 97.0 (96.3-97.6) | 0.0-7.5 |
| Mixed/unclear assay | 2 | 1,091 | 0.0-1.8 | 0.9 | 0.1 (0.0-0.5) | - | - | - |
| **Overall** | **53** | **353,423** | **0.0-11.9** | **0.8** | **1.0 (0.5-1.5)** | **3,496.0 (p<0.001)** | **98.5 (98.4-98.7)** | **0.0-6.6** |
| **Intermediate-risk populations** | | |  |  |  |  |  |  |
| NAAT/PCR | 21 | 9,884 | 0.0-10.8 | 1.2 | 1.4 (0.5-2.6) | 326.8 (p<0.001) | 93.9 (91.9-95.4) | 0.0-8.9 |
| Culture | 10 | 1,293 | 1.2-18.0 | 11.6 | 9.4 (6.1-13.2) | 35.7 (p<0.001) | 74.8 (53.0-86.5) | 0.9-23.9 |
| Mixed/unclear assay | 4 | 985 | 0.0-1.7 | 1.0 | 0.7 (0.0-1.9) | 7.6 (p=0.056) | 60.3 (0.0-86.7) | 0.0-5.6 |
| **Overall** | **35** | **12,162** | **0.0-18.0** | **1.7** | **2.7 (1.4-4.3)** | **526.0 (p<0.001)** | **93.5 (91.9-94.8)** | **0.0-15.6** |
| **Men who have sex with men** |  |  |  |  |  |  |  |  |
| NAAT/PCR | 14 | 5,373 | 0.0-8.3 | 0.5 | 0.9 (0.2-1.9) | 91.2 (p<0.001) | 85.8 (77.7-90.9) | 0.0-6.2 |
| **Overall** | **14** | **5,373** | **0.0-8.3** | **0.5** | **0.9 (0.2-1.9)** | **91.2 (p<0.001)** | **85.8 (77.7-90.9)** | **0.0-6.2** |
| **Symptomatic women** |  |  |  |  |  |  |  |  |
| Culture | 9 | 901 | 0.0-72.8 | 16.7 | 15.9 (4.0-33.2) | 383.3 (p<0.001) | 97.9 (97.1-98.5) | 0.0-83.8 |
| **Overall** | **9** | **901** | **0.0-72.8** | **16.7** | **15.9 (4.0-33.2)** | **383.3 (p<0.001)** | **97.9 (97.1-98.5)** | **0.0-83.8** |
| **Symptomatic men** |  |  |  |  |  |  |  |  |
| Culture | 3 | 489 | 22.9-82.6 | 23.0 | 42.9 (8.4-82.1) | 192.6 (p<0.001) | 99.0 (98.3-99.4) | 0.0-100 |
| Gram stain | 1 | 100 | - | - | 22.0 (14.3-31.4) | - | - | - |
| **Overall** | **4** | **589** | **22.0-82.6** | **22.9** | **37.4 (11.1-68.6)** | **217.2 (p<0.001)** | **98.6 (97.3-99.1)** | **0.0-100** |
| **Infertility clinic attendees** |  |  |  |  |  |  |  |  |
| NAAT/PCR | 5 | 5,589 | 0.0-0.1 | 0.0 | 0.0 (0.0-0.0) | 0.9 (p=0.925) | 0.0 (0.0-79.2) | 0.0-0.0 |
| **Overall** | **5** | **5,589** | **0.0-0.1** | **0.0** | **0.0 (0.0-0.0)** | **0.9 (p=0.925)** | **0.0 (0.0-79.2)** | **0.0-0.0** |
| **Women with miscarriage or ectopic pregnancy** | | |  |  |  |  |  |  |
| Culture | 1 | 52 | - | - | 0.0 (0.0-6.84) | - | - | - |
| **Overall** | **1** | **52** | **-** | **-** | **0.0 (0.0-6.84)** | **-** | **-** | **-** |
| **STI clinic attendees** |  |  |  |  |  |  |  |  |
| NAAT/PCR | 10 | 72,221 | 0.1-4.0 | 1.9 | 1.5 (0.9-2.4) | 272.6 (p<0.001) | 96.7 (95.3-97.7) | 0.0-5.3 |
| Culture | 16 | 6,401 | 0.7-47.3 | 15.9 | 13.3 (8.1-19.6) | 681.2 (p<0.001) | 97.8 (97.2-98.3) | 0.0-45.9 |
| **Overall** | **26** | **78,622** | **0.1-47.6** | **4.2** | **7.5 (4.2-11.6)** | **1,822.7 (p<0.001)** | **98.6 (98.4-98.8)** | **0.0-37.3** |
| **Individuals living with HIV and individuals in HIV-discordant couples** | | |  |  |  |  |  |  |
| NAAT/PCR | 5 | 3,702 | 0.0-2.0 | 0.3 | 0.6 (0.0-1.8) | 10.2 (p=0.037) | 60.9 (0.0-85.3) | 0.0-4.6 |
| **Overall** | **5** | **3,702** | **0.0-2.0** | **0.3** | **0.6 (0.0-1.8)** | **10.2 (p=0.037)** | **60.9 (0.0-85.3)** | **0.0-4.6** |
| **Sexual contacts of persons with NG or CT infection** | | | |  |  |  |  |  |
| Culture | 1 | 133 | - | - | 47.4 (38.7-56.2) | - | - | - |
| **Overall** | **1** | **133** | **-** | **-** | **47.4 (38.7-56.2)** | **-** | **-** | **-** |
| **Other populations^¶^** |  |  |  |  |  |  |  |  |
| NAAT/PCR | 5 | 12,059 | 1.7-4.4 | 3.0 | 2.1 (1.2-3.1) | 12.9 (p=0.012) | 69.0 (20.5-87.9) | 0.2-5.3 |
| Culture | 23 | 7,404 | 0.4-47.2 | 10.7 | 8.5 (5.2-12.4) | 618.8 (p<0.001) | 96.4 (95.5-97.2) | 0.0-33.2 |
| **Overall** | **28** | **19,463** | **0.0-47.2** | **6.6** | **7.2 (4.5-10.4)** | **1,046.7 (p<0.001)** | **97.4 (96.8-97.8)** | **0.0-29.6** |
| **Anorectal specimens** | | | | | | | | |
| **Men who have sex with men** |  |  |  |  |  |  |  |  |
| NAAT/PCR | 8 | 1,903 | 0.0-10.9 | 3.0 | 4.3 (1.9-7.3) | 31.9 (p<0.001) | 78.1 (56.8-88.9) | 0.0-15.6 |
| Culture | 4 | 1,450 | 0.2-5.8 | 0.3 | 0.9 (0.0-3.0) | 22.7 (p<0.00) | 86.8 (68.1-94.5) | 0.0-20.4 |
| Mixed/unclear assay | 1 | 25 | - | - | 4.0 (0.1-20.4) | - | - | - |
| **Overall** | **13** | **3,378** | **0.0-10.9** | **2.9** | **2.6 (0.9-4.9)** | **99.4 (p<0.001)** | **87.9 (81.1-92.3)** | **0.0-14.1** |
| **STI clinic attendees** |  |  |  |  |  |  |  |  |
| NAAT/PCR | 5 | 3,500 | 0.2-5.9 | 2.8 | 2.5 (0.7-5.3) | 57.9 (p<0.001) | 93.1 (86.8-96.4) | 0.0-15.1 |
| **Overall** | **5** | **3,500** | **0.2-5.9** | **2.8** | **2.5 (0.7-5.3)** | **57.9 (p<0.001)** | **93.1 (86.8-96.4)** | **0.0-15.1** |
| **Patients with confirmed or suspected STIs and related infections** | | |  |  |  |  |  |  |
| NAAT/PCR | 2 | 432 | 2.6-13.3 | 8.0 | 8.6 (6.1-11.6) | - | - | - |
| **Overall** | **2** | **432** | **2.6-13.3** | **8.0** | **8.6 (6.1-11.6)** | **-** | **-** | **-** |
| **Oropharyngeal specimens** | | | | | | | | |
| **Men who have sex with men** | |  |  |  |  |  |  |  |
| NAAT/PCR | 8 | 1,947 | 0.0-11.0 | 3.2 | 4.1 (2.2-6.4) | 15.4 (P=0.0314) | 54.5 (0.0-79.5) | 0.1-11.7 |
| Culture | 4 | 2,124 | 0.0-0.2 | 0.0 | 0.0 (0.0-0.2) | 1.5 (P=0.673) | 0.0 (0.0-84.7) | 0.0-0.5 |
| **Overall** | **12** | **4,071** | **0.0-11.0** | **2.0** | **1.6 (0.2-3.8)** | **49.5 (P<0.001)** | **93.5 (90.4-95.6)** | **0.0-13.9** |
| **STI clinic attendees** |  |  |  |  |  |  |  |  |
| NAAT/PCR | 3 | 2,988 | 2.4-8.9 | 5.9 | 5.0 (1.8-9.6) | 17.9 (p<0.001) | 88.8 (69.2-95.9) | 0.0-31.2 |
| **Overall** | **3** | **2,988** | **2.4-8.9** | **5.9** | **5.0 (1.8-9.6)** | **17.9 (p<0.001)** | **88.8 (69.2-95.9)** | **0.0-31.2** |
| **Serological specimens** | | | | | | | | |
| **General populations** |  |  |  |  |  |  |  |  |
| ELISA | 2 | 338 | 6.0-7.0 | 6.5 | 6.2 (3.9-9.3) | - | - | - |
| Microflocculation test | 2 | 87 | 0.0-1.5 | 0.8 | 1.1 (0.0-6.2) | - | - | - |
| Indirect fluorescent test | 1 | 806 | - | - | 1.1 (0.5-2.1) | - | - | - |
| **Overall** | **5** | **1,231** | **0.0-7.0** | **1.5** | **2.3 (0.3-5.6)** | **21.4 (p<0.001)** | **81.3 (56.5-91.9)** | **0.0-14.6** |
| **Symptomatic men** |  |  |  |  |  |  |  |  |
| Indirect fluorescent test | 1 | 205 | - | - | 2.9 (1.1-6.3) | - | - | - |
| **Overall** | **1** | **205** | **-** | **-** | **2.9 (1.1-6.3)** | **-** | **-** | **-** |
| **STI clinic attendees** |  |  |  |  |  |  |  |  |
| Indirect fluorescent test | 1 | 156 | - | - | 35.3 (27.8-43.3) | **-** | **-** | **-** |
| **Overall** | **1** | **156** | **-** | **-** | **35.3 (27.8-43.3)** | **-** | **-** | **-** |
| **Patients with confirmed or suspected STIs and related infections** | | |  |  |  |  |  |  |
| ELISA | 1 | 169 | - | - | 79.9 (73.0-85.6) | - | - | - |
| **Overall** | **1** | **169** | **-** | **-** | **79.9 (73.0-85.6)** | **-** | **-** | **-** |
| **Other populations^¶^** |  |  |  |  |  |  |  |  |
| Microflocculation test | 2 | 885 | 14.3-19.4 | 7.9 | 19.2 (16.7-22.0) | - | - | - |
| **Overall** | **2** | **885** | **14.3-19.4** | **7.9** | **19.2 (16.7-22.0)** | **-** | **-** | **-** |
| **Unspecified/mixed specimens** | | | | | | | | |
| **General populations** |  |  |  |  |  |  |  |  |
| NAAT/PCR | 75 | 111,225 | 0.0-3.1 | 0.1 | 0.5 (0.4-0.7) | 603.4 (p<0.001) | 87.7 (85.3-89.9) | 0.0-2.4 |
| Mixed/unclear assay | 4 | 109,207 | 0.0-1.4 | 0.1 | 0.3 (0.0-1.1) | 123.7 (p<0.001) | 97.6 (95.8-98.6) | 0.0-7.4 |
| **Overall** | **79** | **220,432** | **0.0-3.1** | **0.3** | **0.5 (0.4-0.6)** | **1,523.7 (p<0.001)** | **94.9 (94.1-95.5)** | **0.0-2.3** |
| **Intermediate-risk populations** | |  |  |  |  |  |  |  |
| NAAT/PCR | 2 | 5,517 | 3.4-4.0 | 3.7 | 3.5 (3.0-4.0) | - | - | - |
| Mixed/unclear assay | 2 | 3.4 | 4.0-12.9 | 8.5 | 4.9 (2.8-8.0) | - | - | - |
| **Overall** | **4** | **5,821** | **3.4-12.9** | **4.0** | **3.3 (2.7-3.9)** | **6.3 (p=0.098)** | **52.3 (0.0-84.2)** | **1.7-5.3** |
| **Men who have sex with men** |  |  |  |  |  |  |  |  |
| NAAT/PCR | 2 | 2,839 | 2.1-10.5 | 6.3 | 8.2 (7.3-9.3) | - | - | - |
| Culture | 1 | 1,466 | - | - | 21.0 (18.9-23.3) | - | - | - |
| Mixed/unclear assay | 4 | 8,064 | 1.0-43.9 | 5.9 | 10.1 (0.2-31.9) | 1,775.9 (p<0.001) | 99.8 (99.8-99.9) | 0.0-100 |
| **Overall** | **7** | **12,369** | **1.0-43.9** | **9.1** | **10.0 (2.4-21.8)** | **2,717.9 (p<0.001)** | **99.8 (99.7-99.8)** | **0.0-64.0** |
| **STI clinic attendees** |  |  |  |  |  |  |  |  |
| NAAT/PCR | 3 | 19,241 | 2.9-21.2 | 4.7 | 8.2 (1.1-21.1) | 1,169.7 (p<0.001) | 99.8 (99.8-99.9) | 0.0-80.2 |
| Culture | 2 | 1,275 | 11.8-14.2 | 13.0 | 13.0 (11.2-15.0) | - | - | - |
| Mixed/unclear assay | 4 | 3,903 | 0.4-16.2 | 5.4 | 5.0 (0.3-14.6) | 383.7 (p<0.001) | 99.2(98.9-99.5) | 0.0-54.0 |
| **Overall** | **9** | **24,419** | **0.4-21.2** | **9.9** | **7.6 (3.3-13.3)** | **1,922.8 (p<0.001)** | **99.6 (99.5-99.7)** | **0.0-.34.7** |
| **Individuals living with HIV and individuals in HIV-discordant couples** | | |  |  |  |  |  |  |
| NAAT/PCR | 1 | 578 | - | - | 0.7 (0.2-1.8) | - | - | - |
| Mixed/unclear assay | 1 | 1,997 | - | - | 2.3 (1.7-3.1) | - | - | - |
| **Overall** | **2** | **2,575** | **0.7-2.3** | **1.5** | **1.9 (1.4-2.6)** | **-** | **-** | **-** |
| **Sexual contacts of persons with NG or CT infection** | |  |  |  |  |  |  |  |
| NAAT/PCR | 2 | 429 | 25.0-25.0 | 25 | 20.3 (16.6-24.4) | - | - | - |
| **Overall** | **2** | **429** | **25.0-25.0** | **25** | **20.3 (16.6-24.4)** | **-** | **-** | **-** |
| **Patients with confirmed or suspected STIs and related infections** | | |  |  |  |  |  |  |
| Mixed/unclear assay | 6 | 17,636 | 10.3-24.0 | 13.7 | 14.1 (10.2-18.4) | 112.1 (p<0.001) | 95.5 (92.6-97.3) | 3.3-30.4 |
| **Overall** | **6** | **17,636** | **10.3-24.0** | **13.7** | **14.1 (10.2-18.4)** | **112.1 (p<0.001)** | **95.5 (92.6-97.3)** | **3.3-30.4** |
| **Other populations^¶^** |  |  |  |  |  |  |  |  |
| Culture | 1 | 211 | - | - | 1.3 (0.8-2.1) | - | - | - |
| Mixed/unclear assay | 1 | 19,618 | - | - | 15.3 (14.8-15.8) | - | - | - |
| **Overall** | **2** | **19,829** | **7.6-15.3** | **11.4** | **11.5 (5.1-19.9)** | **-** | **-** | **-** |

Abbreviations: CI, Confidence interval; CT*, Chlamydia trachomatis;* ELISA, enzyme-linked immunosorbent assay; HIV, Human immunodeficiency virus; NAAT, Nucleic acid amplification test; NG, *Neisseria gonorrhoeae*; PCR, Polymerase chain reaction; STI, Sexually transmitted infection.

A minimum of three studies was required to perform a meta-analysis.

^*^ Q: The Cochran’s Q statistic is a measure assessing the existence of heterogeneity in pooled outcome measures, here NG prevalence.

**^†^** I^2^: A measure that assesses the magnitude of between-study variation that is due to true differences in NG prevalence across studies rather than chance.

**^§^** Prediction interval: A measure that estimates the distribution (95% interval) of true NG prevalence around the estimated mean.

**^¶^** Other populations include groups with an undetermined risk of acquiring NG infection, such as cervical cancer patients, specimens submitted to virology or bacteriology laboratories, and mixed or undefined populations.

# **Figure S6****.** Forest plots presenting outcomes of the pooled mean *Neisseria gonorrhoeae* prevalence in urogenital specimens among different populations in Canada.

1. General populations


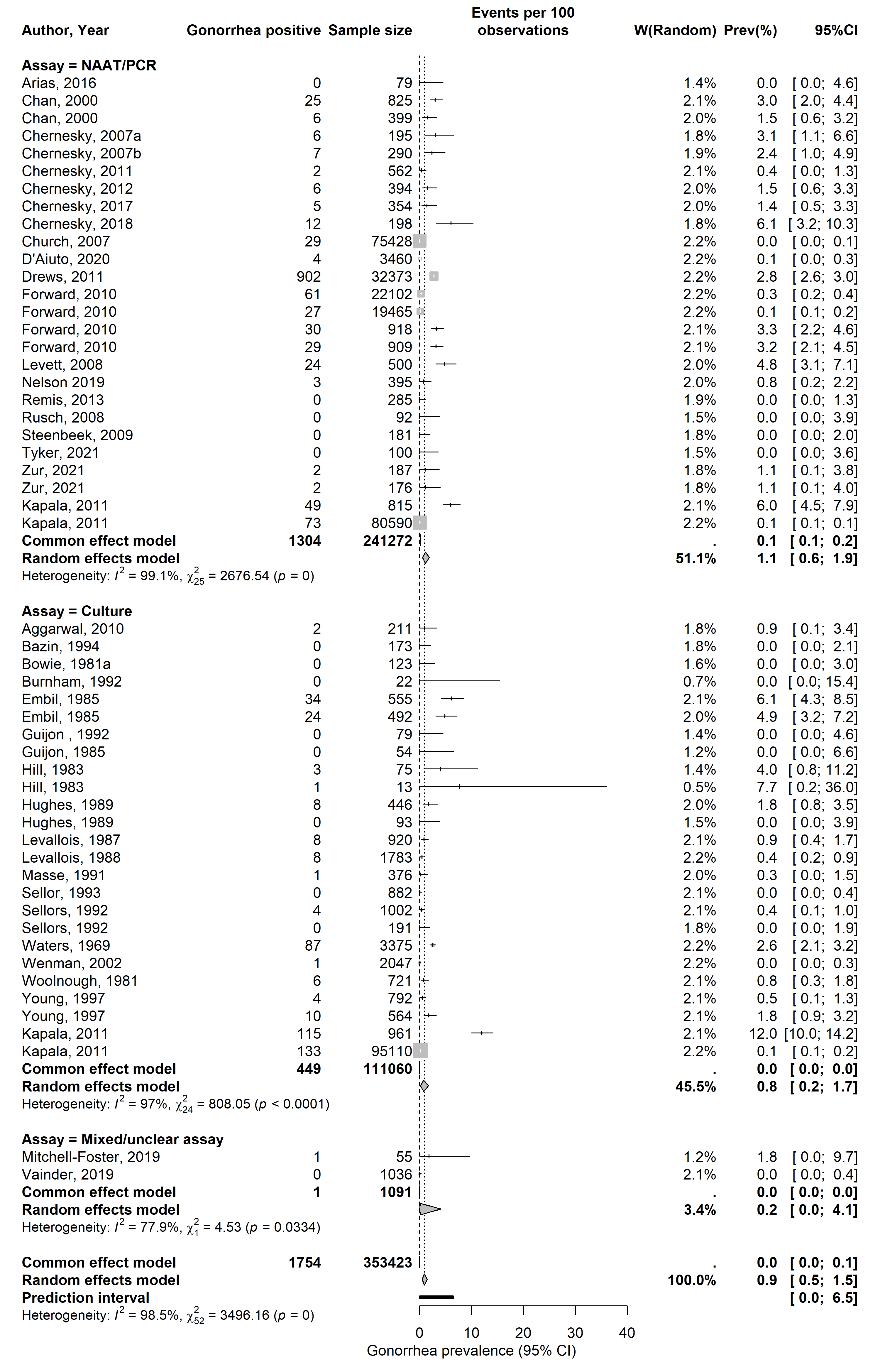


Abbreviation: CI, Confidence interval; NAAT, Nucleic acid amplification test; PCR, Polymerase chain reaction.

1.
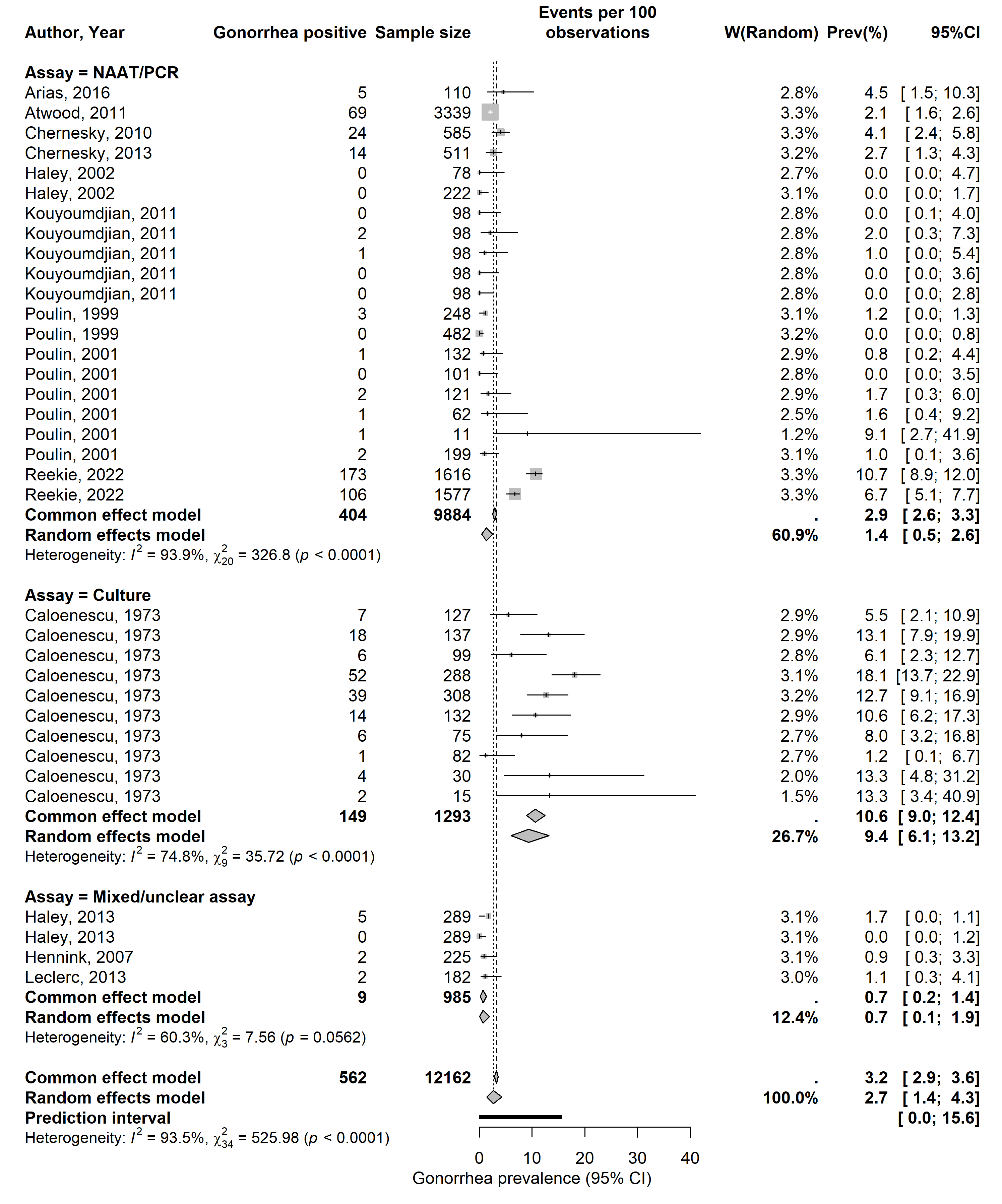
Intermediate-risk populations

Abbreviation: CI, Confidence interval; NAAT, Nucleic acid amplification test; PCR, Polymerase chain reaction.

1. Men who have sex with men


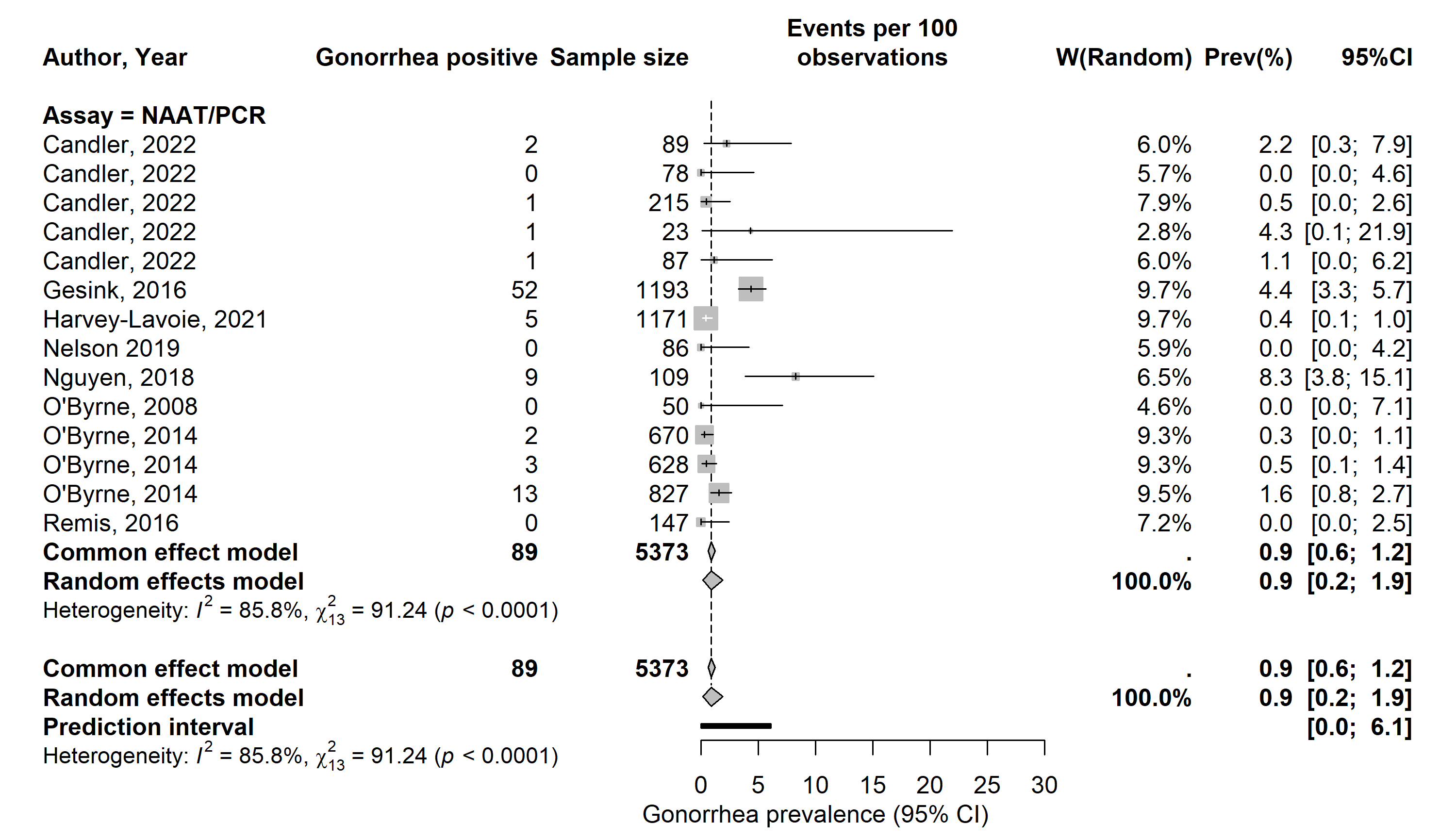


Abbreviation: CI, Confidence interval; NAAT, Nucleic acid amplification test; PCR, Polymerase chain reaction.

1. Symptomatic women
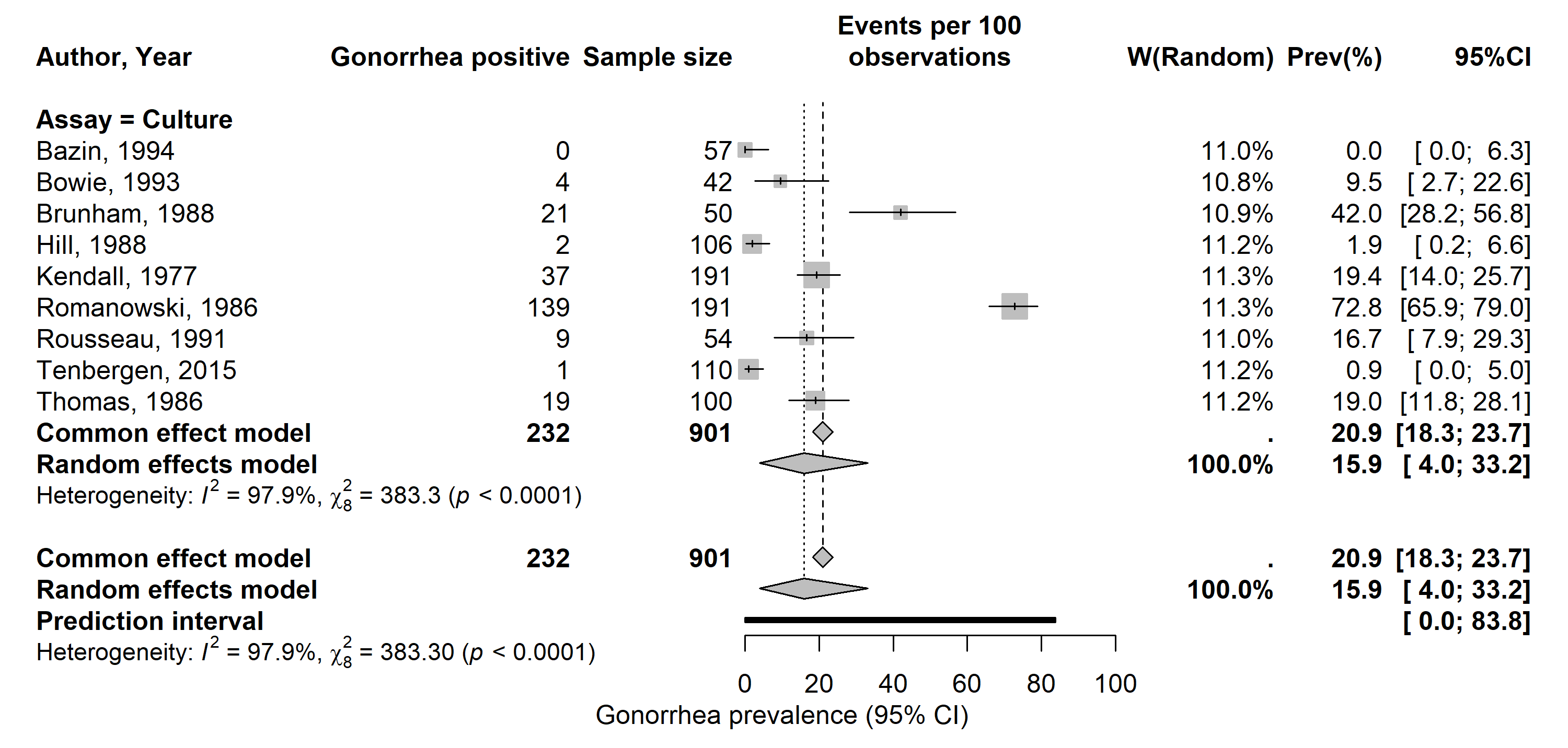


Abbreviation: CI, Confidence interval.

1. Symptomatic men


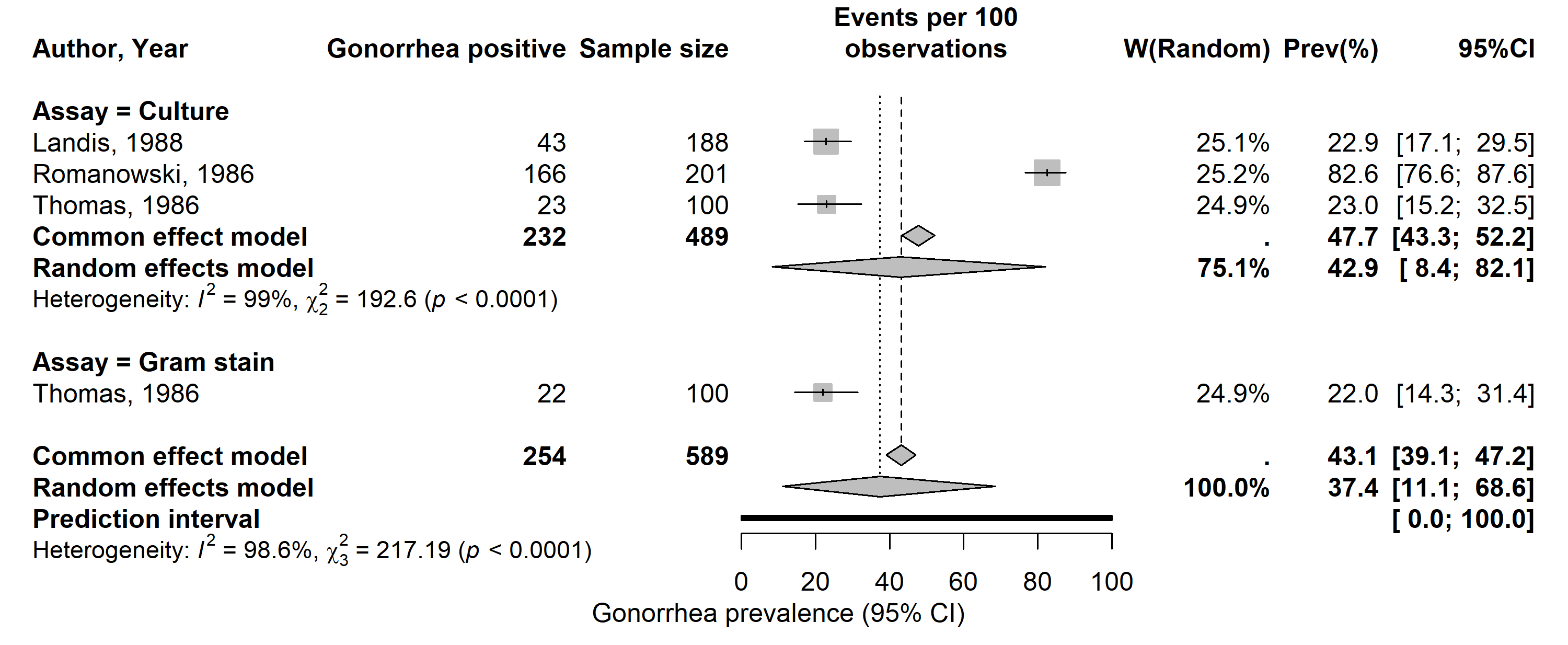


Abbreviation: CI, Confidence interval.

1. Infertility clinic attendees
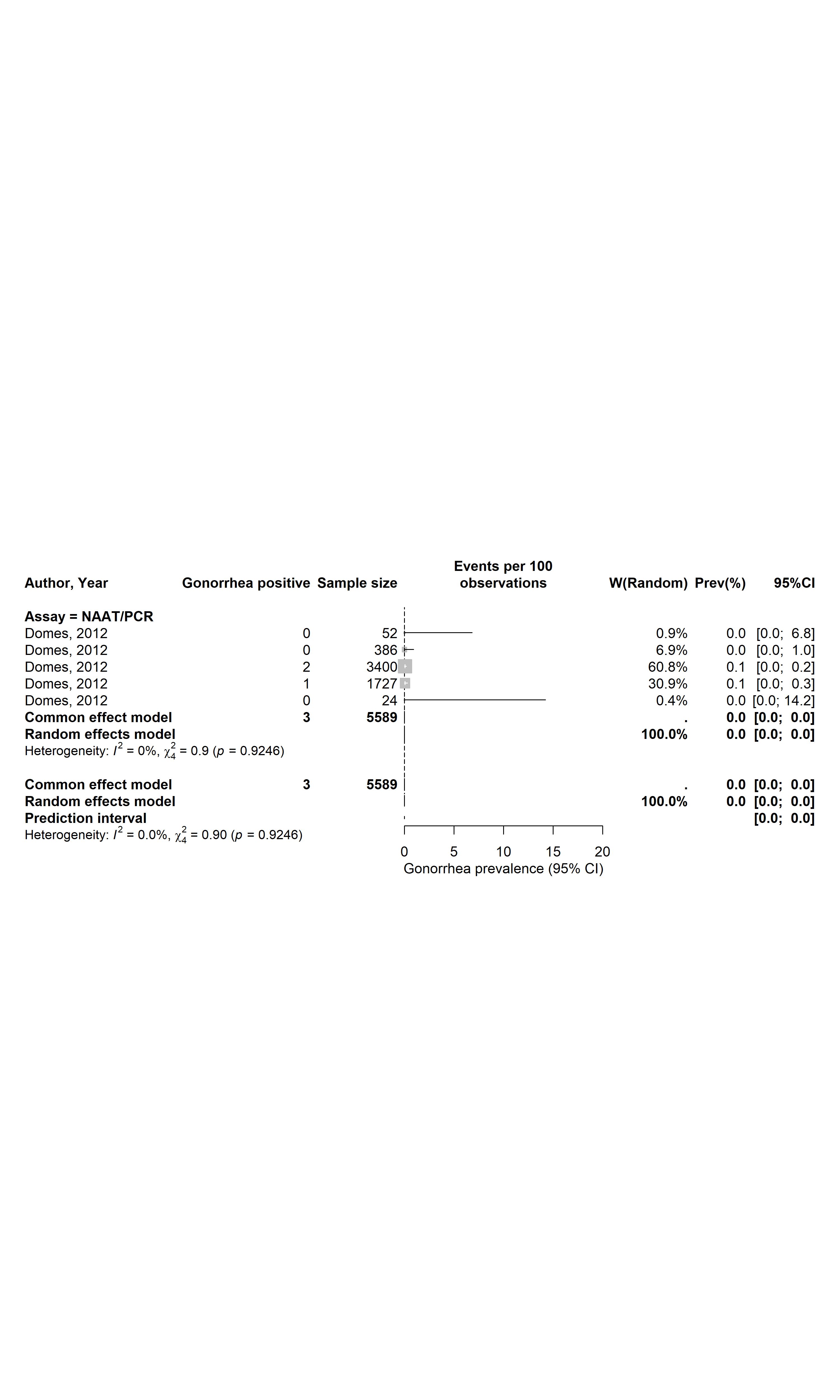


Abbreviation: CI, Confidence interval; NAAT, Nucleic acid amplification test; PCR, Polymerase chain reaction.

1. STI clinic attendees
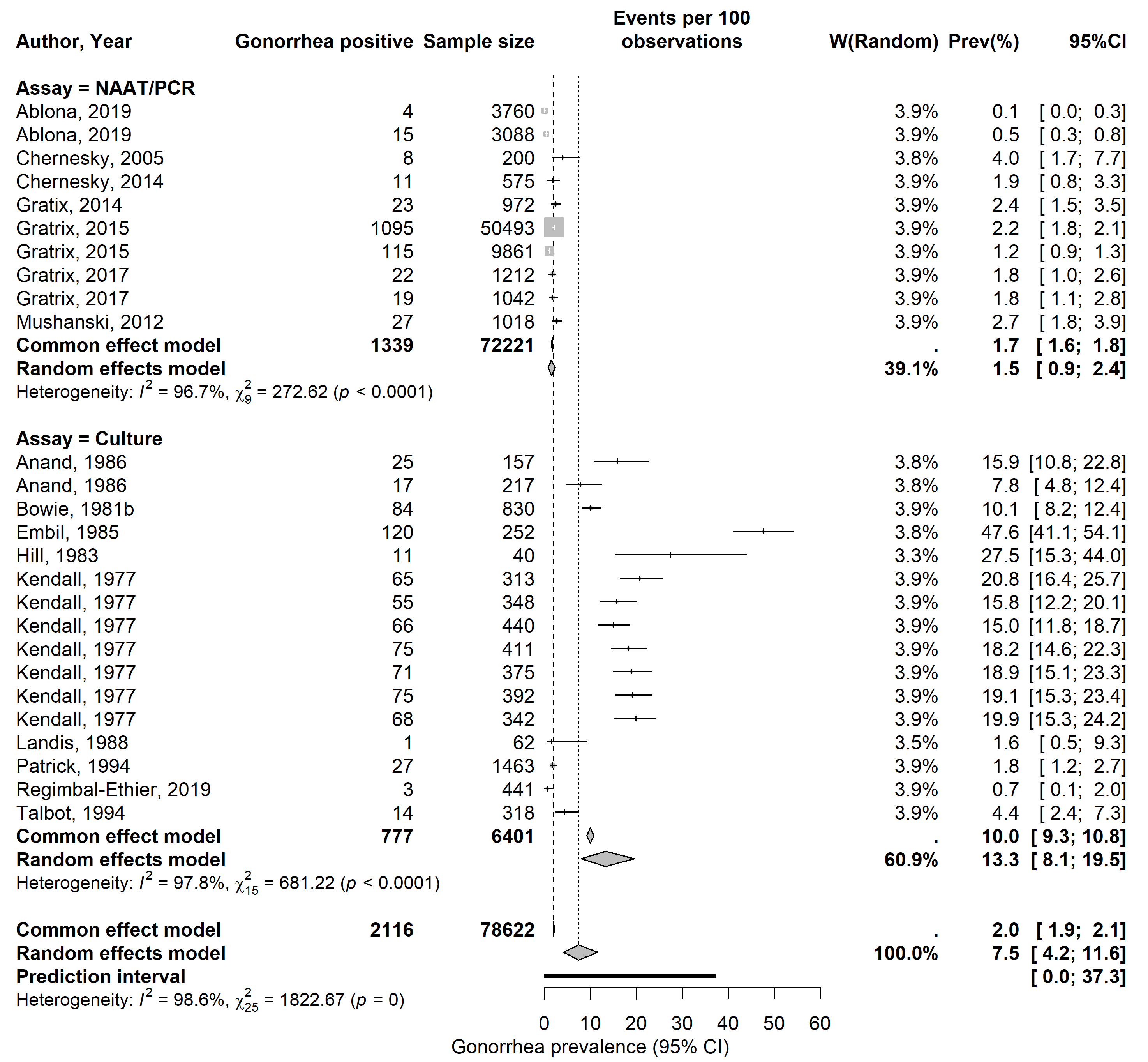


Abbreviations: CI, Confidence interval; NAAT, Nucleic acid amplification test; PCR, Polymerase chain reaction; STI, Sexually transmitted infection.

1. Individuals living with HIV and individuals in HIV-discordant couples
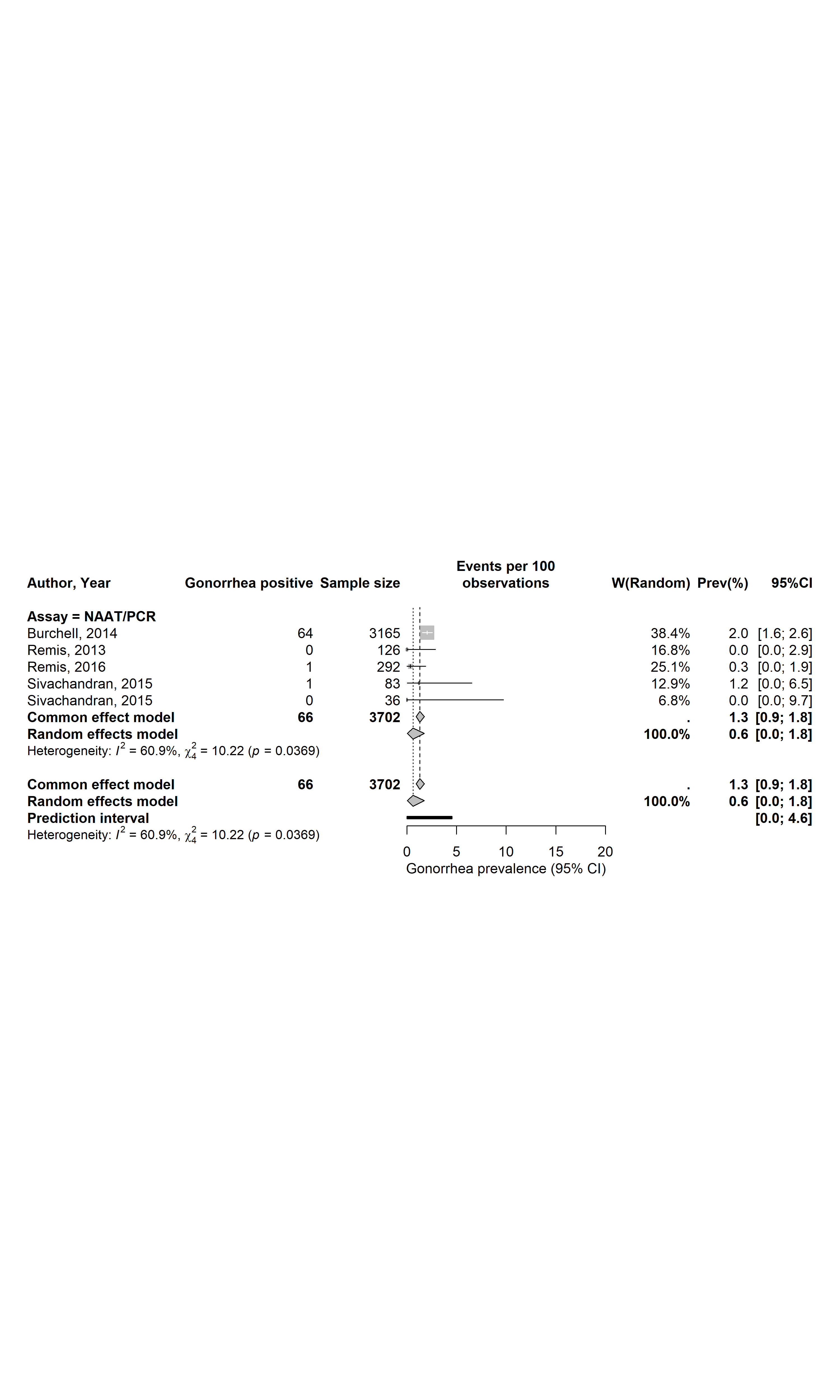


Abbreviations: CI, Confidence interval; HIV, Human immunodeficiency virus; NAAT, Nucleic acid amplification test; PCR, Polymerase chain reaction.

1. Other populations^*^


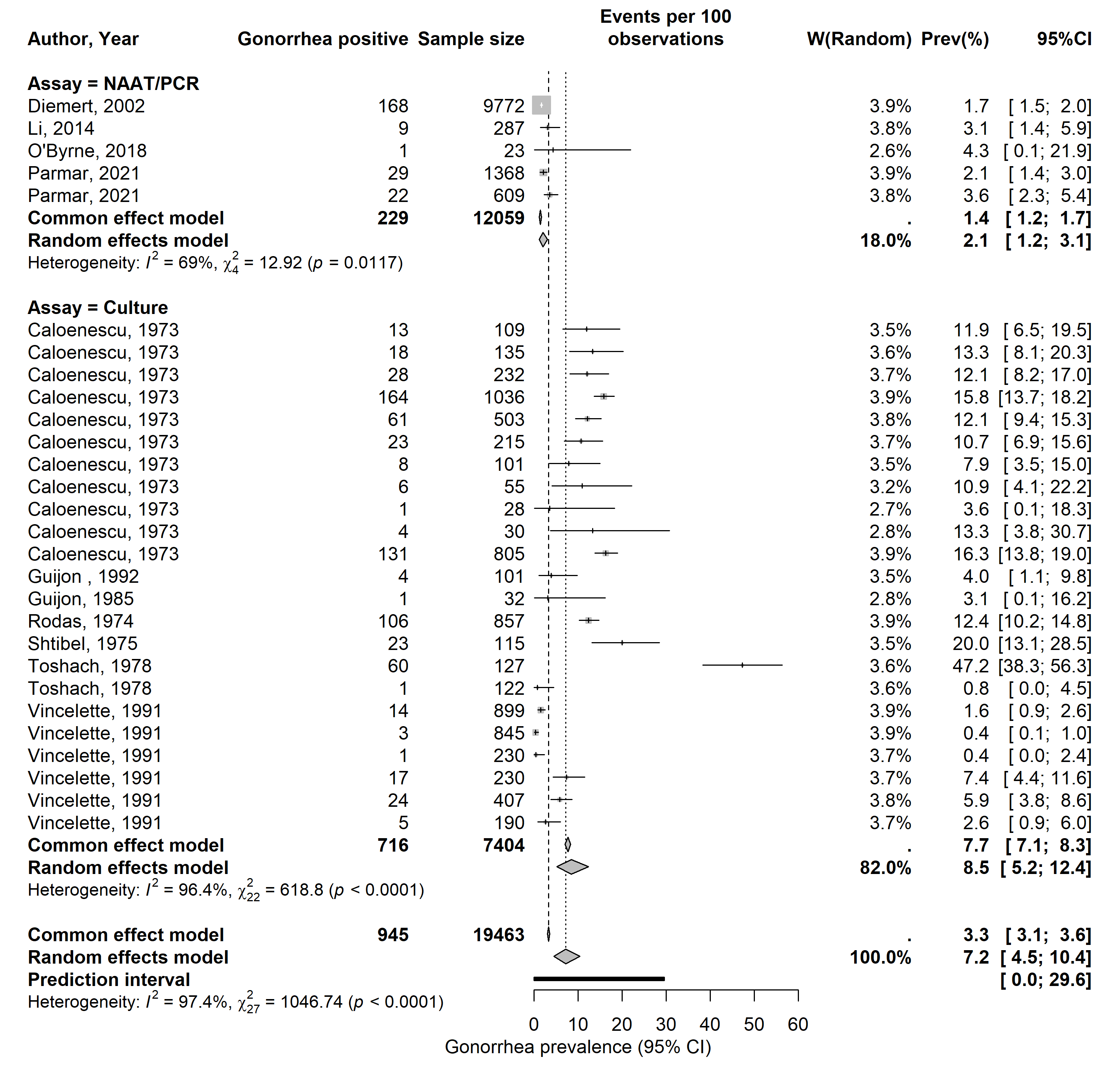
Abbreviations: CI, Confidence interval; NAAT, Nucleic acid amplification test; PCR, Polymerase chain reaction.

^*^Other populations include groups with an undetermined risk of acquiring NG infection, such as cervical cancer patients, specimens submitted to virology or bacteriology laboratories, and mixed or undefined populations.

# **Figure S7.** Forest plots presenting outcomes of the pooled mean *Neisseria gonorrhoeae* prevalence in anorectal specimens among different populations in Canada.

1. Men who have sex with men


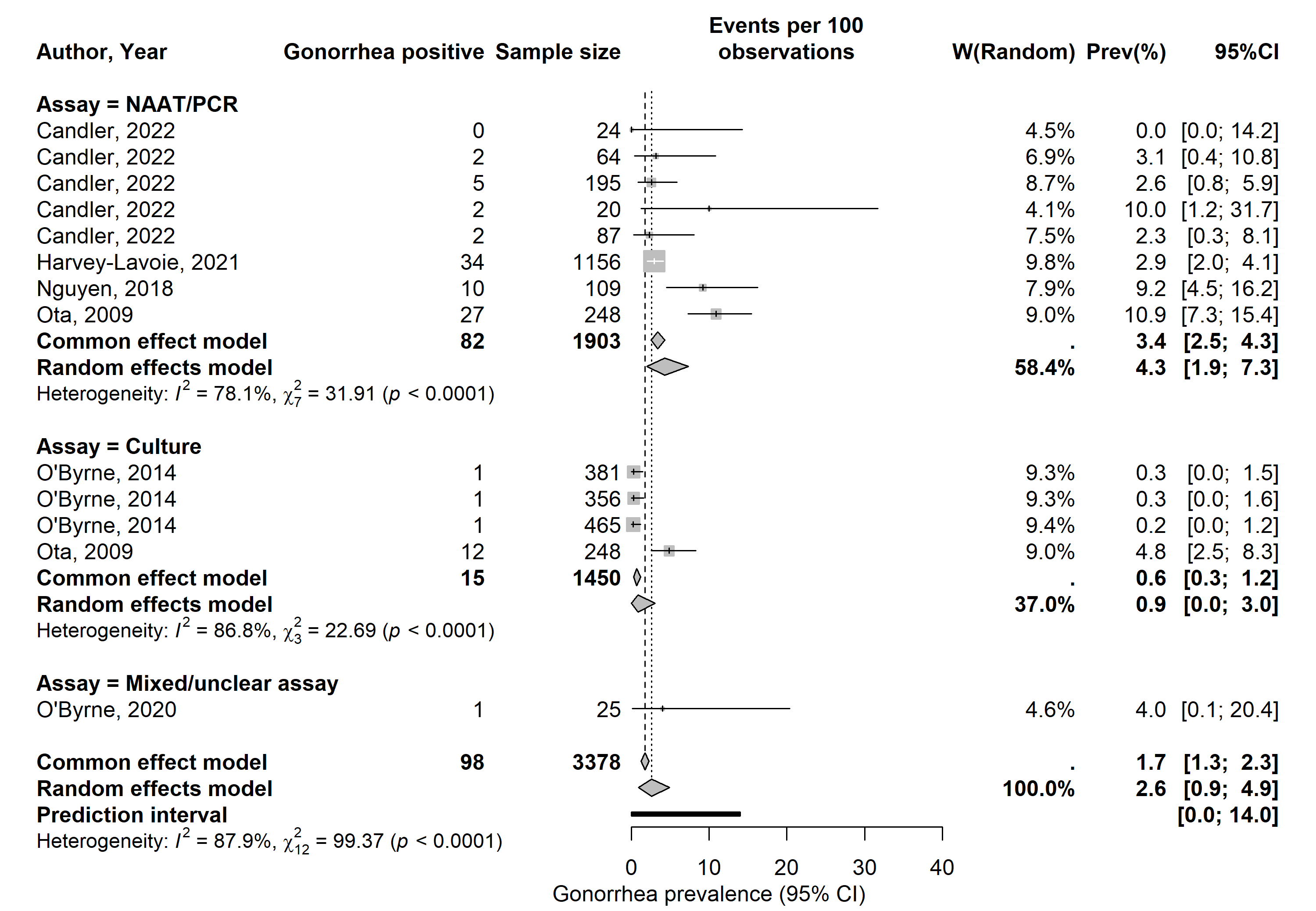


Abbreviations: CI, Confidence interval; NAAT, Nucleic acid amplification test; PCR, Polymerase chain reaction.

1. STI clinic attendees
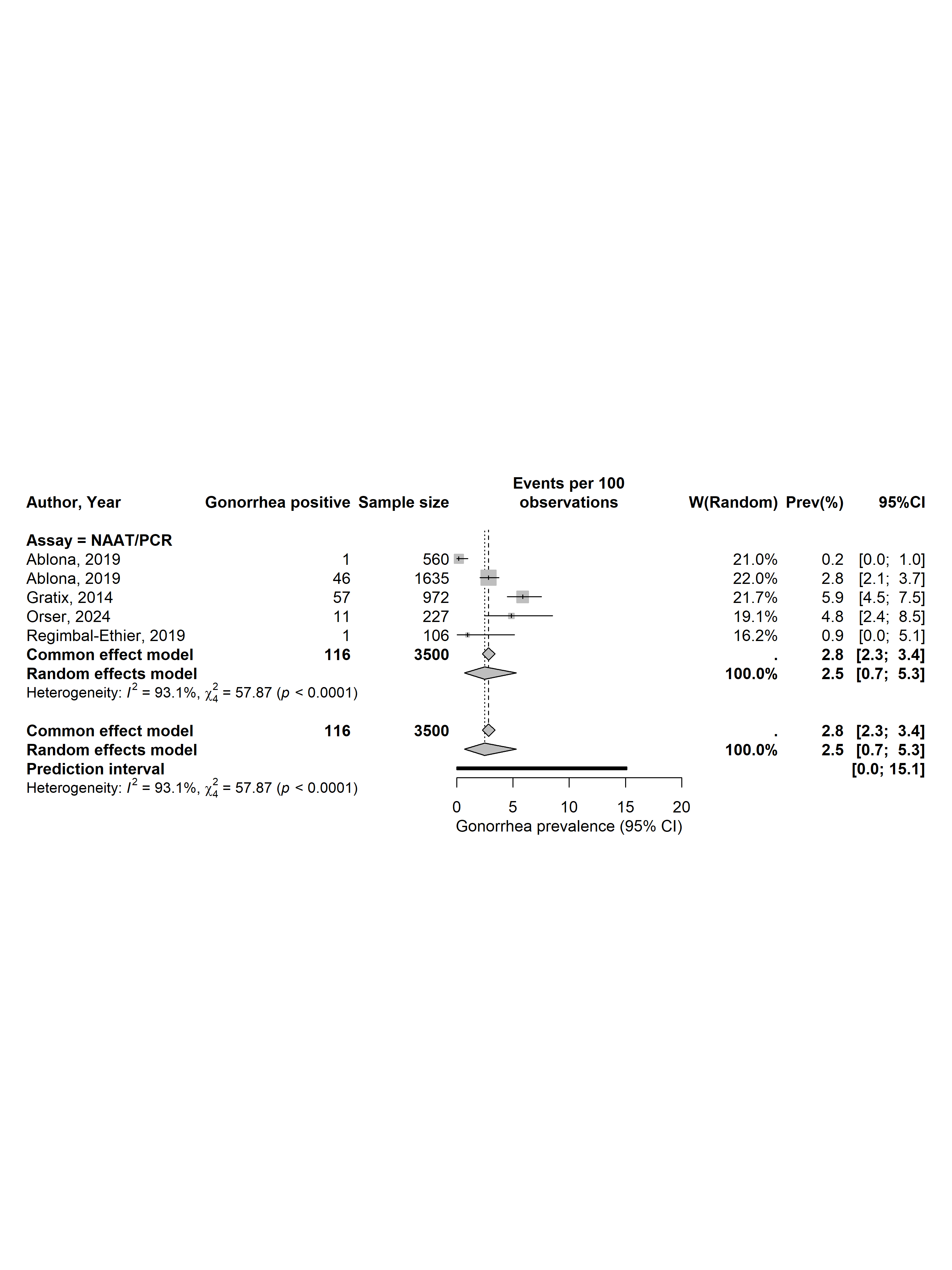


Abbreviations: CI, Confidence interval; NAAT, Nucleic acid amplification test; PCR, Polymerase chain reaction; STI, Sexually transmitted infection.

# **Figure S8.** Forest plots presenting outcomes of the pooled mean *Neisseria gonorrhoeae* prevalence in oropharyngeal specimens among different populations in Canada.

1. Men who have sex with men
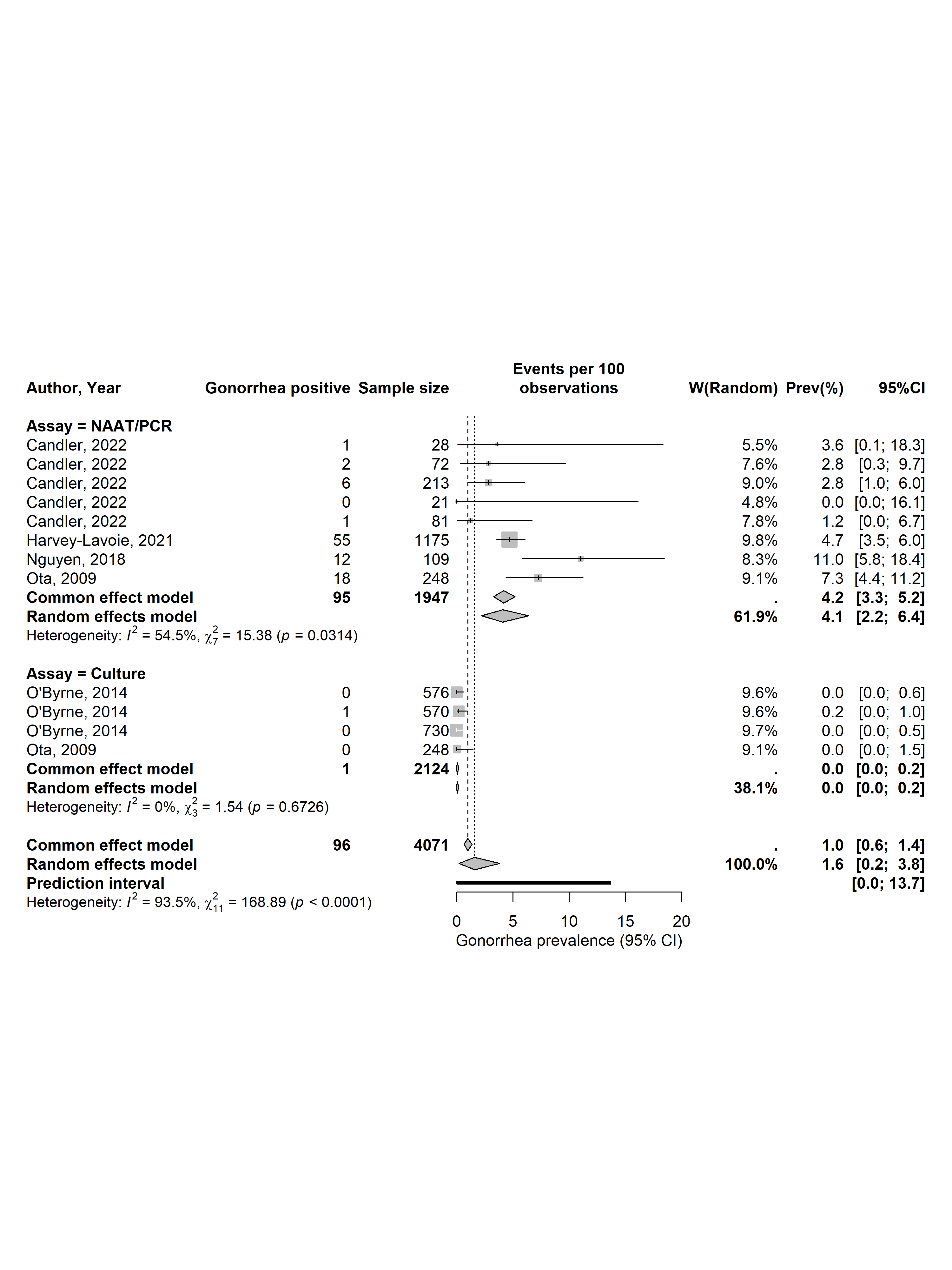


Abbreviations: CI, Confidence interval; NAAT, Nucleic acid amplification test; PCR, Polymerase chain reaction.

1. STI clinic attendees
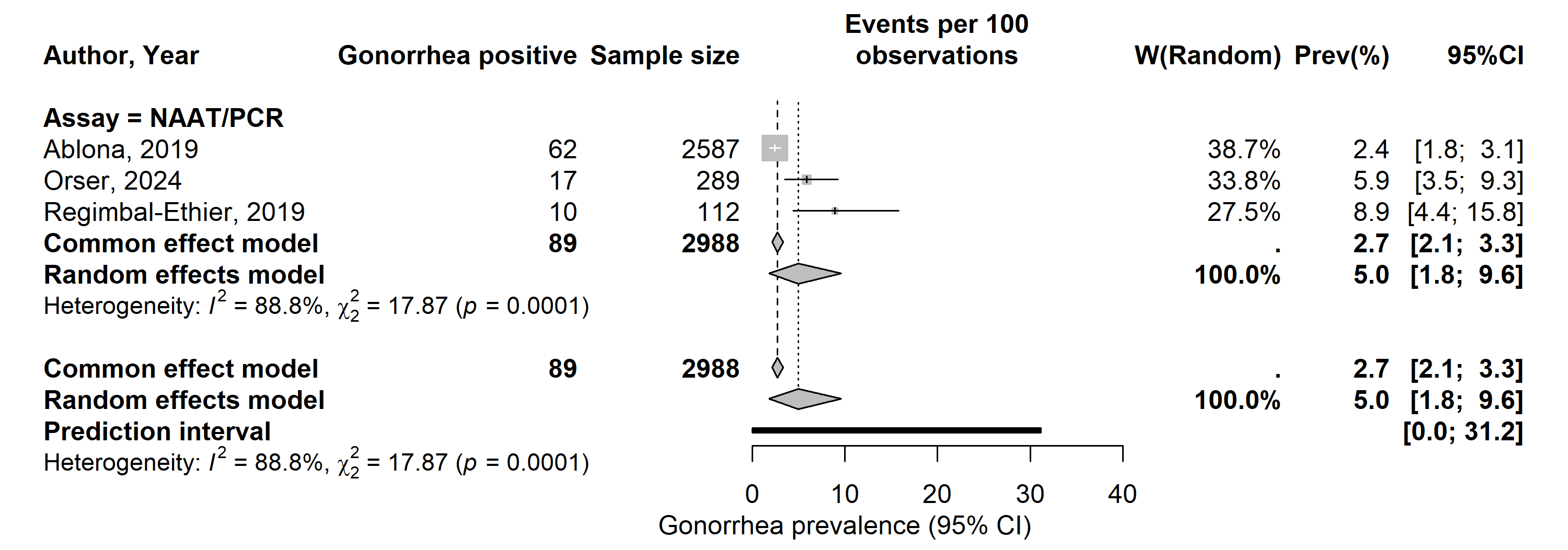


Abbreviations: CI, Confidence interval; NAAT, Nucleic acid amplification test; PCR, Polymerase chain reaction; STI, Sexually transmitted infection.

#
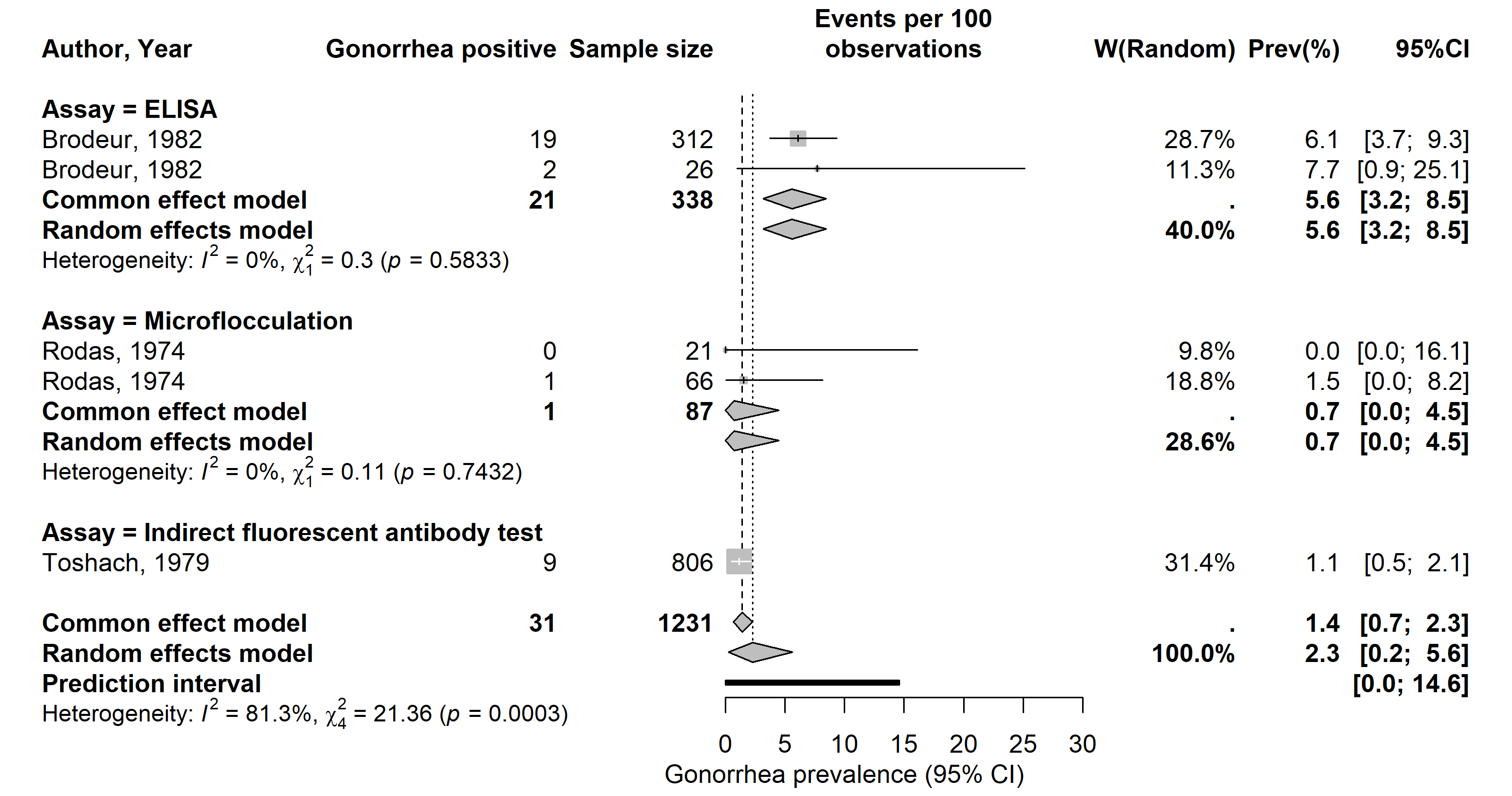
**Figure S9.** Forest plots presenting outcomes of the pooled mean *Neisseria gonorrhoeae* prevalence in serological specimens among general populations in Canada.

Abbreviations: CI, Confidence interval.

# **Figure S10.** Forest plots presenting outcomes of the pooled mean *Neisseria gonorrhoeae* prevalence in unspecified/mixed specimens among different populations in Canada.

1.
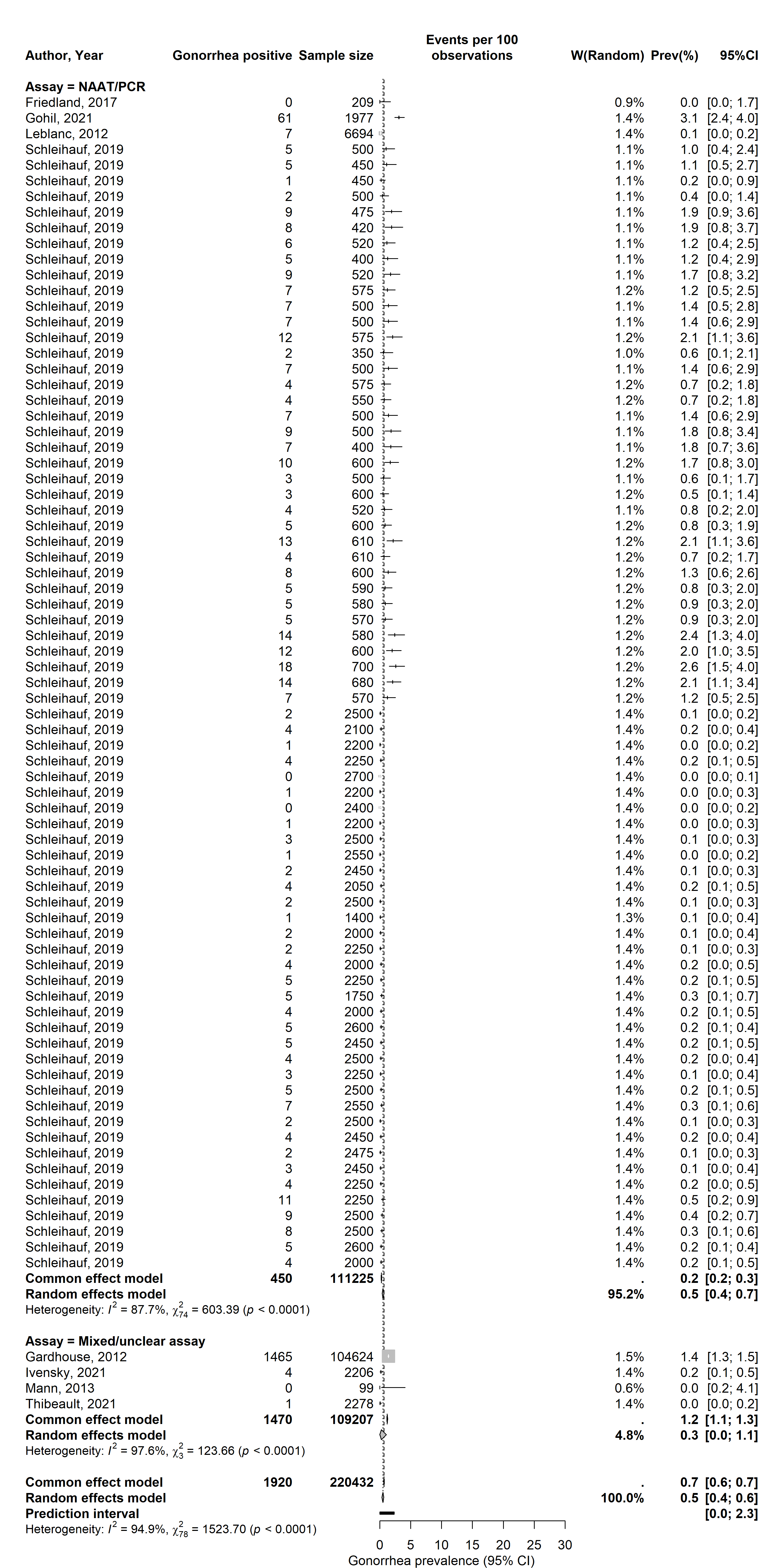
General populations

Abbreviations: CI, Confidence interval; NAAT, Nucleic acid amplification test; PCR, Polymerase chain reaction.

1.
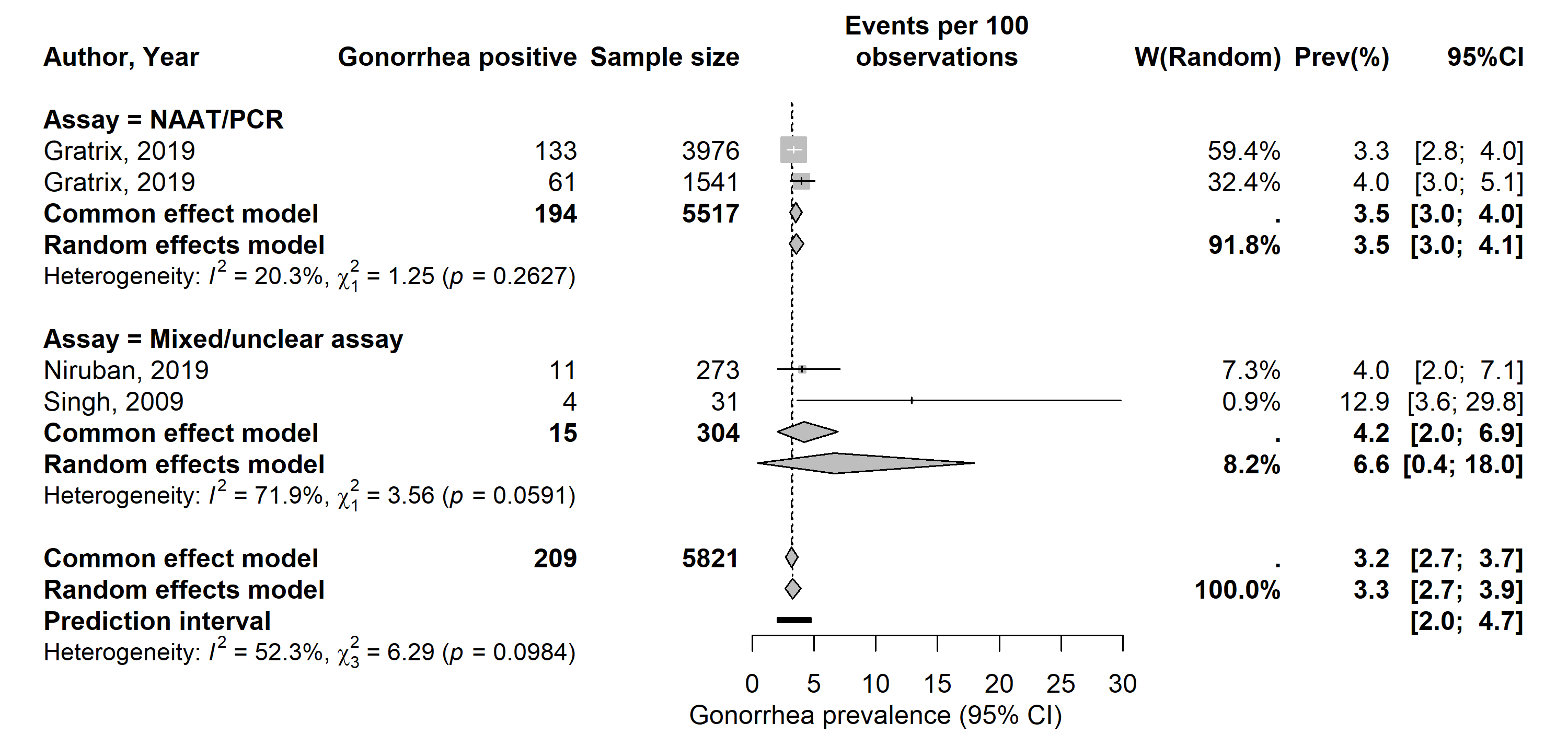
Intermediate-risk populations

Abbreviations: CI, Confidence interval; NAAT, Nucleic acid amplification test; PCR, Polymerase chain reaction.

1.
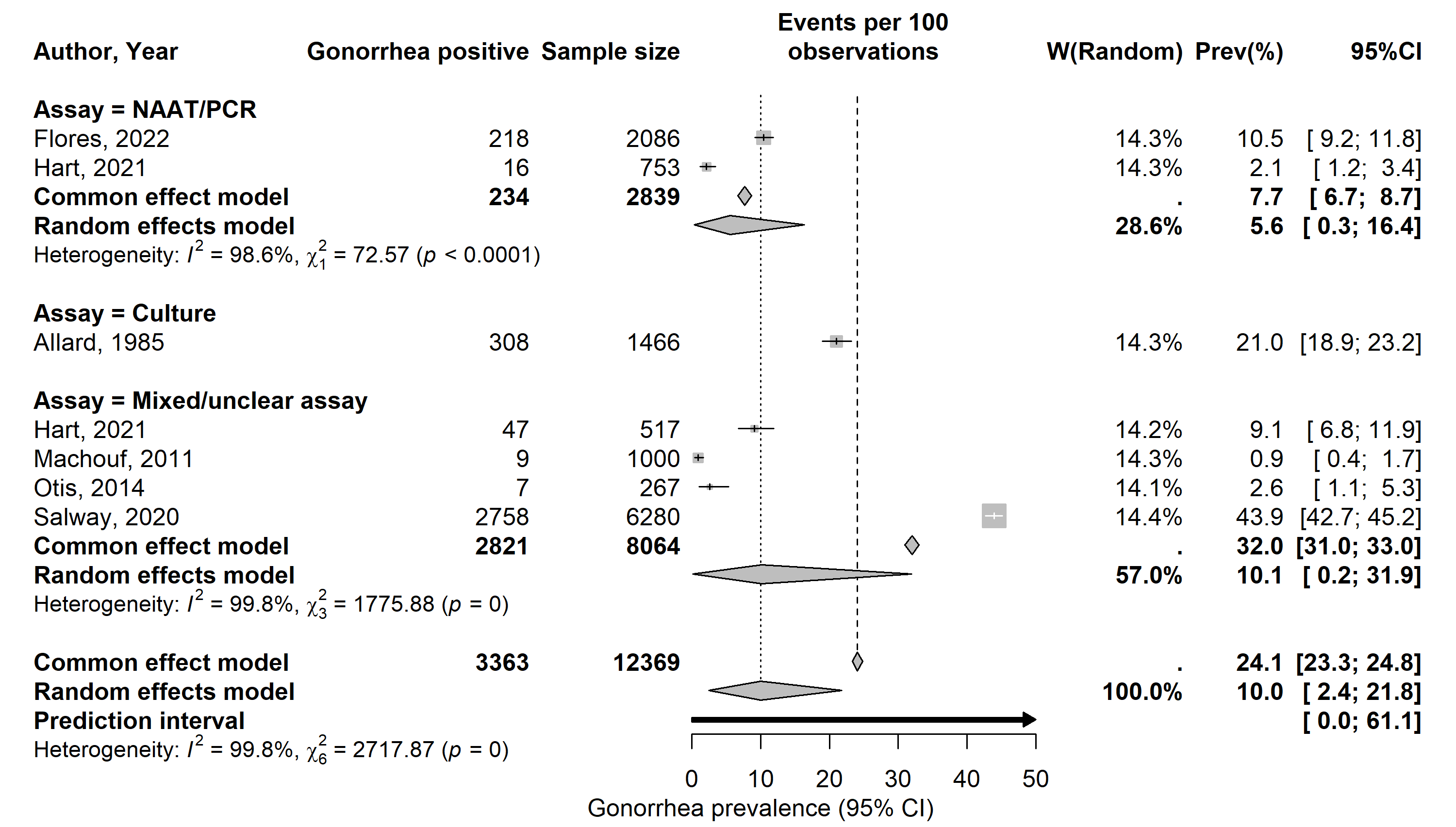
Men who have sex with men

Abbreviations: CI, Confidence interval; NAAT, Nucleic acid amplification test; PCR, Polymerase chain reaction.

1.
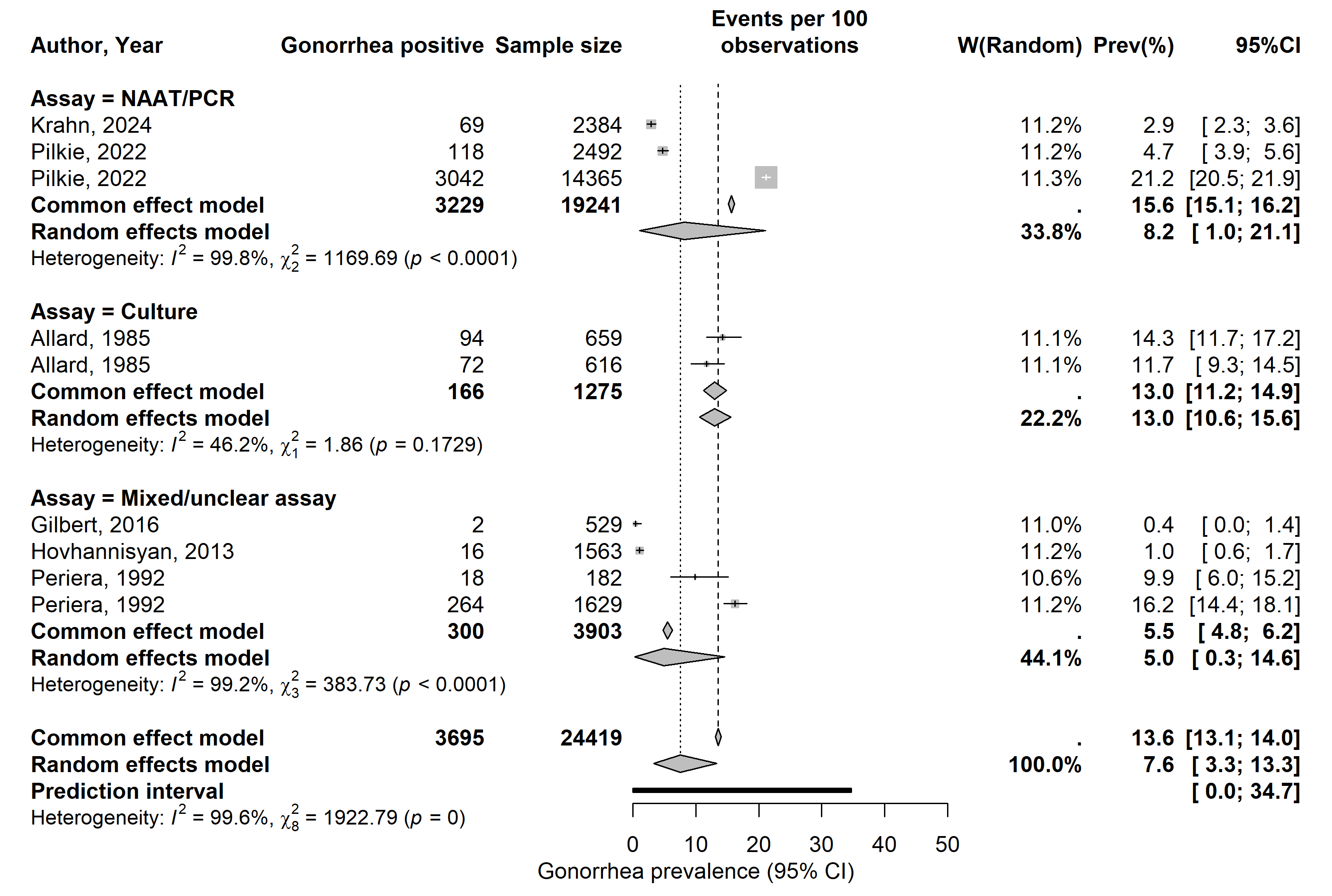
STI clinic attendees

Abbreviations: CI, Confidence interval; NAAT, Nucleic acid amplification test; PCR, Polymerase chain reaction; STI, Sexually transmitted infection.

1. Patients with confirmed or suspected STIs and related infections


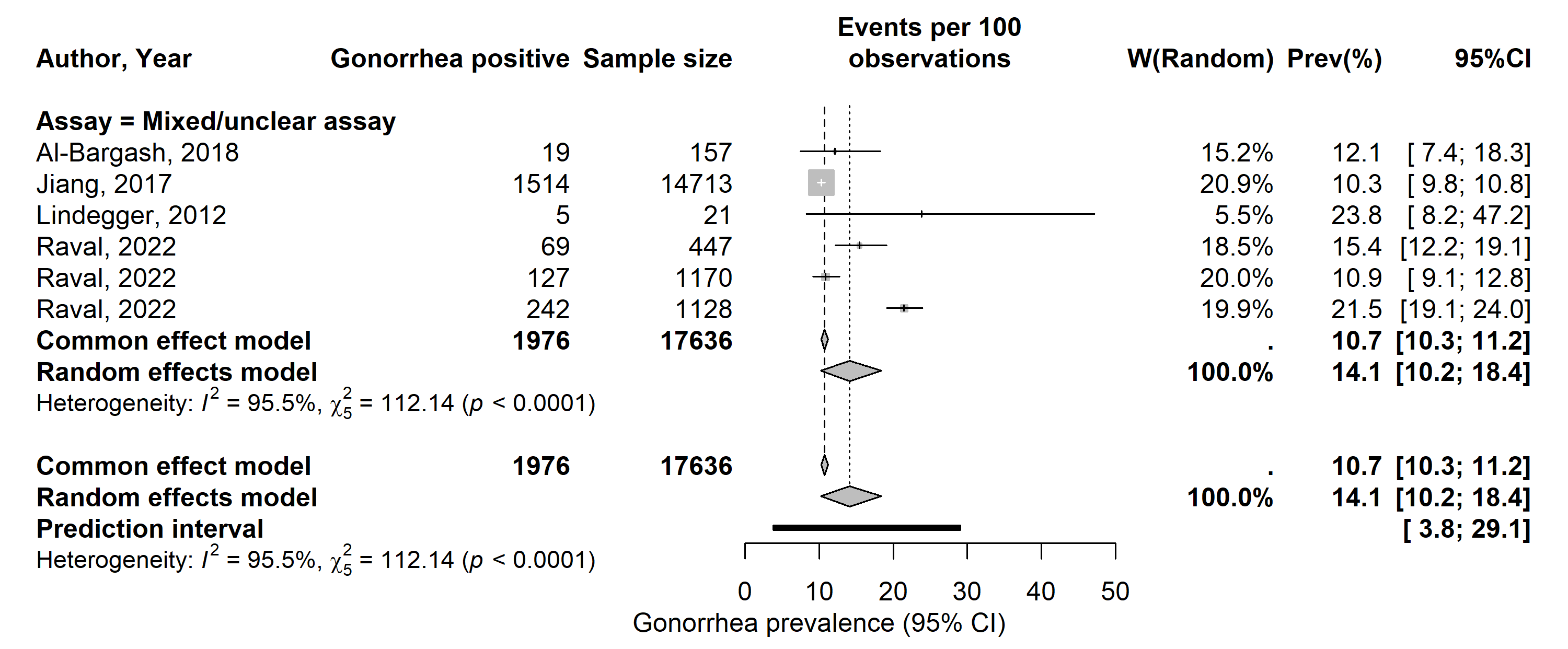
Abbreviations: CI, Confidence interval; STI, Sexually transmitted infection.

# **References**

1. Page MJ, McKenzie JE, Bossuyt PM, Boutron I, Hoffmann TC, Mulrow CD, Shamseer L, Tetzlaff JM, Akl EA, Brennan SE *et al*: **The PRISMA 2020 statement: an updated guideline for reporting systematic reviews**. *BMJ* 2021, **372**:n71.

2. Hoy D, Brooks P, Woolf A, Blyth F, March L, Bain C, Baker P, Smith E, Buchbinder R: **Assessing risk of bias in prevalence studies: modification of an existing tool and evidence of interrater agreement**. *J Clin Epidemiol* 2012, **65**(9):934-939.

3. Munn Z, Moola S, Lisy K, Riitano D, Tufanaru C: **Methodological guidance for systematic reviews of observational epidemiological studies reporting prevalence and cumulative incidence data**. *Int J Evid Based Healthc* 2015, **13**(3):147-153.

4. Furuya-Kanamori L, Barendregt JJ, Doi SAR: **A new improved graphical and quantitative method for detecting bias in meta-analysis**. *Int J Evid Based Healthc* 2018, **16**(4):195-203.
